# How to make gluten‐free friends: A quasi‐experimental study on the psychosocial benefits of celiac camps

**DOI:** 10.1111/bjhp.70027

**Published:** 2025-10-14

**Authors:** Maor Shani, Melina Böttcher

**Affiliations:** ^1^ Institute for Psychology, Osnabrück University Osnabrück Germany

**Keywords:** celiac disease, chronic illness, health‐related quality of life, illness identity, peer support, summer camps

## Abstract

**Objectives:**

Youth with Celiac Disease (CD) face social challenges, yet the benefits of specialized camps are under‐researched. This study evaluated a week‐long celiac camp's impact on social integration, illness identity, health management and health‐related quality of life (HRQOL).

**Design:**

A quasi‐experimental waitlist design was used.

**Methods:**

One hundred eleven youth with CD (ages 8–16, 65% female) were assigned to a one‐week camp in Switzerland (intervention; *n* = 66; 41 children aged 8–12 years and 25 adolescents aged 13–16 years) or a waitlist comparison group (*n* = 45; 34 children and 11 adolescents). Pre‐ and post‐camp surveys assessed psychosocial outcomes, including friendships, illness identity and peer support (adolescents only), dietary adherence, perceived competence, subjective well‐being and age‐adapted HRQOL. Linear mixed models were used to analyse intervention effects.

**Results:**

Compared to the control group, camp participation significantly increased friendships with peers with CD and promoted a more adaptive illness identity among adolescents, especially for novice attendees. No effects were found for dietary adherence or perceived competence. Unexpectedly, campers reported a short‐term worsening in the HRQOL domains of enjoyment (among children) and uncertainty (among adolescents) compared to the control, more strongly among novice campers.

**Conclusions:**

Celiac camps effectively foster social integration and positive illness identity development. However, the short‐term impact on HRQOL is complex and may reflect a challenging “re‐entry” to daily life after a highly supportive experience. These findings highlight the benefits of celiac camps while stressing the need for longitudinal research to understand and optimize their long‐term impact.

**Statement of Contribution**

**What is already known on this subject?**

- Youth with celiac disease often experience social isolation due to strict dietary restrictions.
- Medical specialty camps have been shown to improve psychosocial outcomes in chronic conditions, yet research evaluating the effectiveness of celiac camps is limited.

**What does this study add?**

- Camp participation increased friendships with peers with celiac disease, but perceived peer support did not significantly change, suggesting that social support network development may require more time.
- Illness identity rejection decreased, and enrichment increased among campers aged 13–16, particularly for novice campers.
- Some aspects of health‐related quality of life worsened after camp participation, indicating that the psychosocial impacts of such interventions are complex and may not be uniformly positive in the short term.

## INTRODUCTION

Peer relationships and social integration are crucial for adolescent self‐validation, identity formation and psychosocial adjustment (Arnett, 2013; Brown & Larson, 2009; Dahl et al., 2018; Kulandaivelu & Kohut, 2021). Research on summer camps and similar activities highlights their role in fostering self‐esteem, social competence and autonomy by offering shared experiences, supportive relationships, a sense of community, sustained adult connections, skill acquisition and active participation, all critical for personal growth (Garst et al., 2011; Schelbe et al., 2018; Sendak et al., 2018).

However, children and adolescents with chronic illnesses often face challenges that hinder their development, excluding them from typical experiences and increasing their risk of mental health issues (Coburn et al., 2019; Maslow et al., 2013; Pinquart, 2016). Celiac disease (CD), an autoimmune disorder requiring a strict, lifelong gluten‐free diet (GFD), exemplifies such conditions (Chang et al., 2022; Coburn et al., 2019). Lacking environmental support, youth with CD often face stigmatization and reduced participation in social and food‐related activities, leading to social isolation, depression and anxiety (Coburn et al., 2019; Ho et al., 2023; Meyer & Rosenblum, 2016; Olsson et al., 2009; Rosén et al., 2011). Additionally, adolescents with chronic illnesses may struggle with identity development due to their condition's limitations, affecting their ability to form a coherent sense of self (De Klerk et al., 2024; Luyckx et al., 2023; Meyer & Lamash, 2021; Wheeler et al., 2022).

Medical specialty camps, or therapeutic camps, create tailored experiences that help foster normalcy and belonging, easing the social and emotional challenges of chronic illnesses (Collins, 2020; Moola et al., 2014; Sendak et al., 2018). Research shows that these camps boost psychosocial outcomes like adaptive coping, self‐esteem and overall quality of life across various health conditions (Laing & Moules, 2014; McCarty et al., 2024). Celiac camps, specifically for youth with CD, address the difficulties posed by strict dietary restrictions. By ensuring all food is safe, these camps allow participants to enjoy communal eating without fear, reducing feelings of isolation and stigmatization and fostering positive social and emotional growth (Bongiovanni et al., 2010; Olsson et al., 2009; Sendak et al., 2018; Shani et al., 2022).

Despite their potential, empirical research on the effectiveness of celiac camps is limited. Only two studies have evaluated their impact: Bongiovanni et al. (2010) reported significant improvements in well‐being and self‐perception following a week‐long camp but lacked a control group and used non‐validated questionnaires; Shani et al. (2022) found associations between camp participation and higher disease‐specific quality of life, increased social support and lower anxiety in a cross‐sectional study, though causality cannot be inferred. These findings highlight a research gap and the need for rigorous studies to assess the efficacy of celiac camps in enhancing the psychosocial well‐being of youth with CD. Studying celiac camps is important not only for enhancing the well‐being of youth with CD but also for contributing to the broader understanding of how disease‐specific interventions can support psychosocial adjustment and self‐management skills in chronic illnesses (Berkanish et al., 2022; Månsson et al., 2022).

### Social integration and support

Celiac camps aim to create supportive environments where participants engage with peers facing similar challenges, helping reduce the isolation often experienced in chronic conditions (Evans et al., 2021; Hill & Sibthorp, 2006; Moola et al., 2014; Shani et al., 2022). Structured group activities facilitate peer bonding and provide safe spaces to share coping strategies and emotions (Moola et al., 2014; Schelbe et al., 2018). Unlike online activities, physical camps offer unique benefits for social integration through multisensory engagement and spontaneous interactions that foster deeper connections (Sibthorp et al., 2010; Warner et al., 2021). These immersive settings allow children and adolescents to develop face‐to‐face relationships and social skills in real‐world contexts, which are hard to replicate online (Bers, 2012; Walker & Venker Weidenbenner, 2019). Studies consistently show that such camps enhance social support, increase network connections and foster a sense of belonging (Conrad & Altmaier, 2009; Guastello et al., 2024; Kulandaivelu & Kohut, 2021; Laing & Moules, 2014; Moola et al., 2014). In turn, social support can reduce the chronic stress of disease management and improve health‐related quality of life (HRQOL) (Cohen & Wills, 1985; Dave et al., 2024; Kulandaivelu & Kohut, 2021). Shani et al. (2022) found that celiac camp participation was linked to increased friendships and perceived social support.

### Illness identity and identification

Celiac camps offer environments that can positively influence illness identity through shared experiences, peer support and identity exploration. Illness identity consists of four dimensions: rejection (denial), engulfment (illness dominates life), acceptance (integration of illness) and enrichment (personal growth through illness) (Oris et al., 2016). Adolescents with chronic illnesses who achieve identity commitment, like identity achievement or foreclosure, tend to adapt better and function more effectively (Luyckx et al., 2023; Vanderhaegen et al., 2024). In contrast, those in moratorium or troubled diffusion, marked by ongoing exploration and rumination without firm commitments, may struggle to integrate their illness into their identity, resulting in greater distress and lower life satisfaction (De Klerk et al., 2024; Luyckx et al., 2023; Vanderhaegen et al., 2024). Medical specialty camps may promote positive illness identity by reducing isolation and stigma, facilitating a more positive integration of CD into one's identity (Gillard & Allsop, 2016; Moola et al., 2014). Studies suggest camps stimulate personal identity awareness and contribute to a positive self‐concept (Conrad & Altmaier, 2009; Evans et al., 2021). While Shani et al. (2022) reported higher illness acceptance among celiac camp participants, more research is needed on all illness identity dimensions.

### Health management and competence

Celiac camps provide structured yet supportive environments where children and adolescents can practice self‐management skills crucial for developing independence in managing CD. Based on Self‐Determination Theory (Ryan & Deci, 2017), such camps may enhance intrinsic motivation and self‐regulatory behaviours by promoting competence, autonomy and relatedness. Peer learning, role modelling by counsellors and educational sessions can boost perceived self‐efficacy and competence in managing the GFD (Hill & Sibthorp, 2006; Maslow et al., 2013; Taylor et al., 2012). While research specific to celiac camps is limited, studies on camps for other chronic illnesses such as diabetes consistently show improvements in self‐management behaviours and treatment adherence (Barone et al., 2016; Hill & Sibthorp, 2006; Moola et al., 2014; Taylor et al., 2012).

### Health‐related quality of life and well‐being

Celiac camps may improve HRQOL and subjective well‐being (SWB) by addressing the social and emotional challenges associated with CD. Children and adolescents with CD often face impairments in HRQOL, particularly in social and emotional domains (Byström et al., 2012; Ho et al., 2023). Camps provide opportunities to connect with peers, reduce isolation and foster a sense of belonging and normalcy (Epstein et al., 2005; Moola et al., 2014). Peer interactions with those facing similar challenges can further improve HRQOL and SWB (Epstein et al., 2005; Martiniuk et al., 2014). Studies on camps for other chronic illnesses have shown improvements in psychological well‐being and feelings of support and acceptance (Epstein et al., 2005; Faith et al., 2019; Möller et al., 2021; Odar et al., 2013). In CD, Shani et al. (2022) found that celiac camp participants reported higher disease‐specific HRQOL compared to nonparticipants.

### The present study

This study addresses the limited empirical research on the effectiveness of celiac camps by evaluating the impact of week‐long residential camps on two distinct developmental cohorts: children (aged 8–12 years) and adolescents (aged 13–16 years). Using a quasi‐experimental, waitlist control group design, we examined the effects of camp participation on four key domains: (1) social integration and support, (2) illness identity, (3) health management and (4) quality of life and well‐being. Based on the foregoing literature, we hypothesized that, from pre‐ to post‐intervention, camp participants in both age groups would demonstrate significantly greater positive changes compared to their noncamper peers in the waitlist control group.

Specifically, pertaining to social integration, we hypothesized that, from pre‐ to posttest, camp participants would report a significantly greater increase in friendships with other youth with CD compared to the noncamper group. For adolescents specifically, we further predicted a significantly greater increase in perceived peer support relative to their noncamper peers. With regard to illness identity (assessed in adolescents), we hypothesized that camp participation would promote a more adaptive identity, reflected by a greater decrease in rejection and engulfment, a greater increase in acceptance and enrichment and enhanced social identification with peers with CD when compared to the changes observed in noncampers. Concerning health management, we hypothesized that camp participation would lead to a significantly greater improvement in adherence to the GFD and a greater increase in perceived competence in managing CD compared to the noncamper group. Finally, we hypothesized that camp participation would lead to significantly greater improvements in both CD‐specific HRQOL and SWB relative to the waitlist control group.

## METHOD

### Study design and ethical approval

This study used a quasi‐experimental, waitlist comparison design. The protocol was approved by the institutional ethics committee, adhering to the Declaration of Helsinki. We followed reporting guidelines for nonrandomized designs (TREND; Des Jarlais et al., 2004; see Table S2), quasi‐experimental studies (CONSORT; Schulz et al., 2010; see Table S1) and non‐randomized pilot studies (Lancaster & Thabane, 2019).

### The intervention: IG Zöliakie celiac camps

**FIGURE 1:**
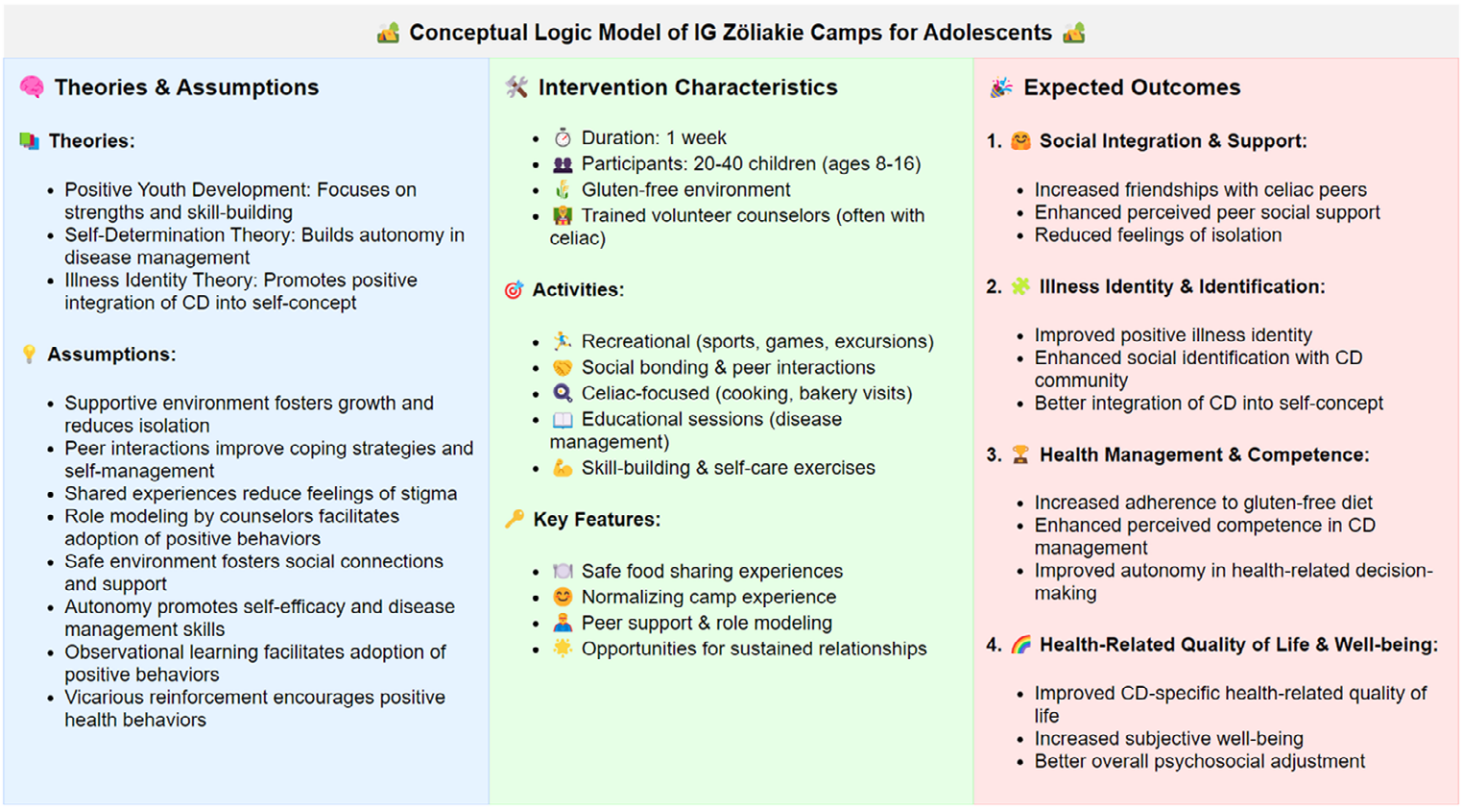
Conceptual logic model for IG Zöliakie Camps for children and adolescents.

The intervention consisted of participation in one of three week‐long residential celiac camps organized by IG Zöliakie, the Celiac Association of German‐speaking Switzerland, for youth with CD (aged 8–16). The program combines traditional summer camp activities with celiac‐specific components within a completely gluten‐free environment, allowing participants to eat communally without fear of cross‐contamination. The camps are run by trained volunteer counsellors, many of whom also have CD and act as role models. The intervention's underlying logic is to foster peer bonding, reduce disease‐related isolation and provide a supportive space for skill mastery and identity exploration (see Supporting Information for a detailed description and Figure 1 for a logic model).

### Participants and recruitment procedure

**FIGURE 2:**
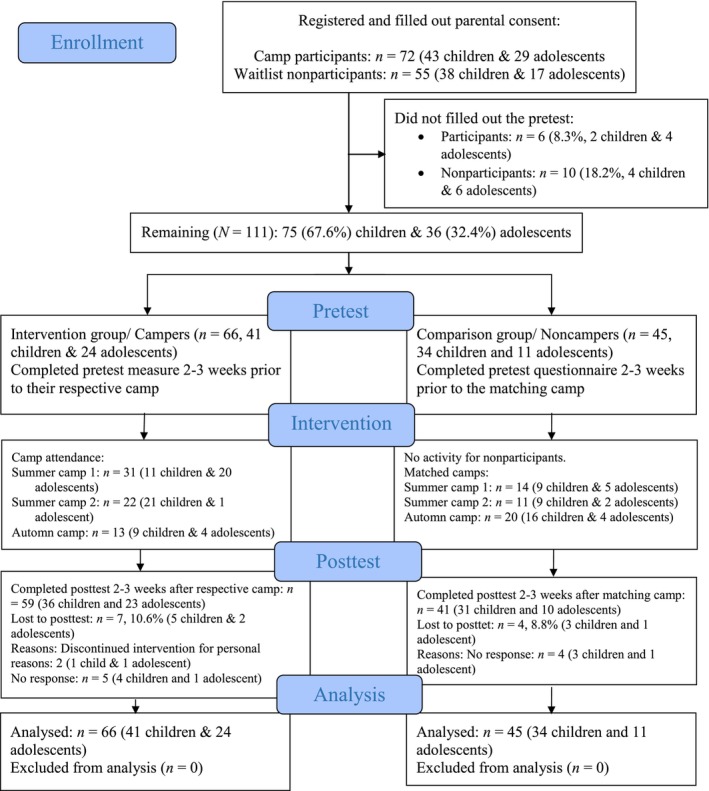
CONSORT flow diagram of the quasi‐experimental study.

Participants (*N* = 111; *M*_age_ = 11.43, *SD* = 2.19, 64.9% female) were recruited from three celiac camps in 2021 (intervention group; *n* = 66) and a proxy waitlist (comparison group; *n* = 45). Noncampers were youth interested in attending who had not registered for the current year, primarily due to limited spots. Figure 2 illustrates the participant flow. After providing consent, parents received links to online surveys. Participants completed a pretest survey 2–3 weeks before the camps and a posttest survey approximately three weeks after. Parents were encouraged to assist younger children (ages 8–12), while adolescents (13–16) completed them independently. No participants withdrew from the study.

The final sample included 75 children (41 campers, 34 noncampers) and 36 adolescents (25 campers, 11 noncampers). A significant baseline difference emerged regarding prior camp experience. Among campers, 23 (35.4%) had not attended celiac camps before (hereafter referred to as ‘novice campers’), while the remaining 42 (64.6%) had attended at least one camp before (hereafter referred to as ‘experienced campers’). In contrast, the majority of noncampers (*n =* 37, 84.1%) were novices. This disparity was particularly stark among adolescents, where 84% of campers had prior camp experience versus only 36.4% of noncampers. Additional demographic and health‐related characteristics are detailed in Table 1. Additional feedback measures for campers are summarized in an evaluation report in the Supporting Information.

**TABLE 1:** Demographic and clinical characteristics of participants by condition and age group.

Variable	Noncampers			Campers			Total sample (*n* = 111)
	Children (*n* = 34)	Adolescents (*n* = 11)	All (*n* = 45)	Children (*n* = 41)	Adolescents (*n* = 25)	All (*n* = 66)	
Gender, *n* (%)							
Male	13 (38.2%)	3 (27.3%)	16 (35.6%)	17 (41.5%)	6 (24.0%)	23 (34.8%)	39 (35.1%)
Female	21 (61.8%)	8 (72.7%)	29 (64.4%)	24 (58.4%)	19 (76.0%)	43 (65.2%)	72 (64.9%)
Camp attended/matched, *n* (%)							
Summer camp 1	9 (26.5%)	5 (45.5%)	14 (31.1%)	11 (26.8%)	20 (80.0%)	31 (47.0%)	45 (40.5%)
Summer camp 2	9 (26.5%)	2 (18.2%)	11 (24.4%)	21 (51.2%)	1 (4.0%)	22 (33.3%)	33 (29.7%)
Autumn camp	16 (47.1%)	4 (36.4%)	20 (44.4%)	9 (22.0%)	4 (16.0%)	13 (19.7%)	33 (29.7%)
Age group, *n* (%)							
8–12 years			34 (75.6%)			41 (62.1%)	75 (67.6%)
13–16 years			11 (24.4%)			25 (37.9%)	36 (32.4%)
Age, *M* (*SD*)	9.76 (1.28)	14.18 (1.08)	10.84 (2.28)	10.49 (1.16)	14.04 (0.98)	11.83 (2.05)	11.43 (2.19)
Age of CD diagnosis, *M* (*SD*)	5.09 (2.98)	7.50 (3.04)	5.62 (3.13)	5.21 (2.60)	5.80 (3.49)	5.42 (2.93)	5.50 (3.00)
Region of residence, *n* (%)							
North Switzerland	6 (17.6%)	3 (27.3%)	9 (20.0%)	4 (9.8%)	2 (8.0%)	6 (9.2%)	15 (13.6%)
Zurich	5 (14.7%)	4 (36.1%)	9 (20.0%)	13 (31.7%)	5 (20.0%)	18 (27.7%)	27 (24.5%)
Bern	7 (20.6%)	1 (9.1%)	8 (17.8%)	6 (14.6%)	7 (28.0%)	13 (20.0%)	21 (19.1%)
Central Switzerland	9 (26.5%)	1 (9.1%)	10 (22.2%)	5 (12.2%)	5 (20.0%)	10 (15.4%)	20 (18.2%)
East Switzerland	2 (5.9%)	2 (18.2%)	4 (8.9%)	3 (7.3%)	2 (8.0%)	5 (7.7%)	9 (8.2%)
Other/Abroad	5 (14.7%)		5 (11.1%)	9 (22.0%)	4 (16.0%)	13 (20.0%)	18 (16.4%)
Socio‐economic status, *M* (*SD*)	7.00 (1.02)	6.45 (0.82)	6.87 (0.99)	6.83 (1.55)	6.76 (1.30)	6.80 (1.45)	6.83 (1.28)
Have additional food restrictions?, *n* (%)							
Yes, severe	2 (5.9%)	1 (9.1%)	3 (6.7%)	4 (9.8%)	1 (4.0%)	5 (7.6%)	8 (7.2%)
Yes, mild	7 (20.6%)	2 (18.2%)	9 (20.0%)	4 (9.8%)	5 (20.0%)	9 (13.6%)	18 (16.2%)
No	25 (73.5%)	8 (72.7%)	33 (73.3%)	33 (80.5%)	19 (76,0%)	52 (78.8%)	85 (76.6%)
Have additional medical restrictions?, *n* (%)							
No	27 (79.4%)	10 (90.9%)	37 (82.2%)	32 (78.0%)	21 (84.0%)	53 (80.3%)	90 (81.1%)
Yes	7 (20.6%)	1 (9.1%)	8 (17.8%)	9 (22.0%)	3 (16.0%)	13 (19.7%)	21 (18.9%)
How often attended CD camps in the past?, *n* (%)^a^							
Never	30 (88.2%)	7 (63.6%)	37 (84.1%)	19 (47.5)%	4 (16.0%)	23 (35.4%)	60 (55.0%)
Once	2 (5.9%)	3 (27.3%)	5 (11.4%)	10 (25.0%)		10 (15.4%)	15 (13.8%)
2–3 times	1 (2.9%)	1 (9.1%)	2 (4.5%)	9 (22.5%)	10 (40.0%)	19 (29.2%)	21 (19.3%)
4 times or more			0 (0.0%)	2 (0.05%)	11 (44.0%)	13 (20.0%)	13 (11.9%)
How active in CD‐related social media and online groups?^b^, ^c^ *M* (*SD*)		0.82 (1.33)			1.60 (2.43)		1.36 (2.17)
Satisfaction with overall health^d^, *M* (*SD*)	3.85 (1.26)	3.64 (0.92)	3.80 (1.18)	3.51 (1.49)	3.80 (1.12)	3.62 (1.36)	3.69 (1.29)

*Note*: Children are 8–12 years old; Adolescents are 13–16 years old. Missing data occurred in 0 to 8 cases per variable.

Abbreviations: CD, celiac disease.

^a^ One camper in the Children group did not answer the question.

^b^ Measured only for adolescents.

^c^ Measured on a scale from 0 (*not active at all*) to 4 (*very active*).

^d^ Measured on a scale from 1 (*not satisfied at all*) to 5 (*very satisfied*).

### Measures

Details on the measures, dataset and analysis syntax are available on the Open Science Framework at https://osf.io/jub3s. All participants were assessed on celiac friendships, CDAT, perceived competence, SWB and social identification. Illness identity (IIQ) and peer support were measured only in adolescents. The CD‐specific HRQOL measure (CDPQOL) was administered in age‐adapted versions. All scales were validated German translations or underwent forward–backward translation.

Internal consistency (Cronbach's alpha) was acceptable for most scales (*α* > .70, see Table S3, for all reliability coefficients). Several subscales with lower reliability (Peer Support, CDAT and some CDPQOL subscales) were retained for comparability with prior research and because shorter scales can yield lower alphas (Hair, 2019; Taber, 2018). Results from these specific subscales are interpreted with additional caution.

#### Friendships with other children and adolescents with celiac disease

Friendships were evaluated using two items: “How many people with celiac do you know outside your family?” and “How many friends do you have who also have celiac?” Responses ranged from 1 (*none*) to 4 (6 or *more*). The use of two items helps mitigate individual differences in subjective perceptions of friendship (e.g., liking, interactions), which can vary greatly in adolescence (Kitts & Leal, 2021). The mean score was calculated, demonstrating reliable associations with other celiac‐related variables (Kitts & Leal, 2021; Shani et al., 2022).

#### Peer support

Perceived peer support was measured with three items adapted from Shani et al. (2022), such as “I enjoy talking with my friends about my celiac.” Participants rated their agreement on a 5‐point scale (1 = *completely disagree* to 5 = *completely agree*).

#### Illness identity

Illness identity was assessed using the 25‐item Illness Identity Questionnaire (IIQ; Oris et al., 2016), which measures integration of CD into self‐concept across four subscales: Rejection, Acceptance, Engulfment and Enrichment. The IIQ has been validated in adolescent CD populations (Meyer & Lamash, 2021; Shani et al., 2022).

#### Social identification

Social identification with other celiac patients was measured using the Dynamic Identity Fusion Index (DIFI; Jiménez et al., 2016), an adaptation of the Inclusion of Other in the Self (IOS) scale (Aron et al., 1992). Participants adjusted the overlap between two circles representing “self” and “group” to indicate closeness with peers who have CD. The overlap score (0–100%) reflects the degree of social identification, with strong reliability and validity established in group identification research (Jiménez et al., 2016), including in adolescents with CD (Shani et al., 2022).

#### Gluten‐free diet adherence (CDAT)

Dietary adherence was measured using the 7‐item Celiac Dietary Adherence Test (CDAT; Leffler et al., 2009). Items addressed challenges in adhering to a GFD in social situations, self‐efficacy and frequency of intentional gluten exposure, rated from 1 to 5 with diverse anchors matching the items. Lower scores indicate better adherence. Despite lower reliability estimates especially at pretest, CDAT is widely used among children and adolescents with CD and was previously found to correlate with dietitian evaluations and biomarkers (Leffler et al., 2009). Therefore, we retained it as a measure of GFD adherence.

#### Perceived competence

Perceived competence in managing a GFD was assessed using the 4‐item Gluten‐Free Diet Perceived Competence Scale (GFD‐PCS; Kipp et al., 2019). Items such as “I feel confident in my ability to maintain a gluten‐free diet” were rated on a 7‐point Likert scale (1 = *not at all true to* 7 = *very true*).

#### Paediatric celiac disease quality of life (CDPQOL)

HRQOL was measured using the Paediatric Celiac Disease Quality of Life (CDPQOL; Jordan et al., 2013), specifically developed for children and adolescents with CD. Using the CDPQOL allows for comparability with previous studies addressing similar populations and interventions (Shani et al., 2022). Two age‐adjusted versions were administered: children (aged 8–12 years) with three dimensions (Negative Emotions, School, Enjoyment; 13 items) and adolescents (aged 13–16 years) with four dimensions (Social, Uncertainty, Isolation, Limitation; 17 items). Items measured the frequency of having negative experiences in the past month (0 = *never*, 4 = *almost always*).

#### Subjective well‐being (happiness)

SWB was measured using the Fordyce Emotions Questionnaire (Fordyce, 1988), a single‐item scale where participants rated their happiness from 0 (*extremely unhappy*) to 10 (*extremely happy*). This measure is reliable and valid in adolescent populations (Diener, 1984; Kozma & Stones, 1980).

#### Demographics and health‐related variables

Demographic data included gender, age, age at CD diagnosis, socio‐economic status (MacArthur Scale of Subjective Social Status—Youth Version; Goodman et al., 2001), prior camp attendance and additional health‐related variables (see Table 1).

### Data analysis

Analyses were performed in R (version 4.4.1), with data collapsed across the three camps to enhance statistical power. A missing data analysis confirmed that data were missing completely at random (Little's MCAR test: *p* > .05).

After examining group equivalence with logistic regression and Mann–Whitney *U* tests, we employed Linear Mixed Models (LMMs) for the main analyses due to their robustness with repeated measures. Models included condition, time of testing and prior camp participation as fixed factors, with a random intercept for individuals. Measures with identical items were analysed across the full 8–16‐year sample, while age‐specific instruments were analysed within their respective groups.

A post‐hoc power analysis indicated sufficient power to detect large group differences (*d* = .60) and medium‐sized interaction effects (*η* ^2^ = .04, 98.1% power). To ensure the robustness of our primary Linear Mixed Models (LMMs), we conducted a sensitivity analysis using nonparametric Wilcoxon signed‐rank tests. This was justified by the presence of small, unequal subgroups (e.g., adolescent noncampers, *n* = 11; see Table 1), for which the parametric assumption of normally distributed residuals could not be reliably confirmed. As parametric models are not robust to such violations in very small samples, this nonparametric check was crucial (Tabachnick & Fidell, 2019). We further tested robustness in follow‐up analyses controlling for covariates, including gender, age, SES and health conditions.

## RESULTS

Descriptive statistics and Spearman bivariate correlations for the study's main variables are available in the Supporting Information (Tables S3 and S6, respectively). Furthermore, we present Spearman correlations between demographics, illness‐related and outcome variables in the Supporting Information (Tables S7.1 and S7.2 for pretest and posttest scores, respectively). Acceptability and satisfaction among campers were overall high (see evaluation report in the Supporting Information).

### Preliminary analyses

A logistic regression analysis involving the entire sample (i.e., age‐invariant measures) revealed that higher participation in past summer camps was significantly associated with being in the intervention group (odds ratio [OR] = 4.694, *p* < .001; see Table S4). Mann–Whitney *U* tests (Table S5) showed that at pretest, adolescent campers (aged 13–16 years) reported higher levels of illness identity acceptance (*p* = .008) and better CD‐specific quality of life (CDPQOL) in the social (*p* = .028) and limitation (*p* = .041) domains compared to noncampers. Due to large discrepancies in prior camp attendance between campers and noncampers, and to examine differential outcomes on novice versus experienced campers, prior camp attendance was included as an additional fixed factor in our LMMs, coded as a two‐level variable (novice vs. experienced). As a result, significant pairwise comparisons were not interpreted for cells with less than 10 participants (see Table S8).

### Effects of celiac camps

**FIGURE 3:**
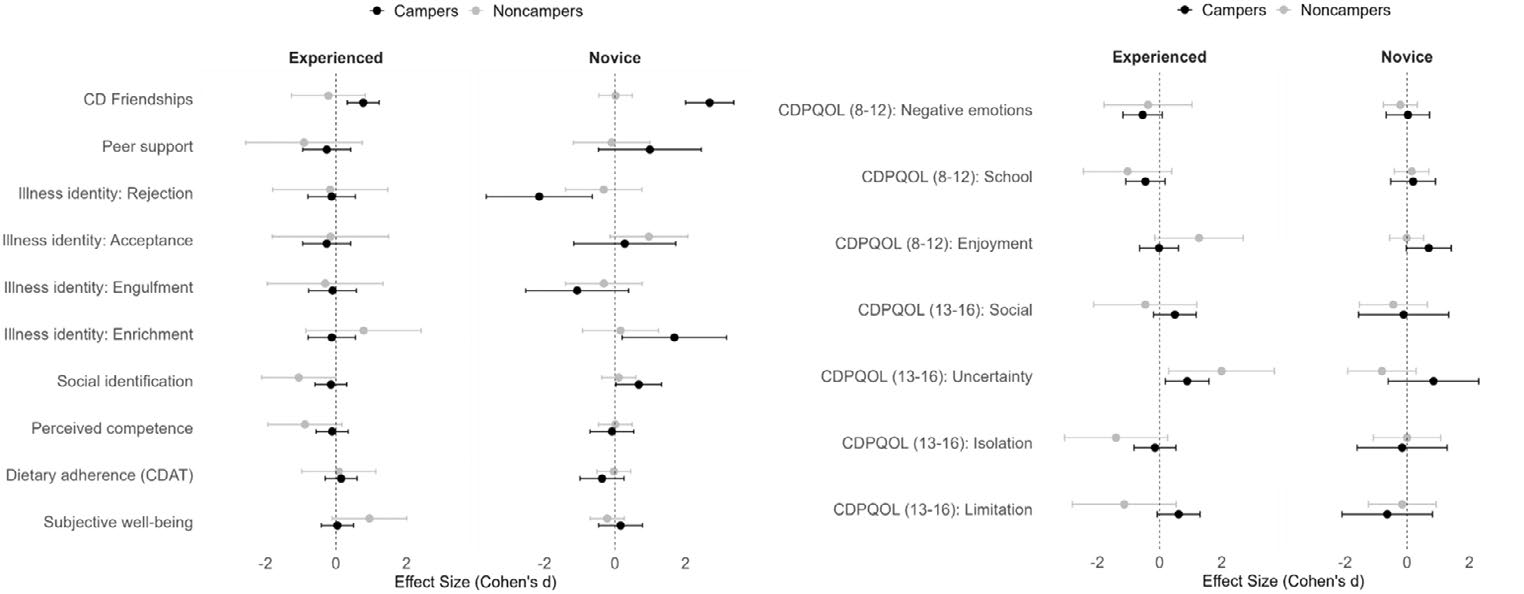
Effect sizes for pre‐post comparisons of psychosocial outcomes by camp experience and condition. Effect sizes (Cohen's *d*) are based on Estimated Marginal Means derived from linear mixed models for campers and noncampers, divided by prior camp experience (Experienced vs. Novice). Error bars represent 95% confidence intervals. Peer support and Illness identity were measured only among adolescents (aged 13–16 years). CDPQOL was measured with two‐age‐adapted versions for children (aged 8–12 years) and adolescents (aged 13–16 years) separately. See Tables 2 and 3 for full details.

Tables 2 and 3 summarize the statistics from multiple LMMs predicting social, identity‐related, self‐management and well‐being variables, as well as CDPQOL domains for each age group. Effect sizes for pre‐post effects are depicted in Figure 3. Detailed results are available in the Supporting Information (Tables S9.1–S9.17).

**TABLE 2:** Mixed‐effects linear models predicting psychosocial outcomes by prior celiac camp experience, condition and time.

Outcome	Group	Campers pretest *M* (*SD*)	Campers posttest *M* (*SD*)	Noncampers pretest *M* (*SD*)	Noncampers posttest *M* (*SD*)	Cohen's *d* for campers	Cohen's *d* for noncampers	Interaction *B* (*SE*)	*p*‐Value
CD friendships	All sample	2.3 (.12)***	3.11 (.13)	1.98 (.18)	1.93 (.19)	1.73	−.10	−1.26 (.19)	< .001
	Experienced	2.9 (.11)***	3.26 (.11)	2.13 (.24)	2.02 (.26)	.77	−.21	.80 (.33)	.018
	Novice	1.7 (.14)***	2.97 (.15)	1.84 (.11)	1.84 (.12)	2.69	.02		
Peer support (adolescents only)	All sample	3.06 (.3)	3.21 (.31)	2.81 (.36)	2.6 (.37)	.36	−.50	−.46	.229
	Experienced	3.37 (.18)	3.26 (.19)	2.42 (.42)	2.03 (.45)	−.26	−.90	.19	.724
	Novice	2.75 (.42)	3.17 (.42)	3.21 (.31)	3.17 (.3)	.99	−.10		
IIQ‐rejection (adolescents only)	All sample	2.59 (.27)**	2.07 (.28)	2.5 (.33)	2.39 (.34)	−1.14	−.24	.85 (.41)	.047
	Experienced	1.84 (.17)	1.78 (.17)	2.8 (.38)	2.72 (.42)	−.12	−.16	−.87 (.58)	.141
	Novice	3.35 (.38)**	2.35 (.38)	2.2 (.28)	2.05 (.27)	−2.15	−.32		
IIQ‐acceptance (adolescents only)	All sample	4.43 (.27)	4.44 (.28)	3.78 (.33)	3.92 (.34)	.01	.40	.25 (.32)	.445
	Experienced	4.47 (.17)	4.37 (.17)	3.85 (.38)	3.79 (.41)	−.26	−.15	−.21 (.45)	.646
	Novice	4.4 (.38)	4.5 (.38)	3.7 (.28)^†^	4.05 (.27)	.28	.96		
IIQ‐engulfment (adolescents only)	All sample	1.95 (.28)	1.71 (.29)	2.03 (.34)	1.9 (.35)	−.59	−.31	.31 (.36)	.399
	Experienced	1.67 (.17)	1.63 (.18)	2.06 (.39)	1.94 (.42)	−.10	−.31	−.39 (.51)	.442
	Novice	2.22 (.39)	1.78 (.39)	1.99 (.29)	1.86 (.28)	−1.08	−.32		
IIQ‐enrichment (adolescents only)	All sample	2.61 (.33)*	3.08 (.34)	2.74 (.4)	3.02 (.42)	.78	.47	−.91 (.52)	.094
	Experienced	2.69 (.2)	2.61 (.21)	2.77 (.46)	3.24 (.51)	−.12	.78	1.44 (.73)	.058
	Novice	2.54 (.46)*	3.54 (.46)	2.71 (.34)	2.8 (.33)	1.69	.16		
DIFI	All sample	43.23 (7.49)	50.35 (7.81)	29.45 (10.62)	16.7 (11.1)	.27	−.48	−15.23 (10.87)	.165
	Experienced	51.64 (6.3)	47.83 (6.47)	35.62 (14.5)*	7.3 (15.19)	−.14	−1.06	−9.29 (18.86)	.623
	Novice	34.82 (8.68)*	52.88 (9.14)	23.27 (6.74)	26.11 (7.01)	.67	.11		
GFD‐PCS	All sample	4.82 (.07)	4.79 (.07)	4.71 (.09)	4.61 (.1)	−.10	−.44	.02 (.10)	.812
	Experienced	4.83 (.06)	4.8 (.06)	4.66 (.13)^†^	4.44 (.13)	−.11	−.88	−.21 (.17)	.218
	Novice	4.81 (.08)	4.79 (.08)	4.77 (.06)	4.77 (.06)	−.09	.01		
CDAT	All sample	1.63 (.09)	1.6 (.09)	1.75 (.12)	1.75 (.13)	.02	−.11	.09 (.11)	.399
	Experienced	1.69 (.07)	1.73 (.08)	1.91 (.17)	1.93 (.18)	.15	.08	−.11 (.19)	.565
	Novice	1.57 (.1)	1.47 (.11)	1.58 (.08)	1.57 (.08)	−.37	−.03		
SWB	All sample	9.03 (.28)	9.1 (.28)	8.19 (.4)	8.46 (.41)	.10	.37	−.28 (.29)	.342
	Experienced	8.97 (.23)	8.99 (.24)	7.25 (.54)^†^	7.94 (.56)	.03	.69	.94 (.51)	.069
	Novice	9.09 (.32)	9.2 (.33)	9.14 (.25)	8.97 (.26)	.11	−.17		

*Note*: Peer support and IIQ outcomes were measured only for adolescents (*n* = 36). Cohen's *d* values indicate effect sizes, with positive and negative signs reflecting the direction of effects. Tukey's pairwise comparisons were conducted using Kenward‐Roger degrees of freedom. Values in parentheses are robust standard errors. Interaction terms for the “All sample” rows represent the two‐way interaction between condition and time, while interaction terms for the “Experienced” rows represent the three‐way interaction between prior camp experience, condition and time.

Abbreviations: CD, Celiac disease; CDAT, Celiac Dietary Adherence Test; CFD‐PCS, Gluten‐Free Diet Perceived Competence Scale; DIFI, Dynamic Identity Fusion Index; IIQ, Illness Identity Questionnaire; SWB, Subjective Well‐Being.

^†^ *p* < .10.

* *p* < .05.

** *p* < .01.

*** *p* < .001.

**TABLE 3:** Mixed‐effects linear models predicting CDPQOL by prior celiac camp experience, condition and time.

Outcome	Group	Campers pretest *M* (*SD*)	Campers posttest *M* (*SD*)	Noncampers pretest *M* (*SD*)	Noncampers posttest *M* (*SD*)	Cohen's *d* for campers	Cohen's *d* for noncampers	Interaction *B* (*SE*)	*p*‐Value
CDPQOL: Children aged 8–12 years (*n* = 75)									
Negative emotions	All sample	1.9 (.14)	1.79 (.15)	2.14 (.21)	2.01 (.22)	−.26	−.29	−.10 (.19)	.586
	Experienced	2 (.13)^†^	1.77 (.14)	2.36 (.31)	2.2 (.31)	−.55	−.37	−.17 (.37)	.642
	Novice	1.8 (.15)	1.81 (.15)	1.92 (.11)	1.83 (.12)	−.03	−.22		
School	All sample	1.6 (.14)	1.55 (.14)	1.89 (.21)	1.73 (.21)	−.31	−.44	−.02 (.17)	.919
	Experienced	1.73 (.13)	1.56 (.13)	2.19 (.31)	1.81 (.31)	−.46	−1.03	−.19 (.33)	.561
	Novice	1.47 (.14)	1.54 (.15)	1.6 (.11)	1.65 (.12)	.19	.15		
Enjoyment	All sample	1.32 (.13)	1.45 (.14)	1.45 (.2)	1.7 (.2)	.34	.63	−.28 (.18)	.120
	Experienced	1.39 (.13)	1.38 (.13)	1.63 (.3)^†^	2.13 (.3)	−.02	1.27	.79 (.35)	.029
	Novice	1.25 (.14)*	1.52 (.15)	1.28 (.11)	1.28 (.11)	.69	−.02		
CDPQOL: Adolescents aged 13–16 years (*n* = 36)									
Social	All sample	1.87 (.28)	1.94 (.28)	2.65 (.33)	2.51 (.34)	.19	−.45	−.11 (.28)	.209
	Experienced	1.75 (.17)	1.91 (.17)	3.04 (.38)	2.89 (.4)	.49	−.46	−.20 (.40)	.623
	Novice	2 (.38)	1.96 (.38)	2.27 (.28)	2.13 (.27)	−.11	−.45		
Uncertainty	All sample	1.74 (.28)*	2 (.28)	1.97 (.33)	2.15 (.34)	.87	.59	−.49 (.26)	.072
	Experienced	1.65 (.17)*	1.91 (.17)	2 (.39)*	2.59 (.41)	.89	1.99	.82 (.37)	.035
	Novice	1.83 (.39)	2.08 (.39)	1.95 (.28)	1.71 (.28)	.85	−.81		
Isolation	All sample	1.86 (.23)	1.8 (.24)	2.12 (.28)	1.85 (.29)	−.16	−.70	.06 (.35)	.857
	Experienced	1.78 (.14)	1.72 (.15)	2.25 (.32)^†^	1.7 (.36)	−.16	−1.40	−.55 (.48)	.263
	Novice	1.94 (.32)	1.87 (.32)	2 (.24)	2 (.23)	−.15	.00		
Limitation	All sample	2.21 (.33)	2.21 (.33)	2.97 (.4)	2.72 (.41)	−.01	−.64	.19 (.35)	.591
	Experienced	1.92 (.2)^†^	2.16 (.21)	3.67 (.46)	3.22 (.49)	.62	−1.14	−.87 (.49)	.084
	Novice	2.5 (.46)	2.25 (.46)	2.27 (.33)	2.21 (.33)	−.65	−.16		

*Note*: Cohen's *d* values indicate effect sizes, with positive and negative signs showing the direction of effects. Tukey's pairwise comparisons were conducted using Kenward–Roger degrees of freedom. Values in parentheses are robust standard errors. Interaction terms for the “All sample” rows represent the two‐way interaction between condition and time, while interaction terms for the “Experienced” rows represent the three‐way interaction between prior camping experience, condition and time.

Abbreviation: CDPQOL, Paediatric Celiac Disease Quality of Life (measured with two versions, age‐dependent).

^†^ *p* < .10.

* *p* < .05.

#### Social relationships

The analysis on the entire sample revealed a significant condition × time interaction for CD friendships, indicating that the camper group experienced a significantly greater increase in friendships with peers who have CD from pre‐ to posttest compared to the noncamper group. Accordingly, campers reported significantly more friends with CD after the camp (*d* = 1.73), with a larger effect for novice campers (*d* = 2.69) compared to experienced campers (*d* = .77; *p*s < .001). No significant interactions were found for peer support in the adolescent age group.

#### Identity

For illness identity (IIQ, adolescents only), the condition × time interaction was significant for the rejection dimension (*B* = .85, *SE* = .41, *p* = .047) and marginally significant for enrichment (*B* = −.91, *SE* = .52, *p* = .094). Planned comparisons showed that illness identity rejection decreased among campers relative to noncampers (*d* = 1.14, *p* < .01). Similarly, exploratory comparisons indicated that enrichment increased among campers, particularly novice campers (*d* = 1.69), when compared to the noncamper group. For social identification (DIFI), while the overall interaction was not significant, exploratory pairwise comparisons revealed a significant pre‐post increase specifically within the novice camper group (*d* = .67, *p*s < .05).

#### CDPQOL

Among children, the three‐way interaction was significant for the enjoyment dimension (*B* = .79, *SE* = .35, *p* = .029), with novice campers showing significantly worse enjoyment post‐intervention compared to their noncamper counterparts (*d* = .69, *p* < .05). For adolescents, significant three‐way interactions were found for uncertainty (*B* = .82, *SE* = .37, *p* = .035) and marginally for limitations (*B* = −.87, *SE* = .49, *p* = .084). Planned comparisons revealed increased uncertainty among both experienced and novice campers relative to noncampers (*d* = .87), but more strongly among experienced campers (*d* = .89, *p*s < .05) and a marginally significant increase in limitations among experienced campers (*d* = .62, *p* < .10).

#### Subjective well‐being

For both age groups, a marginally significant three‐way interaction was found (*B* = .94, *SE* = .51, *p* = .069). This suggests that while happiness levels remained stable for experienced campers, they showed a marginal increase among experienced noncampers by comparison (*n* = 8, *d* = .69, *p* < .10).

No significant intervention effects were found for dietary adherence (CDAT) and perceived competence (GFD‐PCS).

### Sensitivity and robustness analysis

Nonparametric tests (Table S10) largely corroborated the parametric findings. Notably, these within‐group tests confirmed a significant pre‐post increase in social identification (DIFI) among novice campers (V = 4, *p* = .001). They also revealed a significant pre‐post improvement in negative emotions (CDPQOL) within the experienced children's camper group (d = .55; V = 434, *p* = .02), corroborating the trends observed in the primary analyses.

LMMs including covariates (Tables S11–S13.17) were also largely consistent with uncontrolled models. For adolescents, the three‐way interactions for illness identity rejection and CDPQOL limitations became significant in the controlled model (*B* = −1.42, *SE* = .54, *p* = .014; and *B* = −1.23, *SE* = .53, *p* = .028, respectively), strengthening the conclusion that illness identity rejection decreased while perceived limitations increased among novice campers compared to noncampers.

## DISCUSSION

Addressing a gap in the literature, this study used a quasi‐experimental design to evaluate a celiac camp's impact on social integration, illness identity, health management and HRQOL in children (8–12) and adolescents (13–16) during a key developmental period (Arnett, 2013; Brown & Larson, 2009; Chang et al., 2022; Dahl et al., 2018; Maslow et al., 2013). In one of the first controlled evaluations of its kind, we introduced illness identity as a novel outcome mainly for adolescents and examined effects across developmental stages. The findings revealed that camping significantly increased friendships with peers with CD across the full sample, particularly for novices and promoted a more adaptive illness identity (decreased rejection and increased enrichment) specifically among adolescent campers. Conversely, no gains were observed in dietary adherence or perceived competence for the sample as a whole. Furthermore, the effects on HRQOL were complex and diverged by age: children who were novice campers reported a decrease in enjoyment, while adolescents reported an increase in uncertainty post‐camp.

Our most notable finding—a significant increase in friendships with peers with CD compared to noncampers, especially novices—aligns with extensive research on the social benefits of medical specialty camps (Conrad & Altmaier, 2009; Guastello et al., 2024; Hill & Sibthorp, 2006; Kulandaivelu & Kohut, 2021; Moola et al., 2014; Schelbe et al., 2018; Shani et al., 2022; Warner et al., 2021). These camps foster supportive relationships that reduce the isolation and stigmatization common in youth with CD (Bongiovanni et al., 2010; Shani et al., 2022). However, we found no significant effects on perceived peer support in adolescents, possibly due to low statistical power or insufficient time for new friendships to mature. Future work should assess the durability of these friendships (Cheung et al., 2006; Dave et al., 2024).

Among adolescents, camp participation led to a significantly greater decrease in illness identity rejection and a greater increase in enrichment compared to the noncamper group, particularly for novices, suggesting they were less likely to view their CD as a threat and more likely to find personal growth from it (Cheung et al., 2006; Meyer & Lamash, 2021; Oris et al., 2016). Exploratory analysis also revealed that novice campers showed increased social identification with peers, though this requires replication. These outcomes highlight how camps promote a more positive illness identity. Fostering these peer connections may help adolescents build support networks that improve identity, reduce exclusion and promote a positive self‐concept, all of which are linked to better health outcomes (Epstein et al., 2005; Faith et al., 2019; Maslow et al., 2013; Moola et al., 2014; Odar et al., 2013; Sendak et al., 2018).

Our results from the adolescent cohort also suggest that celiac camps may help participants move from moratorium or diffusion identity statuses towards identity achievement by reducing rumination and fostering positive commitments. Camps provide a supportive environment for exploring and integrating their illness into their broader sense of self (De Klerk et al., 2024; Luyckx et al., 2023; Vanderhaegen et al., 2024). This aligns with research showing that interventions promoting positive illness identity can mitigate the negative impact of chronic illness on identity development and improve adolescents' ability to manage their condition (Luyckx et al., 2023; Vanderhaegen et al., 2024).

Contrary to our hypotheses, we observed a significant deterioration in some HRQOL dimensions among campers relative to their noncamper counterparts. Specifically, novice child campers showed decreased enjoyment, while adolescent campers reported increased uncertainty and limitations when compared to the control group. While requiring cautious interpretation, these effects may stem from a “contrast effect” during reintegration into everyday life. The highly supportive camp atmosphere may heighten awareness of challenges at home and school (Faith et al., 2019). For children, this could manifest as reduced enjoyment, while for adolescents, peer discussions might trigger deeper contemplation of their chronic condition, leading to increased uncertainty (Byström et al., 2012; Meyer & Lamash, 2021; Olsson et al., 2009; Wheeler et al., 2022).

These mixed findings are not entirely inconsistent with the broader camp literature, which has reported inconsistent effects on HRQOL. While some studies show positive psychosocial changes post‐camp (Meltzer & Rourke, 2005; Packman et al., 2005), others report mixed or no effects (Faith et al., 2019; Kiernan et al., 2004). Odar et al.'s (2013) meta‐analysis found only a small effect (*d* = .25) on children's self‐perceptions, suggesting that the impact of camps on HRQOL may not be substantial. Moreover, the lack of improvement in HRQOL dimensions might be due to the camp prompting adolescents to confront the enduring challenges of managing CD, despite enhancing social integration and promoting positive illness identity.

Finally, the lack of improvement in dietary adherence and competence contrasts with camps for other conditions like diabetes where self‐management typically improves (Barone et al., 2016; Cheung et al., 2006; Collins, 2020; Moola et al., 2014; Taylor et al., 2012). This discrepancy may be explained by high baseline adherence (a ceiling effect; Shani et al., 2022), the camp's primary focus on social connections over dietary skills (Hill & Sibthorp, 2006; Sibthorp et al., 2010) or its insufficient duration. Furthermore, measurement limitations, like the low reliability of the CDAT in our sample, could also limit the validity of these findings.

### Limitations, contributions and future research

Several limitations of the present study warrant careful consideration. First, the quasi‐experimental design, lacking random assignment, introduces potential selection biases, thereby limiting our ability to draw causal inferences (Shadish et al., 2002). This is evidenced by the significant baseline difference in prior camp attendance between our groups. Second, the small sample size, particularly within the adolescent noncamper subgroup (*n* = 11), restricted statistical power to detect smaller effects and examine important moderators such as gender or prior camp experience. Third, the reliance on self‐reported data may introduce response biases (Odar et al., 2013).

A key limitation is the short, single follow‐up period of three to four weeks post‐camp. This timing may not capture the stable, long‐term impact of the intervention, but rather a transient “re‐entry” effect as youth readjust to their home environment. As research on other chronic illness camps suggests, some psychosocial benefits may take longer to consolidate and manifest, while other effects may only emerge over an extended period (Faith et al., 2019; Moola et al., 2014). Future research must incorporate extended longitudinal follow‐ups (e.g., 6 and 12 months) to map the full trajectory of camp effects and determine if the observed changes are temporary adjustments or lasting outcomes.

Furthermore, the appropriateness of the selected measures for this specific psychosocial intervention requires critical evaluation. The lack of change on the Celiac Dietary Adherence Test (CDAT), for instance, may reflect a “measure‐concept mismatch”, as the camp was a psychosocial intervention, not a dietary training program. This is compounded by the measure's low reliability in our sample. Similarly, the paradoxical HRQOL findings must be interpreted with caution due to the low internal consistency of the specific CDPQOL subscales involved. While the CDPQOL is a validated instrument, the poor performance of these subscales in our sample means the findings could be genuine psychological effects or measurement artefacts. Future studies should consider using alternative or supplementary measures to corroborate findings, such as the Celiac Disease DUX (CDDUX), which has also been validated among children and adolescents in a European context (Meyer & Shani, 2022; Thompson et al., 2022).

Finally, the study's focus on a single camp organization in Switzerland limits the generalizability of our findings to other settings and populations (Dave et al., 2024). To address these limitations, future research should prioritize randomized controlled trials (RCTs) to enhance causal inference, as well as larger, multi‐site studies to increase sample sizes and statistical power (Guastello et al., 2024). Incorporating qualitative methodologies, such as interviews, could also provide deeper insights into the mechanisms through which camps influence psychosocial outcomes and the camper experience (Egmose et al., 2024).

Despite its limitations, this study makes several key contributions. It offers the first quasi‐experimental evaluation of celiac camps with a waitlist control group and validated measures, offering insights on the experiences of youth with CD in a contemporary European context. It highlights the benefits of in‐person interactions as psychosocial interventions, countering digital‐only trends. The study also contributes to the medical specialty camp literature by revealing complex effects on HRQOL and introducing illness identity and social identification as novel outcomes. It pioneered exploration of how psychosocial interventions shape illness identity, helping adolescents integrate their illness into broader life domains (Luyckx et al., 2023; Oris et al., 2016). Lastly, it provides valuable insights for interventions for youth with chronic food‐related illnesses, showing that celiac camps offer both normative developmental experiences and improved coping strategies.

### Practical implications

A key practical implication is the value of integrating physical camps into therapeutic interventions for youth with CD and similar chronic illnesses. These camps provide a safe, inclusive environment for interacting with peers who face similar challenges, which fosters social connections, reduces isolation and enhances psychosocial well‐being. While recent research has focused on digital interventions (Berkanish et al., 2022; Månsson et al., 2022), celiac camps offer unique advantages through rich, multisensory engagement and spontaneous social interactions that facilitate healthy development.

However, our findings suggest practitioners must be aware of a potential “re‐entry” challenge, where the contrast between the supportive camp environment and a less accommodating home or school life may cause a short‐term decrease in HRQOL. A critical implication is for camp facilitators to prepare participants for this transition by processing the “returning home” experience before camp ends. To ensure the sustainability of psychosocial benefits, ongoing support beyond the camp is needed. Implementing structured follow‐up actions, such as online forums or periodic meetings, can help maintain gains from camp and mitigate potential negative effects of returning home. Integrating peer support networks within healthcare systems can extend the positive impact, possibly contributing to increased HRQOL over time.

## CONCLUSION

In conclusion, celiac camps appear to be a powerful intervention for fostering peer friendships and promoting a more positive illness identity among youth with CD, particularly for adolescents. However, their impact is complex and not uniformly positive in the immediate aftermath. The observed null effects on health management and the paradoxical worsening of specific HRQOL domains suggest that benefits may come with short‐term psychosocial adjustments. These findings challenge the field to look beyond simple positive outcomes and investigate the complex psychological processes that occur when youth return from a peak experience to daily life. A multifaceted, person‐centred approach that combines in‐person camp experiences with structured follow‐up support is likely needed to optimize the benefits of these valuable programs and contribute to the long‐term well‐being of youth living with CD.

## AUTHOR CONTRIBUTIONS

**Maor Shani:** Conceptualization; project administration; investigation; methodology; software; formal analysis; writing – original draft. **Melina Böttcher:** Conceptualization; project administration; software; writing – review and editing.

## FUNDING INFORMATION

The authors declare no funding resources.

## CONFLICT OF INTEREST STATEMENT

The authors declare that there is no conflict of interests.

## ETHICS STATEMENT

This study was approved by the ethics committee of the authors' institute and was performed in accordance with the ethical standards laid down in the 1964 Declaration of Helsinki and its later amendments.

## PARTICIPANTS CONSENT STATEMENT

All participants under 18 obtained signed parental consent for participating in the study and, in addition, gave their own informed consent prior to their participation. No details that might disclose their identity were obtained, and the dataset was fully anonymized after matching pretest and posttest questionnaires.

## OPEN SCIENCE STATEMENT

We provide our code and analysis results as well as extensive Supporting Information on the Open Science Framework project page: https://osf.io/jub3s.

## Supporting information


Data S1.

## Data Availability

The data that support the findings of this study are openly available in OSF at https://osf.io/jub3s/, reference number DOI 10.17605/OSF.IO/JUB3S.

## References

[bjhp70027-bib-0002] Arnett, J. J. (2013). Adolescence and emerging adulthood: A cultural approach (5th ed., internat. ed.). Pearson.

[bjhp70027-bib-0003] Aron, A. , Aron, E. N. , & Smollan, D. (1992). Inclusion of other in the self scale and the structure of interpersonal closeness. Journal of Personality and Social Psychology, 63(4), 596–612. 10.1037/0022-3514.63.4.596

[bjhp70027-bib-0004] Barone, M. T. U. , Vivolo, M. A. , & Madden, P. B. (2016). Are diabetes camps effective? Diabetes Research and Clinical Practice, 114, 15–22. 10.1016/j.diabres.2016.01.013 27103364

[bjhp70027-bib-0005] Berkanish, P. , Pan, S. , Viola, A. , Rademaker, Q. , & Devine, K. A. (2022). Technology‐based peer support interventions for adolescents with chronic illness: A systematic review. Journal of Clinical Psychology in Medical Settings, 29(4), 911–942. 10.1007/s10880-022-09853-0 35147830 PMC8853345

[bjhp70027-bib-0006] Bers, M. U. (2012). Designing digital experiences for positive youth development: From playpen to playground (1st ed.). Oxford University Press, Incorporated.

[bjhp70027-bib-0007] Bongiovanni, T. R. S. , Clark, A. L. , Garnett, E. A. , Wojcicki, J. M. , & Heyman, M. B. (2010). Impact of gluten‐free camp on quality of life of children and adolescents with celiac disease. Pediatrics, 125(3), e525–e529. 10.1542/peds.2009-1862 20156892 PMC3334335

[bjhp70027-bib-0008] Brown, B. B. , & Larson, J. (2009). Peer relationships in adolescence. In R. M. Lerner & L. Steinberg (Eds.), Handbook of adolescent psychology (1st ed.). Wiley. 10.1002/9780470479193.adlpsy002004

[bjhp70027-bib-0009] Byström, I.‐M. , Hollén, E. , Fälth‐Magnusson, K. , & Johansson, A. (2012). Health‐related quality of life in children and adolescents with celiac disease: From the perspectives of children and parents. Gastroenterology Research and Practice, 2012, 1–6. 10.1155/2012/986475 PMC3324145 22548054

[bjhp70027-bib-0010] Chang, D. , O'Shea, D. , Therrien, A. , & Silvester, J. A. (2022). Review article: Becoming and being coeliac—special considerations for childhood, adolescence and beyond. Alimentary Pharmacology & Therapeutics, 56(S1), S73–S85. 10.1111/apt.16851 35815825 PMC9441244

[bjhp70027-bib-0011] Cheung, R. , Cureton, V. Y. , & Canham, D. L. (2006). Quality of life in adolescents with type 1 diabetes who participate in diabetes camp. The Journal of School Nursing, 22(1), 53–58. 10.1177/10598405060220010901 16435931

[bjhp70027-bib-0012] Coburn, S. S. , Puppa, E. L. , & Blanchard, S. (2019). Psychological comorbidities in childhood celiac disease: A systematic review. Journal of Pediatric Gastroenterology and Nutrition, 69(2), E25–E33. 10.1097/MPG.0000000000002407 31149937

[bjhp70027-bib-0013] Cohen, S. , & Wills, T. A. (1985). Stress, social support, and the buffering hypothesis. Psychological Bulletin, 98(2), 310–357. 10.1037/0033-2909.98.2.310 3901065

[bjhp70027-bib-0014] Collins, T. M. (2020). Medical specialty camps: A holistic approach to assist in the management of diabetes. *Dissertation Abstracts International Section A: Humanities and Social Sciences*, *81*(7‐A). 10.25777/a7bd-0c36

[bjhp70027-bib-0015] Conrad, A. L. , & Altmaier, E. M. (2009). Specialized summer camp for children with cancer: Social support and adjustment. Journal of Pediatric Oncology Nursing, 26(3), 150–157. 10.1177/1043454209334418 19414831

[bjhp70027-bib-0016] Dahl, R. E. , Allen, N. B. , Wilbrecht, L. , & Suleiman, A. B. (2018). Importance of investing in adolescence from a developmental science perspective. Nature, 554(7693), 441–450. 10.1038/nature25770 29469094

[bjhp70027-bib-0017] Dave, S. , Kim, S. C. , Beaver, S. , Hasimoglu, Y. G. , Katz, I. , Luedke, H. , Yandulskaya, A. S. , & Sharma, N. (2024). Peer support in adolescents and young adults with chronic or rare conditions in northern America and Europe: Targeted literature review. Journal of Pediatric Nursing, 78, e31–e40. 10.1016/j.pedn.2024.06.001 38964964

[bjhp70027-bib-0018] De Klerk, E. , Deacon, E. , & Van Rensburg, E. (2024). Reviewing identity development in young people living with type 1 diabetes mellitus. Journal of Adolescence, 97, 73–84. 10.1002/jad.12412 39327821 PMC11701391

[bjhp70027-bib-0019] Des Jarlais, D. C. , Lyles, C. , Crepaz, N. , & the TREND Group . (2004). Improving the reporting quality of nonrandomized evaluations of behavioral and public health interventions: The TREND statement. American Journal of Public Health, 94(3), 361–366. 10.2105/AJPH.94.3.361 14998794 PMC1448256

[bjhp70027-bib-0020] Diener, E. (1984). Subjective well‐being. Psychological Bulletin, 95(3), 542–575. 10.1037/0033-2909.95.3.542 6399758

[bjhp70027-bib-0021] Egmose, B. , Huniche, L. , Bindslev‐Jensen, C. , Nielsen, D. S. , & Mørtz, C. G. (2024). Exploring young adults' experiences with food allergy during their teenage years: A practice research study. Scandinavian Journal of Caring Sciences, 38(4), 844–853. 10.1111/scs.13283 38988314

[bjhp70027-bib-0022] Epstein, I. , Stinson, J. , & Stevens, B. (2005). The effects of camp on health‐related quality of life in children with chronic illnesses: A review of the literature. Journal of Pediatric Oncology Nursing, 22(2), 89–103. 10.1177/1043454204273881 15695351

[bjhp70027-bib-0023] Evans, M. , Daaleman, T. , & Fisher, E. B. (2021). Peer support for chronic medical conditions. In J. D. Avery (Ed.), Peer support in medicine (pp. 49–69). Springer International Publishing. 10.1007/978-3-030-58660-7_3

[bjhp70027-bib-0024] Faith, M. A. , Mayes, S. , Pratt, C. D. , & Carter, C. (2019). Improvements in hope and beliefs about illness following a summer camp for youth with chronic illnesses. Journal of Pediatric Nursing, 44, 56–62. 10.1016/j.pedn.2018.10.016 30683282

[bjhp70027-bib-0025] Fordyce, M. W. (1988). A review of research on the happiness measures: A sixty second index of happiness and mental health. Social Indicators Research, 20(4), 355–381. 10.1007/BF00302333

[bjhp70027-bib-0026] Garst, B. A. , Browne, L. P. , & Bialeschki, M. D. (2011). Youth development and the camp experience. New Directions for Youth Development, 2011(130), 73–87. 10.1002/yd.398 21786411

[bjhp70027-bib-0027] Gillard, A. , & Allsop, J. (2016). Camp experiences in the lives of adolescents with serious illnesses. Children and Youth Services Review, 65, 112–119. 10.1016/j.childyouth.2016.04.001

[bjhp70027-bib-0028] Goodman, E. , Adler, N. E. , Kawachi, I. , Frazier, A. L. , Huang, B. , & Colditz, G. A. (2001). Adolescents' perceptions of social status: Development and evaluation of a new indicator. Pediatrics, 108(2), e31. 10.1542/peds.108.2.e31 11483841

[bjhp70027-bib-0029] Guastello, A. D. , Barthle‐Herrera, M. A. , Downing, S. , Mirhosseini, T. , Valko, A. , & McNamara, J. P. H. (2024). Using summer camps as opportunities to provide brief interventions. In T. E. Davis Iii & E. A. Storch (Eds.), Brief CBT and science‐based tailoring for children, adolescents, and young adults (pp. 135–150). Springer Nature. 10.1007/978-3-031-60746-2_8

[bjhp70027-bib-0030] Hair, J. F. (2019). Multivariate data analysis (8th ed.). Cengage.

[bjhp70027-bib-0031] Hill, E. , & Sibthorp, J. (2006). Autonomy support at diabetes camp: A self determination theory approach to therapeutic recreation. Therapeutic Recreation Journal, 40(2), 107–125.

[bjhp70027-bib-0032] Ho, W. H. J. , Atkinson, E. L. , & David, A. L. (2023). Examining the psychosocial well‐being of children and adolescents with coeliac disease: A systematic review. Journal of Pediatric Gastroenterology and Nutrition, 76(1), e1–e14. 10.1097/MPG.0000000000003652 36573999

[bjhp70027-bib-0033] Jiménez, J. , Gómez, Á. , Buhrmester, M. D. , Vázquez, A. , Whitehouse, H. , & Swann, W. B. (2016). The dynamic identity fusion index: A new continuous measure of identity fusion for web‐based questionnaires. Social Science Computer Review, 34(2), 215–228. 10.1177/0894439314566178

[bjhp70027-bib-0034] Jordan, N. E. , Li, Y. , Magrini, D. , Simpson, S. , Reilly, N. R. , Defelice, A. R. , Sockolow, R. , & Green, P. H. R. (2013). Development and validation of a celiac disease quality of life instrument for north American children. Journal of Pediatric Gastroenterology and Nutrition, 57(4), 477–486. 10.1097/MPG.0b013e31829b68a1 23689265

[bjhp70027-bib-0035] Kiernan, G. , Gormley, M. , & MacLachlan, M. (2004). Outcomes associated with participation in a therapeutic recreation camping programme for children from 15 European countries: Data from the ‘Barretstown studies’. Social Science & Medicine, 59(5), 903–913. 10.1016/j.socscimed.2003.12.010 15186893

[bjhp70027-bib-0036] Kipp, T. O. , Husby, S. , & Skjerning, H. (2019). The large majority of coeliacs have a high degree of perceived dietary competence. Scandinavian Journal of Gastroenterology, 54(12), 1452–1457. 10.1080/00365521.2019.1690039 31738623

[bjhp70027-bib-0037] Kitts, J. A. , & Leal, D. F. (2021). What is(n't) a friend? Dimensions of the friendship concept among adolescents. Social Networks, 66, 161–170. 10.1016/j.socnet.2021.01.004 34012218 PMC8128148

[bjhp70027-bib-0038] Kozma, A. , & Stones, M. J. (1980). The measurement of happiness: Development of the memorial university of Newfoundland scale of happiness (MUNSH). Journal of Gerontology, 35(6), 906–912. 10.1093/geronj/35.6.906 7440930

[bjhp70027-bib-0039] Kulandaivelu, Y. , & Kohut, S. A. (2021). Peer support for adolescents with chronic illness. In J. D. Avery (Ed.), Peer support in medicine (pp. 95–113). Springer International Publishing. 10.1007/978-3-030-58660-7_5

[bjhp70027-bib-0040] Laing, C. M. , & Moules, N. J. (2014). Children's cancer camps: A sense of community, a sense of family. Journal of Family Nursing, 20(2), 185–203. 10.1177/1074840714520717 24492974

[bjhp70027-bib-0041] Lancaster, G. A. , & Thabane, L. (2019). Guidelines for reporting non‐randomised pilot and feasibility studies. Pilot and Feasibility Studies, 5(1), 114. 10.1186/s40814-019-0499-1 31608150 PMC6778655

[bjhp70027-bib-0042] Leffler, D. A. , Dennis, M. , Edwards George, J. B. , Jamma, S. , Magge, S. , Cook, E. F. , Schuppan, D. , & Kelly, C. P. (2009). A simple validated gluten‐free diet adherence survey for adults with celiac disease. Clinical Gastroenterology and Hepatology, 7(5), 530–536.e2. 10.1016/j.cgh.2008.12.032 19268725

[bjhp70027-bib-0043] Luyckx, K. , Vanderhaegen, J. , Raemen, L. , & Claes, L. (2023). Identity formation in adolescence and emerging adulthood: A process‐oriented and applied perspective. European Journal of Developmental Psychology, 22(2), 168–187. 10.1080/17405629.2023.2250128

[bjhp70027-bib-0044] Månsson, A. L. , Meijer‐Boekel, C. , & Mårild, K. (2022). Utilization and effectiveness of eHealth technology in the follow‐up of celiac disease: A systematic review. Journal of Pediatric Gastroenterology and Nutrition, 74(6), 812–818. 10.1097/MPG.0000000000003423 35849504

[bjhp70027-bib-0045] Martiniuk, A. , Silva, M. , Amylon, M. , & Barr, R. (2014). Camp programs for children with cancer and their families: Review of research progress over the past decade. Pediatric Blood & Cancer, 61(5), 778–787. 10.1002/pbc.24912 24395392

[bjhp70027-bib-0046] Maslow, G. , Adams, C. , Willis, M. , Neukirch, J. , Herts, K. , Froehlich, W. , Calleson, D. , & Rickerby, M. (2013). An evaluation of a positive youth development program for adolescents with chronic illness. Journal of Adolescent Health, 52(2), 179–185. 10.1016/j.jadohealth.2012.06.020 23332482

[bjhp70027-bib-0047] McCarty, R. J. , Downing, S. T. , Guastello, A. D. , Lazaroe, L. M. , Ordway, A. R. , MirHosseini, T. , Barthle‐Herrera, M. A. , Cooke, D. L. , Mathews, C. A. , & McNamara, J. P. H. (2024). Implementation and preliminary outcomes of an exposure‐based summer camp for pediatric OCD and anxiety. Behavior Therapy, 55(3), 543–557. 10.1016/j.beth.2023.08.006 38670667

[bjhp70027-bib-0048] Meltzer, L. J. , & Rourke, M. T. (2005). Oncology summer camp: Benefits of social comparison. Children's Health Care, 34(4), 305–314. 10.1207/s15326888chc3404_5

[bjhp70027-bib-0049] Meyer, S. , & Lamash, L. (2021). Illness identity in adolescents with celiac disease. Journal of Pediatric Gastroenterology and Nutrition, 72(2), e42–e47. 10.1097/MPG.0000000000002946 32932383

[bjhp70027-bib-0050] Meyer, S. , & Rosenblum, S. (2016). Children with celiac disease: Health‐related quality of life and leisure participation. American Journal of Occupational Therapy, 70(6), 7006220010p1–7006220010p8. 10.5014/ajot.2016.020594 27767940

[bjhp70027-bib-0051] Meyer, S. , & Shani, M. (2022). Structural validation and dyadic child–parent measurement invariance of the celiac disease quality of life questionnaire. European Journal of Gastroenterology & Hepatology, 34(1), 39–47. 10.1097/MEG.0000000000002051 33470699

[bjhp70027-bib-0052] Möller, S. P. , Hayes, B. , Wilding, H. , Apputhurai, P. , Tye‐Din, J. A. , & Knowles, S. R. (2021). Systematic review: Exploration of the impact of psychosocial factors on quality of life in adults living with coeliac disease. Journal of Psychosomatic Research, 147, 110537. 10.1016/j.jpsychores.2021.110537 34139581

[bjhp70027-bib-0053] Moola, F. J. , Faulkner, G. E. J. , White, L. , & Kirsh, J. A. (2014). The psychological and social impact of camp for children with chronic illnesses: A systematic review update. Child: Care, Health and Development, 40(5), 615–631. 10.1111/cch.12114 25250399

[bjhp70027-bib-0054] Odar, C. , Canter, K. S. , & Roberts, M. C. (2013). Relationship between camp attendance and self‐perceptions in children with chronic health conditions: A meta‐analysis. Journal of Pediatric Psychology, 38(4), 398–411. 10.1093/jpepsy/jss176 23381729

[bjhp70027-bib-0055] Olsson, C. , Lyon, P. , Hörnell, A. , Ivarsson, A. , & Sydner, Y. M. (2009). Food that makes you different: The stigma experienced by adolescents with celiac disease. Qualitative Health Research, 19(7), 976–984. 10.1177/1049732309338722 19556403

[bjhp70027-bib-0056] Oris, L. , Rassart, J. , Prikken, S. , Verschueren, M. , Goubert, L. , Moons, P. , Berg, C. A. , Weets, I. , & Luyckx, K. (2016). Illness identity in adolescents and emerging adults with type 1 diabetes: Introducing the illness identity questionnaire. Diabetes Care, 39(5), 757–763. 10.2337/dc15-2559 26989179

[bjhp70027-bib-0057] Packman, W. , Greenhalgh, J. , Chesterman, B. , Shaffer, T. , Fine, J. , Vanzutphen, K. , Golan, R. , & Amylon, M. D. (2005). Siblings of pediatric cancer patients: The quantitative and qualitative nature of quality of life. Journal of Psychosocial Oncology, 23(1), 87–108. 10.1300/J077v23n01_06 16492646

[bjhp70027-bib-0058] Pinquart, M. (2016). Systematic review: Bullying involvement of children with and without chronic physical illness and/or physical/sensory disability: A meta‐analytic comparison with healthy/nondisabled peers. Journal of Pediatric Psychology, 42, jsw081. 10.1093/jpepsy/jsw081 27784727

[bjhp70027-bib-0059] Rosén, A. , Ivarsson, A. , Nordyke, K. , Karlsson, E. , Carlsson, A. , Danielsson, L. , Högberg, L. , & Emmelin, M. (2011). Balancing health benefits and social sacrifices: A qualitative study of how screening‐detected celiac disease impacts adolescents' quality of life. BMC Pediatrics, 11(1), 32. 10.1186/1471-2431-11-32 21569235 PMC3120678

[bjhp70027-bib-0060] Ryan, R. M. , & Deci, E. L. (2017). Self‐determination theory: Basic psychological needs in motivation, development, and wellness. Guilford Press.

[bjhp70027-bib-0061] Schelbe, L. , Deichen Hansen, M. E. , France, V. L. , Rony, M. , & Twichell, K. E. (2018). Does camp make a difference?: Camp counselors' perceptions of how camp impacted youth. Children and Youth Services Review, 93, 441–450. 10.1016/j.childyouth.2018.08.022

[bjhp70027-bib-0062] Schulz, K. F. , Altman, D. G. , & Moher, D. (2010). CONSORT 2010 statement: Updated guidelines for reporting parallel group randomised trials. Journal of Pharmacology and Pharmacotherapeutics, 1(2), 100–107. 10.4103/0976-500X.72352 21350618 PMC3043330

[bjhp70027-bib-0063] Sendak, M. D. , Schilstra, C. , Tye, E. , Brotkin, S. , & Maslow, G. (2018). Positive youth development at camps for youth with chronic illness: A systematic review of the literature. Journal of Youth Development, 13(1–2), 201–215.

[bjhp70027-bib-0064] Shadish, W. R. , Cook, T. D. , & Campbell, D. T. (2002). Experimental and quasi‐experimental designs for generalized causal inference. Wadsworth Cengage Learning.

[bjhp70027-bib-0066] Shani, M. , Kraft, L. , Müller, M. , & Boehnke, K. (2022). The potential benefits of camps for children and adolescents with celiac disease on social support, illness acceptance, and health‐related quality of life. Journal of Health Psychology, 27(7), 1635–1645. 10.1177/1359105320968142 33198516

[bjhp70027-bib-0067] Sibthorp, J. , Browne, L. , & Bialeschki, M. D. (2010). Measuring positive youth development at summer camp: Problem solving and camp connectedness. Research in Outdoor Education, 10(1), 1–12. 10.1353/roe.2010.0002

[bjhp70027-bib-0068] Tabachnick, B. , & Fidell, L. (2019). Using multivariate statistics (7th ed.). Pearson.

[bjhp70027-bib-0069] Taber, K. S. (2018). The use of Cronbach's alpha when developing and reporting research instruments in science education. Research in Science Education, 48(6), 1273–1296. 10.1007/s11165-016-9602-2

[bjhp70027-bib-0070] Taylor, J. , Piatt, J. , Hill, E. , & Malcolm, T. (2012). Diabetes camps and self‐determination theory: Controlling glycemic level in youth with type 1 diabetes. Annual in Therapeutic Recreation, 20, 46–58.

[bjhp70027-bib-0071] Thompson, D. M. , Booth, L. , Moore, D. , & Mathers, J. (2022). Peer support for people with chronic conditions: A systematic review of reviews. BMC Health Services Research, 22(1), 427. 10.1186/s12913-022-07816-7 35361215 PMC8973527

[bjhp70027-bib-0072] Vanderhaegen, J. , Raymaekers, K. , Prikken, S. , Claes, L. , Van Laere, E. , Campens, S. , Moons, P. , & Luyckx, K. (2024). Personal and illness identity in youth with type 1 diabetes: Developmental trajectories and associations. Health Psychology, 43(5), 328–338. 10.1037/hea0001366 38252095

[bjhp70027-bib-0073] Walker, G. , & Venker Weidenbenner, J. (2019). Social and emotional learning in the age of virtual play: Technology, empathy, and learning. Journal of Research in Innovative Teaching & Learning, 12(2), 116–132. 10.1108/JRIT-03-2019-0046

[bjhp70027-bib-0074] Warner, R. P. , Sibthorp, J. , Wilson, C. , Browne, L. P. , Barnett, S. , Gillard, A. , & Sorenson, J. (2021). Similarities and differences in summer camps: A mixed methods study of lasting outcomes and program elements. Children and Youth Services Review, 120, 105779. 10.1016/j.childyouth.2020.105779

[bjhp70027-bib-0075] Wheeler, M. , David, A. L. , Kennedy, J. , & Knight, M. (2022). “I sort of never felt like I should be worried about it or that I could be worried about it” an interpretative phenomenological analysis of perceived barriers to disclosure by young people with coeliac disease. British Journal of Health Psychology, 27(4), 1296–1313. 10.1111/bjhp.12599 35574996 PMC9790695

