# Supplementary material for: How to make gluten‐free friends: A quasi‐experimental study on the psychosocial benefits of celiac camps
Source: Br J Health Psychol. 2025 Oct 14;30(4):e70027. doi: 10.1111/bjhp.70027 (PMC12521827; doi:10.1111/bjhp.70027)
Supplement: Supplementary file 1 — Data S1. [file BJHP-30-0-s001.docx]

**How to Make Gluten-Free Friends: A Quasi-Experimental Study on the Psychosocial Benefits of Celiac Camps**

Online Supplementary Materials

Contents

[Extended Information 2](#_Toc179817205)

[IG Zöliakie Camps 2](#_Toc179817206)

[Extended explanations on the analytical strategy 2](#_Toc179817207)

[References 6](#_Toc179817208)

[Quasi-experiment reporting standards 7](#_Toc179817209)

[Preliminary Analysis Tables 14](#_Toc179817210)

[Robustness analysis 40](#_Toc179817211)

[Evaluation: feasibility and acceptability of IG Zöliakie Camps 62](#_Toc179817212)

[Future activities preferences among all study participants 62](#_Toc179817213)

[Camp evaluation: Expectations before the camp 65](#_Toc179817215)

[Friendship formation during the camp: 70](#_Toc179817216)

[Potential for long-term friendships: 71](#_Toc179817217)

[The camp facilitator as a role model: 72](#_Toc179817218)

[Self-assessment of camp’s impact on coping: 73](#_Toc179817219)

[Answers to open questions: 75](#_Toc179817220)

# Extended Information

## IG Zöliakie Camps

we evaluated the potential positive effects of participating in celiac camps organized by IG Zöliakie, the primary celiac support organization in German-speaking Switzerland (<https://www.zoeliakie.ch/de/betroffene/zoelikids/lager.html>) . For over four decades, IG Zöliakie has conducted three annual week-long residential camps for youth aged 8 to 16, each accommodating 20 to 40 participants, including some from neighboring German-speaking countries. The IG Zöliakie camps are meticulously designed to offer a comprehensive experience combining traditional camp activities with celiac-specific educational components (Guastello et al., 2024). Campers engage in various recreational activities such as sports, social games, excursions, and adventurous programs, fostering peer bonding and skill mastery. Importantly, the camps include celiac-specific activities like cooking and baking gluten-free meals together, visiting gluten-free bakeries, and sharing meals without concern for dietary restrictions—experiences that are rare in their usual social environments. Trained volunteer counselors, many of whom have CD themselves, serve as relatable role models, enriching the camp experience. By fostering an environment where dietary management is normalized and shared, the camps aim to reduce feelings of isolation and promote autonomy and self-management skills.

## Extended explanations on the analytical strategy

All analyses were conducted using R version 4.4.1 (R Core Team, 2024). Due to the small number of participants in each intervention group by camp attended or matched to, and to increase statistical power, data were collapsed across the three camps. This approach allowed us to treat specific camps as a random factor and focus on overall camping effects.

Missing data on dependent variables were analyzed separately for children (ages 8–12 years) and adolescents (ages 13-16 years), considering the more extensive measures used in the older cohort. Among children aged 8-12 years, missing data accounted for 8.2% of the dependent variables, primarily due to attrition at posttest. For adolescents aged 13-16 years, missing data accounted for 6.5% of all values. In total, 11 participants (7 in the intervention group and 4 in the control group) did not complete the post-camp measures. To assess potential discrepancies between those who completed and those who did not complete the post-camp measures, we conducted Little's test for missing completely at random (MCAR) (Little & Rubin, 2020). The results indicated that the data were MCAR for both age groups (ages 8–12: χ²(168) = 193.49, *p* = .086; ages 13–16: χ²(187) = 216.50, *p* = .069). Given the small number of attrition cases, we did not conduct further dropout analyses to examine the risk of differential attrition.

We performed a logistic regression analysis examining the equivalence of a comparison group to an intervention group in demographics, illness-related variables, and outcome variables that were measured in both age groups. To examine the equivalence between the comparison group and the intervention group on outcomes measured separately for children and adolescents, a follow-up series of non-parametric Mann-Whitney U tests were conducted. This test is appropriate for comparing two independent groups, particularly when the data does not follow a normal distribution and considering that the sample size within each age group is fairly small compared to the whole data together.

To assess comparability between the two intervention groups, a logistic regression was utilized, predicting condition (1 = campers) from demographics, illness-related variables, and outcomes that were measured in both age groups at pretest. For outcomes measured separately for children and adolescents, we conducted a series of complementary non-parametric Mann-Whitney U tests. This test is appropriate for comparing two independent groups, particularly when the data do not follow a normal distribution and considering the relatively small sample size within each age group.

Testing for multivariate normality revealed several deviations from normal distribution across the outcome variables (see Supplementary Table S3 below). Considering these findings, as well as in order to maximize analytical power by including all available cases, we utilized linear mixed models (LMMs) for our main analyses using the lme4 package in R (Bates et al., 2024). LMMs are advantageous due to their flexibility and robustness in handling repeated measures data, ability to account for the correlation between repeated measures, and capacity to handle missing data without requiring complete cases. This approach provides unbiased estimates under the assumption that data are missing at random (MAR) (Raudenbush & Bryk, 2010; Singer & Willett, 2003). By using LMMs, we included all participants in the analyses, including those who were lost to follow-up.

Given the differences in prior camp attendance between the two intervention groups, a dichotomous variable representing prior participation in celiac camps (experienced vs. novice) was included as an additional fixed factor in the linear mixed models (LMMs), along with condition (intervention vs. control) and time of testing (pretest vs. posttest). This allowed us to examine differential effects between first-time and experienced campers, create more equivalent groups, and explore three-way interactions among prior camp experience, intervention condition, and time to understand how prior experience moderates the intervention's impact. Our primary focus was on the interaction between condition and time, as well as the three-way interaction among prior camp experience, condition, and time. Furthermore, a random intercept accounts for repeated measures within individuals. Degrees of freedom for significance testing were approximated using the Kenward-Roger method (Kenward & Roger, 1997) as implemented in the sjPlot package (Lüdecke, 2024). Estimated marginal means and standard errors for continuous outcomes were obtained using the emmeans package (Lenth, 2024), and planned comparisons were performed using the Tukey method with Kenward-Roger approximation for degrees of freedom. When interactions were marginally significant (*p* < .10), exploratory planned comparisons were conducted but reported only for pre-post comparisons within groups (combinations of age, prior camping experience, and condition) that included at least 10 participants to ensure validity (see Table S8 below).

A post-hoc power analysis indicated that with our sample size (N = 100), we had 82.9% power to detect large effects (*d* = 0.60) in mean differences between groups and 98.1% power to detect significant interaction effects of medium magnitude (η² = .04).

Finally, for robustness, we conducted two follow-up analyses. First, we repeated the LMMs including covariates as control variables: gender (1 = female), age, age of diagnosis, socioeconomic status (SES), and the presence of additional health conditions (dichotomous). Second, we conducted equivalent nonparametric tests for the pre-post change within each group using Wilcoxon signed-rank test. We report on differences between the controlled and uncontrolled analyses, as well as between the parametric and nonparametric approaches, to assess the stability of our findings.

Statistical significance was determined by two-sided tests with a threshold of *p* < .05. Given the relatively small sample size and complexity of the main analyses, we also noted test statistics with *p*-values less than .10. These near-significant results were followed up with exploratory pairwise comparisons, as they may indicate notable trends worthy of further examination in future research (Perneger, 1998).

## References

Bates, D., Maechler, M., Bolker, B., & Walker, S. (2024). *lme4: Linear Mixed-Effects Models using “Eigen” and S4* (p. 1.1-35.5) [Dataset]. https://doi.org/10.32614/CRAN.package.lme4

Guastello, A. D., Barthle-Herrera, M. A., Downing, S., Mirhosseini, T., Valko, A., & McNamara, J. P. H. (2024). Using Summer Camps as Opportunities to Provide Brief Interventions. In T. E. Davis Iii & E. A. Storch (Eds.), *Brief CBT and Science-Based Tailoring for Children, Adolescents, and Young Adults* (pp. 135–150). Springer Nature Switzerland. https://doi.org/10.1007/978-3-031-60746-2_8

Kenward, M. G., & Roger, J. H. (1997). Small Sample Inference for Fixed Effects from Restricted Maximum Likelihood. *Biometrics*, *53*(3), 983. https://doi.org/10.2307/2533558

Lenth, R. V. (2024). *emmeans: Estimated Marginal Means, aka Least-Squares Means* (p. 1.10.4) [Dataset]. https://doi.org/10.32614/CRAN.package.emmeans

Little, R. J. A., & Rubin, D. B. (2020). *Statistical analysis with missing data* (3rd edition). Wiley.

Lüdecke, D. (2024). *sjPlot: Data Visualization for Statistics in Social Science* (p. 2.8.16) [Dataset]. https://doi.org/10.32614/CRAN.package.sjPlot

Perneger, T. V. (1998). What’s wrong with Bonferroni adjustments. *BMJ*, *316*(7139), 1236–1238. https://doi.org/10.1136/bmj.316.7139.1236

Raudenbush, S. W., & Bryk, A. S. (2010). *Hierarchical linear models: Applications and data analysis methods* (2. ed., [Nachdr.]). Sage Publ.

Singer, J. D., & Willett, J. B. (2003). *Applied Longitudinal Data Analysis: Modeling Change and Event Occurrence* (1st ed.). Oxford University PressNew York. https://doi.org/10.1093/acprof:oso/9780195152968.001.0001

# Quasi-experiment reporting standards

**Table S1**


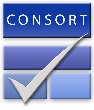
CONSORT 2010 checklist of information to include when reporting a randomised trial* (adapted for quasi-experimental, waitlist-matching design)

| Section/Topic | Item No | Checklist item | Reported on page No |
| --- | --- | --- | --- |
| Title and abstract | | | |
|  | 1a | Identification as a randomised trial in the title | not applicable (non-randomized trial), instead “quasi-experimental study” in the title |
|  | 1b | Structured summary of trial design, methods, results, and conclusions (for specific guidance see CONSORT for abstracts) | Abstract (p. 2) |
| Introduction | | | |
| Background and objectives | 2a | Scientific background and explanation of rationale | Introduction section, p. 3-4 |
|  | 2b | Specific objectives or hypotheses | The Present Study, p. 4-8 |
| Methods | | | |
| Trial design | 3a | Description of trial design (such as parallel, factorial) including allocation ratio | Method secdiont, p. 8-9, Figure 2 |
|  | 3b | Important changes to methods after trial commencement (such as eligibility criteria), with reasons | P. 9 (Sample characteristics), p. 13 (preliminary analysis) |
| Participants | 4a | Eligibility criteria for participants | p. 8 (Participants and procedure) |
|  | 4b | Settings and locations where the data were collected | p. 8-9 (Participants and procedure) |
| Interventions | 5 | The interventions for each group with sufficient details to allow replication, including how and when they were actually administered | Figure 1 (logic model), section: “Extended information on IG Zöliakie Camps”, Supplementary Materials |
| Outcomes | 6a | Completely defined pre-specified primary and secondary outcome measures, including how and when they were assessed | Methods/Measures section (p. 9-12) |
|  | 6b | Any changes to trial outcomes after the trial commenced, with reasons | Not applicable |
| Sample size | 7a | How sample size was determined | Participants and Procedure (p. 8-9), Data analysis (post-hoc power, p. 13) |
|  | 7b | When applicable, explanation of any interim analyses and stopping guidelines | Not applicable |
| Randomisation: |  |  |  |
| Sequence generation | 8a | Method used to generate the random allocation sequence | not applicable (non-randomized trial) |
|  | 8b | Type of randomisation; details of any restriction (such as blocking and block size) | not applicable (non-randomized trial) |
| Allocation concealment mechanism | 9 | Mechanism used to implement the random allocation sequence (such as sequentially numbered containers), describing any steps taken to conceal the sequence until interventions were assigned | not applicable (non-randomized trial) |
| Implementation | 10 | Who generated the random allocation sequence, who enrolled participants, and who assigned participants to interventions | not applicable (non-randomized trial) |
| Blinding | 11a | If done, who was blinded after assignment to interventions (for example, participants, care providers, those assessing outcomes) and how | not applicable (non-randomized trial) |
|  | 11b | If relevant, description of the similarity of interventions | not applicable, only one intervention |
| Statistical methods | 12a | Statistical methods used to compare groups for primary and secondary outcomes | Data analysis (p. 12-13), Results (p. 13-15) |
|  | 12b | Methods for additional analyses, such as subgroup analyses and adjusted analyses | Robustness analysis: Data analysis (p. 12-13), Results (p. 15) |
| Results | | | |
| Participant flow (a diagram is strongly recommended) | 13a | For each group, the numbers of participants who were randomly assigned, received intended treatment, and were analysed for the primary outcome | Nonrandomized trial, Figure 2, Participants and Procedure (p. 8-9) |
|  | 13b | For each group, losses and exclusions after randomisation, together with reasons | Figure 2 |
| Recruitment | 14a | Dates defining the periods of recruitment and follow-up | Participants and procedure (p. 8-10) |
|  | 14b | Why the trial ended or was stopped | Not applicable |
| Baseline data | 15 | A table showing baseline demographic and clinical characteristics for each group | Table 1 |
| Numbers analysed | 16 | For each group, number of participants (denominator) included in each analysis and whether the analysis was by original assigned groups | Tables 2-3 |
| Outcomes and estimation | 17a | For each primary and secondary outcome, results for each group, and the estimated effect size and its precision (such as 95% confidence interval) | Tables 2-3, Figure 3, Supplementary Tables S9.1-S9.17 |
|  | 17b | For binary outcomes, presentation of both absolute and relative effect sizes is recommended | Not applicable |
| Ancillary analyses | **18** | **Results of any other analyses performed, including subgroup analyses and adjusted analyses, distinguishing pre-specified from exploratory** | Robustness analysis (p. 15), Supplementary Tables (Tables S10-S13.17) |
| Harms | 19 | All important harms or unintended effects in each group (for specific guidance see CONSORT for harms) | Not applicable |
| Discussion | | | |
| Limitations | 20 | Trial limitations, addressing sources of potential bias, imprecision, and, if relevant, multiplicity of analyses | Discussion, Limitations (p. 18-19) |
| Generalisability | 21 | Generalisability (external validity, applicability) of the trial findings | Discussion (p. 18) |
| Interpretation | 22 | Interpretation consistent with results, balancing benefits and harms, and considering other relevant evidence | Discussion (p. 15-20) |
| Other information | | |  |
| Registration | 23 | Registration number and name of trial registry | Not applicable |
| Protocol | 24 | Where the full trial protocol can be accessed, if available | Not applicable |
| Funding | 25 | Sources of funding and other support (such as supply of drugs), role of funders | Not applicable |

Citation: Schulz KF, Altman DG, Moher D, for the CONSORT Group. CONSORT 2010 Statement: updated guidelines for reporting parallel group randomised trials. BMC Medicine. 2010;8:18.
© 2010 Schulz et al. This is an Open Access article distributed under the terms of the Creative Commons Attribution License (<http://creativecommons.org/licenses/by/2.0>), which permits unrestricted use, distribution, and reproduction in any medium, provided the original work is properly cited.

*We strongly recommend reading this statement in conjunction with the CONSORT 2010 Explanation and Elaboration for important clarifications on all the items. If relevant, we also recommend reading CONSORT extensions for cluster randomised trials, non-inferiority and equivalence trials, non-pharmacological treatments, herbal interventions, and pragmatic trials. Additional extensions are forthcoming: for those and for up-to-date references relevant to this checklist, see [www.consort-statement.org](http://www.consort-statement.org).

**Table S2**

The TREND Checklist for Non-Randomized Trials

| **Paper Section/Topic** | **Item No.** | | **Descriptor** | Reported? | |
| --- | --- | --- | --- | --- | --- |
|  |  |  |  |  | Pg # |
| **TITLE and ABSTRACT** | | | |  |  |
| Title and Abstract | 1 | | - Information on how units were allocated to interventions | X | 8-9, Figure 2 |
|  |  | | - Structured abstract recommended | X | 2 |
|  |  | | - Information on target population or study sample | X | 8-9, Figures 1-2 |
| **INTRODUCTION** | | | |  |  |
| Background | 2 | - Scientific background and explanation of rationale | | X | 3-8 |
|  |  | - Theories used in designing behavioral interventions | | X | 4-8, Figure 1 |
| **METHODS** | | | |  |  |
| Participants | 3 | | - Eligibility criteria for participants, including criteria at different levels in recruitment/sampling plan (e.g., cities, clinics, subjects) | X | 8-9, Figure 2 |
|  |  | | - Method of recruitment (e.g., referral, self-selection), including the sampling method if a systematic sampling plan was implemented | X | 8-9 |
|  |  | | - Recruitment setting | X | 8-9 |
|  |  | | - Settings and locations where the data were collected | X | 8-9 |
| Interventions | 4 | | - Details of the interventions intended for each study condition and how and when they were actually administered, specifically including: | X | 4-5, Figure 1, supplementary materials |
|  |  | | - - Content: what was given? | X | 4-5, Figure 1, supplementary materials |
|  |  | | - - Delivery method: how was the content given? | X | 4-5, Figure 1, supplementary materials |
|  |  | | - - Unit of delivery: how were subjects grouped during delivery? | X | 4-5, Figure 1, supplementary materials |
|  |  | | - - Deliverer: who delivered the intervention? | X | 4-5, Figure 1, supplementary materials |
|  |  | | - - Setting: where was the intervention delivered? | X | 4-5, Figure 1, supplementary materials |
|  |  | | - - Exposure quantity and duration: how many sessions or episodes or events were intended to be delivered? How long were they intended to last? | X | 4-5, Figure 1, supplementary materials |
|  |  | | - - Time span: how long was it intended to take to deliver the intervention to each unit? | X | 4-5, Figure 1, supplementary materials |
|  |  | | - - Activities to increase compliance or adherence (e.g., incentives) |  | N/A |
| Objectives | 5 | | - Specific objectives and hypotheses | X | 4-8 |
| Outcomes | 6 | | - Clearly defined primary and secondary outcome measures | X | 9-12 |
|  |  | | - Methods used to collect data and any methods used to enhance the quality of measurements | X | 8-12 |
|  |  | | - Information on validated instruments such as psychometric and biometric properties | X | 9-12, supplementary materials |
| Sample size | 7 | | - How sample size was determined and, when applicable, explanation of any interim analyses and stopping rules | X | 8-9, Figure 2 |
| Assignment method | 8 | | - Unit of assignment (the unit being assigned to study condition, e.g., individual, group, community) | X | 8-9, Figure 2 |
|  |  | | - Method used to assign units to study conditions, including details of any restriction (e.g., blocking, stratification, minimization) | X | 8-9, Figure 2 |
|  |  | | - Inclusion of aspects employed to help minimize potential bias induced due to non-randomization (e.g., matching) | X | 8-9, Figure 2 |
| Blinding (masking) | 9 | | - Whether or not participants, those administering the interventions, and those assessing the outcomes were blinded to study condition assignment; if so, statement regarding how the blinding was accomplished and how it was assessed |  | N/A |
| Unit of Analysis | 10 | | - Description of the smallest unit that is being analysed to assess intervention effects (e.g., individual, group, or community) | X | 12-13 |
|  |  | | - If the unit of analysis differs from the unit of assignment, the analytical method used to account for this (e.g., adjusting the standard error estimates by the design effect or using multilevel analysis) |  | N/A |
| Statistical methods | 11 | | - Statistical methods used to compare study groups for primary methods outcome(s), including complex methods for correlated data | X | 12-13, supplementary materials |
|  |  |  | - Statistical methods used for additional analyses, such as subgroup analyses and adjusted analysis | X | 12-13, supplementary materials |
|  |  |  | - Methods for imputing missing data, if used |  | N/A |
|  |  |  | - Statistical software or programs used | X | 12 |
| **RESULTS** | | | |  |  |
| Participant flow | 12 | | - Flow of participants through each stage of the study: enrollment, assignment, allocation and intervention exposure, follow-up, analysis (a diagram is strongly recommended) | X | Figure 2 |
|  |  | | - - Enrollment: the numbers of participants screened for eligibility, found to be eligible or not eligible, declined to be enrolled, and enrolled in the study | X | Figure 2 |
|  |  | | - - Assignment: the numbers of participants assigned to a study condition | X | Figure 2 |
|  |  | | - - Allocation and intervention exposure: the number of participants assigned to each study condition and the number of participants who received each intervention | X | Figure 2 |
|  |  | | - - Follow-up: the number of participants who completed the follow-up or did not complete the follow-up (i.e., lost to follow-up), by study condition | X | Figure 2 |
|  |  | | - - Analysis: the number of participants included in or excluded from the main analysis, by study condition | X | Figure 2 |
|  |  | | - Description of protocol deviations from study as planned, along with reasons |  | N/A |
| Recruitment | 13 | | - Dates defining the periods of recruitment and follow-up | X | 12-13 |
| Baseline data | 14 | | - Baseline demographic and clinical characteristics of participants in each study condition | X | 9, Table 1 |
|  |  | | - Baseline characteristics for each study condition relevant to specific disease prevention research | X | 9, Table 1 |
|  |  | | - Baseline comparisons of those lost to follow-up and those retained, overall and by study condition |  | N/A |
|  |  | | - Comparison between study population at baseline and target population of interest |  | N/A |
| Baseline equivalence | 15 | | - Data on study group equivalence at baseline and statistical methods used to control for baseline differences | X | 13, supplementary Tables S4-S5 |
| Numbers analyzed | 16 | | - Number of participants (denominator) included in each analysis for each study condition, particularly when the denominators change for different outcomes; statement of the results in absolute numbers when feasible | X | Tables 2-3, supplementary Tables S8, S91.-S9.17 |
|  |  | | - Indication of whether the analysis strategy was “intention to treat” or, if not, description of how non-compliers were treated in the analyses | X | Figure 2 |
| Outcomes and estimation | 17 | | - For each primary and secondary outcome, a summary of results for each estimation study condition, and the estimated effect size and a confidence interval to indicate the precision | X | 13-15, Tables 2-3, Supplementary Tables S9,1-S9.17 |
|  |  | | - Inclusion of null and negative findings | X | 13-15, Tables 2-3, Supplementary Tables S9,1-S9.17 |
|  |  | | - Inclusion of results from testing pre-specified causal pathways through which the intervention was intended to operate, if any |  | N/A |
| Ancillary analyses | 18 | | - Summary of other analyses performed, including subgroup or restricted analyses, indicating which are pre-specified or exploratory | X | 15, Supplementary Tables S10-S13.17 |
| Adverse events | 19 | | - Summary of all important adverse events or unintended effects in each study condition (including summary measures, effect size estimates, and confidence intervals) |  | N/A |
| **DISCUSSION** | | | |  |  |
| Interpretation | 20 | | - Interpretation of the results, taking into account study hypotheses, sources of potential bias, imprecision of measures, multiplicative analyses, and other limitations or weaknesses of the study | X | 15-18 |
|  |  | | - Discussion of results taking into account the mechanism by which the intervention was intended to work (causal pathways) or alternative mechanisms or explanations | X | 15-18 |
|  |  | | - Discussion of the success of and barriers to implementing the intervention, fidelity of implementation | X | Evaluation report, supplementary materials |
|  |  | | - Discussion of research, programmatic, or policy implications | X | 19-20 |
| Generalizability | 21 | | - Generalizability (external validity) of the trial findings, taking into account the study population, the characteristics of the intervention, length of follow-up, incentives, compliance rates, specific sites/settings involved in the study, and other contextual issues | X | 18 |
| Overall evidence | 22 | | - General interpretation of the results in the context of current evidence and current theory | X | 15-18 |

# Preliminary Analysis Tables

**Table S3**

Descriptive Statistics and Normal distribution

| **Variable** | **Time** | **M** | **SD** | **N** | **Range (Min)** | **Range (Max** | **Skewness** | **Kurtosis** | **Shapiro Wilk Statistic** | **Shapiro Wilk p value** | **Cronbach Alpha** |
| --- | --- | --- | --- | --- | --- | --- | --- | --- | --- | --- | --- |
| CD Friendships | Pre | 2.24 | 0.84 | 111 | 1 | 4 | 0.47 | 2.35 | 0.92 | < .001 | r = .60, p < .001 |
|  | post | 2.64 | 0.96 | 99 | 1 | 4 | -0.09 | 1.75 | 0.92 | < .001 | r = .66, p < .001 |
| Peer support | Pre | 3.16 | 0.89 | 36 | 1 | 5 | -0.29 | 3.13 | 0.96 | 0.155 | 0.53 |
|  | post | 3.13 | 0.88 | 33 | 1.33 | 5 | -0.35 | 2.64 | 0.95 | 0.165 | 0.61 |
| IIQ-Rejection | Pre | 2.17 | 0.93 | 36 | 1 | 4.4 | 0.55 | 2.37 | 0.93 | 0.032 | 0.71 |
|  | post | 1.99 | 0.75 | 33 | 1 | 3.6 | 0.29 | 2.04 | 0.94 | 0.065 | 0.75 |
| IIQ-Acceptance | Pre | 4.24 | 0.78 | 36 | 2.4 | 5 | -1.02 | 3.11 | 0.86 | < .001 | 0.86 |
|  | post | 4.28 | 0.78 | 33 | 2 | 5 | -1.05 | 3.44 | 0.85 | < .001 | 0.83 |
| IIQ-Engulfment | Pre | 1.84 | 0.83 | 36 | 1 | 4.38 | 1.4 | 4.63 | 0.85 | < .001 | 0.89 |
|  | post | 1.74 | 0.71 | 33 | 1 | 3.38 | 1.01 | 3.02 | 0.86 | < .001 | 0.87 |
| IIQ-Enrichment | Pre | 2.68 | 0.95 | 36 | 1 | 5 | 0.34 | 2.75 | 0.97 | 0.351 | 0.88 |
|  | post | 2.83 | 0.88 | 33 | 1 | 4.71 | -0.21 | 2.83 | 0.96 | 0.329 | 0.84 |
| DIFI | Pre | 36.92 | 42.01 | 109 | 0 | 100 | 0.54 | 1.54 | 0.76 | < .001 |  |
|  | post | 37.71 | 42.57 | 98 | 0 | 100 | 0.57 | 1.57 | 0.75 | < .001 |  |
| GFD-PCS | Pre | 4.79 | 0.32 | 110 | 3.75 | 5 | -1.46 | 4.24 | 0.7 | < .001 | 0.69 |
|  | post | 4.76 | 0.41 | 98 | 2.75 | 5 | -2.18 | 8.73 | 0.65 | < .001 | 0.79 |
| CDAT | Pre | 1.65 | 0.45 | 110 | 1 | 3.29 | 0.76 | 3.8 | 0.95 | < .001 | 0.42 |
|  | post | 1.63 | 0.53 | 99 | 1 | 3.29 | 1.08 | 3.73 | 0.9 | < .001 | 0.61 |
| CDPQOL: Ages 8-12 (*n* = 75) | | | | | | | | | | | |
| Negative emotions | Pre | 1.94 | 0.65 | 74 | 1 | 4.14 | 1.15 | 4.7 | 0.92 | < .001 | 0.81 |
|  | post | 1.84 | 0.59 | 66 | 1 | 4 | 1.18 | 5.03 | 0.92 | < .001 | 0.74 |
| School | Pre | 1.64 | 0.63 | 74 | 1 | 4.25 | 1.55 | 6.32 | 0.85 | < .001 | 0.65 |
|  | post | 1.62 | 0.6 | 64 | 1 | 3.75 | 1.29 | 4.35 | 0.85 | < .001 | 0.63 |
| Enjoyment | Pre | 1.32 | 0.54 | 74 | 1 | 3 | 1.72 | 5.16 | 0.66 | < .001 | 0.62 |
|  | post | 1.42 | 0.66 | 65 | 1 | 3 | 1.46 | 3.84 | 0.67 | < .001 | 0.59 |
| CDPQOL: Ages 13-16 (*n* = 36) | | | | | | | | | | | |
| Social | Pre | 2.02 | 0.84 | 36 | 1 | 3.86 | 0.69 | 2.4 | 0.91 | 0.007 | 0.86 |
|  | post | 2.03 | 0.82 | 33 | 1 | 3.86 | 0.83 | 2.96 | 0.91 | 0.009 | 0.88 |
| Uncertainty | Pre | 1.77 | 0.78 | 36 | 1 | 4 | 0.88 | 3.09 | 0.87 | < .001 | 0.47 |
|  | post | 1.97 | 0.78 | 33 | 1 | 3.67 | 0.35 | 2.01 | 0.92 | 0.019 | 0.51 |
| Isolation | Pre | 1.89 | 0.68 | 36 | 1 | 3.5 | 0.51 | 2.41 | 0.94 | 0.057 | 0.67 |
|  | post | 1.81 | 0.57 | 33 | 1 | 3.25 | 0.79 | 2.92 | 0.92 | 0.024 | 0.57 |
| Limitation | Pre | 2.26 | 1.01 | 36 | 1 | 4.33 | 0.45 | 2.03 | 0.92 | 0.015 | 0.7 |
|  | post | 2.23 | 0.98 | 33 | 1 | 4.33 | 0.3 | 2.12 | 0.93 | 0.038 | 0.74 |
| SWB | Pre | 8.91 | 1.66 | 110 | 1 | 11 | -1.91 | 8.18 | 0.8 | < .001 |  |
|  | post | 8.97 | 1.43 | 98 | 2 | 11 | -2 | 9.38 | 0.78 | < .001 |  |

Note. CD = Celiac disease. IIQ = Illness Identity Questionnaire. DIFI = Dynamic Identity Fusion Index. CFD-PCS = Gluten-Free Diet Perceived Competence Scale. CDAT = Celiac Dietary Adherence Test. CDPQOL = Pediatric Celiac Disease Quality of Life (measured with two versions, age-dependent). SWB = Subjective Well-Being. Peer support and IIQ outcomes were measured only for participants aged 13–16 (n = 36).

**Table S4**

*Logistic regression examining comparison group’s equivalence to intervention group in demographics and illness related variables*

|  | **Condition (1 = Intervention/ campers)** | | | | | | |
| --- | --- | --- | --- | --- | --- | --- | --- |
| *Predictors* | *OR* | *SE* | *β* | *Std. SE* | 95% *CI* | *Std. CI* | *p* |
| (Intercept) | 0.000 | 0.001 | 5.043 | 2.896 | 0.000 – 20.116 | 1.775 – 17.300 | 0.151 |
| Prior camping experience | 4.396 | 1.936 | 5.068 | 2.447 | 1.995 – 11.546 | 2.132 – 14.605 | **0.001** |
| Age | 1.107 | 0.175 | 1.239 | 0.413 | 0.810 – 1.516 | 0.640 – 2.408 | 0.521 |
| Age of diagnosis | 1.011 | 0.094 | 1.034 | 0.289 | 0.840 – 1.216 | 0.593 – 1.801 | 0.904 |
| SES | 1.036 | 0.247 | 1.042 | 0.291 | 0.646 – 1.668 | 0.600 – 1.821 | 0.884 |
| Gender (1 = female) | 0.327 | 0.205 | 0.327 | 0.205 | 0.090 – 1.071 | 0.090 – 1.071 | 0.075 |
| CD friendship | 1.183 | 0.571 | 1.149 | 0.460 | 0.453 – 3.076 | 0.519 – 2.537 | 0.728 |
| DIFI - Overlap | 1.013 | 0.016 | 1.686 | 1.082 | 0.982 – 1.045 | 0.480 – 6.115 | 0.416 |
| GFD-PCS | 1.597 | 1.640 | 1.153 | 0.362 | 0.213 – 12.598 | 0.624 – 2.166 | 0.649 |
| CDAT | 1.265 | 1.057 | 1.105 | 0.391 | 0.242 – 6.690 | 0.548 – 2.238 | 0.779 |
| SWB | 1.145 | 0.230 | 1.258 | 0.427 | 0.772 – 1.715 | 0.646 – 2.488 | 0.500 |
| Observations | 98 | | | | | | |
| R^2^ Tjur | 0.374 | | | | | | |
| AIC | 116.279 | | | | | | |
| log-Likelihood | -45.140 | | | | | | |

**Table S5**

*Mann-Whitney tests for equivalence of comparison and intervention groups in outcomes measured separately for the two age groups*

| Variable | *N* | Noncampers | | Campers | | Statistic | *p* value |
| --- | --- | --- | --- | --- | --- | --- | --- |
|  |  | *M* | *SD* | *M* | *SD* |  |  |
| Ages 8-12 (*n* = 75) |  |  |  |  |  |  |  |
| CDPQOL: Negative emotions | 74 | 1.97 | 0.6 | 1.91 | 0.7 | 746 | 0.476 |
| CDPQOL: School | 74 | 1.67 | 0.67 | 1.61 | 0.59 | 706.5 | 0.775 |
| CDPQOL: Enjoyment | 74 | 1.32 | 0.55 | 1.32 | 0.54 | 661.5 | 0.818 |
| Ages 13-16 (*n* = 36) |  |  |  |  |  |  |  |
| Peer support | 36 | 2.91 | 1.08 | 3.27 | 0.8 | 102 | 0.223 |
| IIQ: Rejection | 36 | 2.36 | 0.88 | 2.08 | 0.95 | 166 | 0.334 |
| IIQ: Acceptance | 36 | 3.75 | 0.8 | 4.46 | 0.67 | 60 | 0.008 |
| IIQ: Engulfment | 36 | 2.02 | 1.14 | 1.76 | 0.66 | 145.5 | 0.796 |
| IIQ: Enrichment | 36 | 2.72 | 0.75 | 2.66 | 1.05 | 145.5 | 0.796 |
| CDPQOL: Social | 36 | 2.55 | 0.94 | 1.79 | 0.68 | 202 | 0.028 |
| CDPQOL: Uncertainty | 36 | 1.97 | 0.81 | 1.68 | 0.76 | 170.5 | 0.255 |
| CDPQOL: Isolation | 36 | 2.09 | 0.69 | 1.81 | 0.67 | 170 | 0.268 |
| CDPQOL: Limitation | 36 | 2.82 | 1.08 | 2.01 | 0.88 | 197 | 0.041 |

*Note*. IIQ = Illness Identity Questionnaire. CDPQOL = Pediatric Celiac Disease Quality of Life (measured with two versions, age-dependent).

**Table S6**

Spearman correlation matrix for pretest (below the diagonal) and posttest (above the diagonal)

| Variable | 1 | 2 | 3 | 4 | 5 | 6 | 7 | 8 | 9 | 10 | 11 | 12 | 13 | 14 | 15 | 16 | 17 | 18 |
| --- | --- | --- | --- | --- | --- | --- | --- | --- | --- | --- | --- | --- | --- | --- | --- | --- | --- | --- |
| 1. Prior camping | - | .56*** | -.04 | -.24 | .21 | -.09 | -.12 | .08 | .07 | .21* | .03 | -.14 | .01 | -.13 | .08 | -.2 | -.07 | -.06 |
| 2. CD friendship | .68*** | - | .01 | -.27 | .39* | .00 | .00 | .15 | .13 | .10 | -.10 | -.25* | -.04 | -.11 | .05 | -.04 | -.04 | .00 |
| 3. Peer support | .24 | .30 | - | -.6*** | .62*** | -.48** | .01 | .18 | .38* | -.32 |  |  | -.78*** | -.72*** | -.65*** | -.87*** | .41* |  |
| 4. IIQ: Rejection | -.24 | -.39* | -.39* | - | -.62*** | .47** | -.04 | -.23 | -.10 | .35* |  |  | .58*** | .42* | .51** | .61*** | -.19 |  |
| 5. IIQ: Acceptance | .26 | .28 | .52** | -.58*** | - | -.45** | .30 | .24 | .54** | -.47** |  |  | -.72*** | -.45** | -.49** | -.7*** | .53** |  |
| 6. IIQ: Engulfment | -.21 | -.09 | -.4* | .27 | -.37* | - | .04 | -.01 | -.35* | -.08 |  |  | .58*** | .67*** | .44** | .59*** | -.18 |  |
| 7. IIQ: Enrichment | .05 | .03 | .09 | -.18 | .21 | .24 | - | -.02 | .16 | -.07 |  |  | -.09 | .12 | .02 | -.04 | .4* |  |
| 8. DIFI - Overlap | .26** | .19 | .32 | -.20 | .37* | -.27 | .24 | - | .02 | -.02 | -.04 | .08 | -.14 | -.08 | .15 | -.19 | -.03 | .24* |
| 9. GFD-PCS | .07 | .06 | .47** | -.32 | .56*** | -.42* | .14 | .31** | - | -.33** | -.36** | -.29* | -.39** | -.47** | -.5** | -.30 | -.53** | .37*** |
| 10. CDAT | .15 | .08 | -.23 | .20 | -.30 | -.04 | -.30 | -.14 | -.33** | - | .33** | .29* | .31* | .32 | .20 | .33 | .37* | -.4*** |
| 11. CDPQOL: Negative emotions | .02 | -.04 |  |  |  |  |  |  |  |  | - | -.47*** | .42*** |  |  |  | -.2 |  |
| 12. CDPQOL: School | .06 | .06 |  |  |  |  |  |  |  |  | -.05 | - | -.28* | .33** | .57*** |  | -.4** |  |
| 13. CDPQOL: Enjoyment | .07 | -.07 |  |  |  |  |  |  |  |  | -.14 | -.28* | - | .38*** | .55*** | .56*** |  | -.43*** |
| 14. CDPQOL: Social | -.28 | -.27 | -.7*** | .45** | -.66*** | .45** | -.18 | -.27 | -.6*** | .37* |  |  | - | .71*** | .63*** | .89*** | -.49** |  |
| 15. CDPQOL: Uncertainty | -.09 | -.16 | -.44** | .43** | -.54*** | .66*** | .05 | -.10 | -.46** | .32 |  |  | .71*** | - | .51** | .77*** | -.24 |  |
| 16. CDPQOL: Isolation | -.15 | -.10 | -.62*** | .47** | -.69*** | .49** | -.12 | -.28 | -.57*** | .21 |  |  | .75*** | .61*** | - | .69*** | -.18 |  |
| 17. CDPQOL: Limitation | -.26 | -.30 | -.71*** | .50** | -.65*** | .42** | -.16 | -.20 | -.50** | .28 |  |  | .89*** | .66*** | .81*** | - | -.41* |  |
| 18. SWB | -.06 | -.05 | .43** | -.12 | .52** | -.01 | .5** | .13 | .39*** | -.52*** | -.6*** | -.44*** | -.47*** | -.48** | -.26 | -.42* | -.51** | - |

Note. CD = Celiac disease. IIQ = Illness Identity Questionnaire. DIFI = Dynamic Identity Fusion Index. CFD-PCS = Gluten-Free Diet Perceived Competence Scale. CDAT = Celiac Dietary Adherence Test. CDPQOL = Pediatric Celiac Disease Quality of Life (measured with two versions, age-dependent). SWB = Subjective Well-Being. Peer support and IIQ outcomes were measured only for participants aged 13–16 (n = 36).

* *p* < .05, ** *p* < .01, *** *p* < .001.

**Table S7.1**

*Spearman correlations between demographics, illness related and outcome variables at pretest:*

| **Variable** | **Age** | **Age of diagnosis** | **SES** | **Prior camping** | **Health satisfaction** |
| --- | --- | --- | --- | --- | --- |
| CD friendship | 0.5*** | -0.09 | -0.1 | 0.68*** | -0.04 |
| Peer support | 0.08 | -0.04 | 0.09 | 0.24 | 0.21 |
| IIQ: Rejection | -0.1 | -0.03 | 0.05 | -0.24 | 0.06 |
| IIQ: Acceptance | 0.01 | -0.07 | 0.16 | 0.26 | 0.28 |
| IIQ: Engulfment | 0.24 | 0 | -0.1 | -0.21 | -0.27 |
| IIQ: Enrichment | 0.42* | 0.13 | 0.33 | 0.05 | 0.15 |
| DIFI - Overlap | 0 | -0.19 | 0.04 | 0.26** | 0.19* |
| GFD-PCS | -0.05 | -0.16 | -0.02 | 0.07 | 0.17 |
| CDAT | 0.29** | 0.16 | -0.15 | 0.15 | -0.16 |
| CDPQOL: Negative emotions | 0.04 | -0.03 | 0 | 0.02 | -0.26* |
| CDPQOL: School | -0.03 | 0.19 | 0.1 | 0.06 | -0.09 |
| CDPQOL: Enjoyment | -0.1 | 0.17 | 0.03 | 0.07 | -0.25* |
| CDPQOL: Social | -0.15 | 0.17 | 0.01 | -0.28 | -0.38* |
| CDPQOL: Uncertainty | 0.01 | 0.11 | 0.03 | -0.09 | -0.2 |
| CDPQOL: Isolation | 0.02 | -0.09 | 0.07 | -0.15 | -0.19 |
| CDPQOL: Limitation | -0.1 | -0.04 | 0.02 | -0.26 | -0.31 |
| SWB | -0.25** | -0.08 | 0.14 | -0.06 | 0.22* |

Note. CD = Celiac disease. IIQ = Illness Identity Questionnaire. DIFI = Dynamic Identity Fusion Index. CFD-PCS = Gluten-Free Diet Perceived Competence Scale. CDAT = Celiac Dietary Adherence Test. CDPQOL = Pediatric Celiac Disease Quality of Life (measured with two versions, age-dependent, 8-12: n = 75, 13-16: n = 36). SWB = Subjective Well-Being. Peer support and IIQ outcomes were measured only for participants aged 13–16 (n = 36).

* *p* < .05, ** *p* < .01, *** *p* < .001.

**Table S7.2**

*Spearman correlations between demographics, illness related and outcome variables at posttest:*

| **Variable** | **Age** | **Age of diagnosis** | **SES** | **Prior camping** | **Health satisfaction** |
| --- | --- | --- | --- | --- | --- |
| CD friendship | 0.46*** | 0.01 | -0.13 | 0.56*** | 0.1 |
| Peer support | -0.09 | 0.24 | -0.07 | -0.04 | 0.19 |
| IIQ: Rejection | -0.08 | -0.22 | 0.14 | -0.24 | -0.16 |
| IIQ: Acceptance | -0.03 | 0.07 | -0.05 | 0.21 | 0.22 |
| IIQ: Engulfment | 0.21 | 0.09 | 0.02 | -0.09 | -0.07 |
| IIQ: Enrichment | -0.21 | 0.3 | 0.44* | -0.12 | 0.06 |
| DIFI - Overlap | -0.13 | -0.04 | -0.04 | 0.08 | 0.07 |
| GFD-PCS | -0.06 | -0.03 | 0.04 | 0.07 | 0.03 |
| CDAT | 0.37*** | 0.1 | -0.1 | 0.21* | -0.07 |
| CDPQOL: Negative emotions | 0.15 | 0.13 | 0.06 | 0.03 | -0.14 |
| CDPQOL: School | 0 | 0.11 | 0.06 | -0.14 | -0.15 |
| CDPQOL: Enjoyment | 0.02 | 0.05 | 0.16 | 0.01 | -0.02 |
| CDPQOL: Social | -0.07 | -0.1 | 0.05 | -0.13 | -0.25 |
| CDPQOL: Uncertainty | 0.06 | 0.01 | 0.15 | 0.08 | -0.36* |
| CDPQOL: Isolation | -0.03 | -0.08 | 0.07 | -0.2 | 0.19 |
| CDPQOL: Limitation | -0.04 | -0.18 | 0.09 | -0.07 | -0.29 |
| SWB | -0.19 | -0.06 | 0.11 | -0.06 | 0.11 |

Note. CD = Celiac disease. IIQ = Illness Identity Questionnaire. DIFI = Dynamic Identity Fusion Index. CFD-PCS = Gluten-Free Diet Perceived Competence Scale. CDAT = Celiac Dietary Adherence Test. CDPQOL = Pediatric Celiac Disease Quality of Life (measured with two versions, age-dependent, 8-12: n = 75, 13-16: n = 36). SWB = Subjective Well-Being. Peer support and IIQ outcomes were measured only for participants aged 13–16 (n = 36).

* *p* < .05, ** *p* < .01, *** *p* < .001.

**Table S8**

*Number of participants by age group, prior camp experience group, and condition*

| Age groups | Prior experience | Campers | Noncampers | Total |
| --- | --- | --- | --- | --- |
| Children (aged 8-12 years) | Novice | 19 | 30 | 49 |
|  | Experienced | 22 | 4 | 26 |
|  | Total | 41 | 34 | 75 |
| Adolescents (aged 13-16 years) | Novice | 4 | 7 | 11 |
|  | Experienced | 21 | 4 | 25 |
|  | Total | 25 | 11 | 36 |

*Note*. Cells with low numbers of participants are highlighted in red.

Linear Mixed-Models

**Table S9.1**

*Results of Linear Mixed Model Predicting CD Friendships from Prior Camping, Condition, and Time*

|  | **CD Friendship** | | | | | | | |
| --- | --- | --- | --- | --- | --- | --- | --- | --- |
| *Predictors* | *B* | *SE* | *β* | *Std. SE* | *CI* | *Std. CI* | *p* | *df* |
| (Intercept) | 1.845 | 0.117 | -0.635 | 0.127 | 1.614 – 2.076 | -0.887 – -0.384 | **<0.001** | 168.481 |
| Time | -0.007 | 0.114 | -0.008 | 0.124 | -0.233 – 0.218 | -0.253 – 0.238 | 0.950 | 98.558 |
| Condition | 1.121 | 0.194 | 1.220 | 0.211 | 0.738 – 1.504 | 0.803 – 1.637 | **<0.001** | 174.551 |
| Prior Camping | 0.179 | 0.282 | 0.195 | 0.307 | -0.377 – 0.736 | -0.411 – 0.801 | 0.526 | 172.203 |
| Time X Condition | -1.263 | 0.188 | -1.375 | 0.205 | -1.637 – -0.889 | -1.782 – -0.968 | **<0.001** | 100.855 |
| Time X Prior Camping | 0.108 | 0.274 | 0.118 | 0.298 | -0.435 – 0.651 | -0.474 – 0.709 | 0.694 | 99.949 |
| Condition X Prior Camping | 0.116 | 0.340 | 0.127 | 0.370 | -0.554 – 0.787 | -0.603 – 0.856 | 0.732 | 173.154 |
| Time X Condition X Prior Camping | 0.796 | 0.330 | 0.866 | 0.359 | 0.142 – 1.451 | 0.154 – 1.579 | **0.018** | 100.313 |
| **Random Effects** | | | | | | | | |
| σ^2^ | 0.22 | | | | | | | |
| τ_00_ _ID_Matching_ | 0.25 | | | | | | | |
| ICC | 0.53 | | | | | | | |
| N _ID_Matching_ | 111 | | | | | | | |
| Observations | 210 | | | | | | | |
| Marginal R^2^ / Conditional R^2^ | 0.447 / 0.741 | | | | | | | |

**Table S9.2**

*Results of Linear Mixed Model Predicting Peer Support from Prior Camping, Condition, and Time*

|  | **Peer support** | | | | | | | |
| --- | --- | --- | --- | --- | --- | --- | --- | --- |
| *Predictors* | *B* | *SE* | *β* | *Std. SE* | *CI* | *Std. CI* | *p* | *df* |
| (Intercept) | 3.167 | 0.298 | 0.025 | 0.338 | 2.565 – 3.768 | -0.658 – 0.707 | **<0.001** | 40.845 |
| Time | 0.042 | 0.224 | 0.047 | 0.254 | -0.416 – 0.499 | -0.472 – 0.567 | 0.854 | 28.874 |
| Condition | -0.000 | 0.516 | -0.000 | 0.585 | -1.042 – 1.042 | -1.182 – 1.182 | 1.000 | 40.845 |
| Prior Camping | -1.132 | 0.541 | -1.285 | 0.614 | -2.221 – -0.043 | -2.521 – -0.049 | **0.042** | 46.169 |
| Time X Condition | -0.458 | 0.372 | -0.520 | 0.423 | -1.221 – 0.304 | -1.386 – 0.345 | 0.229 | 28.356 |
| Time X Prior Camping | 0.340 | 0.407 | 0.386 | 0.462 | -0.491 – 1.171 | -0.557 – 1.330 | 0.409 | 29.469 |
| Condition X Prior Camping | 1.221 | 0.711 | 1.386 | 0.808 | -0.213 – 2.654 | -0.242 – 3.013 | 0.093 | 44.195 |
| Time X Condition X Prior Camping | 0.186 | 0.523 | 0.211 | 0.594 | -0.884 – 1.256 | -1.003 – 1.425 | 0.724 | 28.972 |
| **Random Effects** | | | | | | | | |
| σ^2^ | 0.18 | | | | | | | |
| τ_00_ _ID_Matching_ | 0.53 | | | | | | | |
| ICC | 0.75 | | | | | | | |
| N _ID_Matching_ | 37 | | | | | | | |
| Observations | 69 | | | | | | | |
| Marginal R^2^ / Conditional R^2^ | 0.138 / 0.784 | | | | | | | |

**Table S9.3**

*Results of Linear Mixed Model Predicting IIQ: Acceptance from Prior Camping, Condition, and Time*

|  | **IIQ: Acceptance** | | | | | | | |
| --- | --- | --- | --- | --- | --- | --- | --- | --- |
| *Predictors* | *B* | *SE* | *β* | *Std. SE* | *CI* | *Std. CI* | *p* | *df* |
| (Intercept) | 4.050 | 0.271 | -0.272 | 0.350 | 3.503 – 4.597 | -0.979 – 0.435 | **<0.001** | 39.920 |
| Time | -0.348 | 0.192 | -0.450 | 0.248 | -0.741 – 0.045 | -0.958 – 0.059 | 0.081 | 28.780 |
| Condition | 0.450 | 0.469 | 0.582 | 0.606 | -0.497 – 1.397 | -0.643 – 1.807 | 0.343 | 39.920 |
| Prior Camping | -0.256 | 0.489 | -0.331 | 0.633 | -1.241 – 0.730 | -1.606 – 0.944 | 0.604 | 44.912 |
| Time X Condition | 0.248 | 0.320 | 0.320 | 0.414 | -0.407 – 0.902 | -0.526 – 1.167 | 0.445 | 28.314 |
| Time X Prior Camping | 0.403 | 0.349 | 0.522 | 0.452 | -0.311 – 1.118 | -0.402 – 1.446 | 0.258 | 29.312 |
| Condition X Prior Camping | 0.130 | 0.645 | 0.168 | 0.834 | -1.170 – 1.430 | -1.513 – 1.850 | 0.841 | 43.044 |
| Time X Condition X Prior Camping | -0.209 | 0.449 | -0.270 | 0.581 | -1.127 – 0.710 | -1.458 – 0.919 | 0.646 | 28.867 |
| **Random Effects** | | | | | | | | |
| σ^2^ | 0.13 | | | | | | | |
| τ_00_ _ID_Matching_ | 0.45 | | | | | | | |
| ICC | 0.78 | | | | | | | |
| N _ID_Matching_ | 37 | | | | | | | |
| Observations | 69 | | | | | | | |
| Marginal R^2^ / Conditional R^2^ | 0.118 / 0.803 | | | | | | | |

**Table S9.4**

*Results of Linear Mixed Model Predicting IIQ: Rejection from Prior Camping, Condition, and Time*

|  | **IIQ: Rejection** | | | | | | | |
| --- | --- | --- | --- | --- | --- | --- | --- | --- |
| *Predictors* | *B* | *SE* | *β* | *Std. SE* | *CI* | *Std. CI* | *p* | *df* |
| (Intercept) | 2.050 | 0.270 | -0.037 | 0.318 | 1.507 – 2.593 | -0.678 – 0.604 | **<0.001** | 45.132 |
| Time | 0.150 | 0.246 | 0.177 | 0.290 | -0.353 – 0.653 | -0.417 – 0.770 | 0.548 | 29.293 |
| Condition | 0.300 | 0.467 | 0.354 | 0.551 | -0.641 – 1.241 | -0.756 – 1.464 | 0.524 | 45.132 |
| Prior Camping | 0.674 | 0.499 | 0.795 | 0.588 | -0.327 – 1.675 | -0.386 – 1.976 | 0.183 | 51.108 |
| Time X Condition | 0.850 | 0.410 | 1.004 | 0.484 | 0.010 – 1.690 | 0.012 – 1.995 | **0.047** | 28.557 |
| Time X Prior Camping | -0.073 | 0.446 | -0.087 | 0.526 | -0.984 – 0.837 | -1.161 – 0.988 | 0.870 | 30.154 |
| Condition X Prior Camping | -1.243 | 0.652 | -1.466 | 0.769 | -2.552 – 0.067 | -3.012 – 0.079 | 0.062 | 48.995 |
| Time X Condition X Prior Camping | -0.870 | 0.575 | -1.026 | 0.678 | -2.045 – 0.305 | -2.413 – 0.360 | 0.141 | 29.433 |
| **Random Effects** | | | | | | | | |
| σ^2^ | 0.22 | | | | | | | |
| τ_00_ _ID_Matching_ | 0.37 | | | | | | | |
| ICC | 0.63 | | | | | | | |
| N _ID_Matching_ | 37 | | | | | | | |
| Observations | 69 | | | | | | | |
| Marginal R^2^ / Conditional R^2^ | 0.247 / 0.721 | | | | | | | |

**Table S9.5**

*Results of Linear Mixed Model Predicting IIQ: Engulfment from Prior Camping, Condition, and Time*

|  | **IIQ: Engulfment** | | | | | | | |
| --- | --- | --- | --- | --- | --- | --- | --- | --- |
| *Predictors* | *B* | *SE* | *β* | *Std. SE* | *CI* | *Std. CI* | *p* | *df* |
| (Intercept) | 1.859 | 0.277 | 0.088 | 0.361 | 1.299 – 2.419 | -0.640 – 0.816 | **<0.001** | 41.496 |
| Time | 0.129 | 0.216 | 0.168 | 0.281 | -0.313 – 0.571 | -0.407 – 0.743 | 0.554 | 28.940 |
| Condition | -0.078 | 0.480 | -0.102 | 0.625 | -1.048 – 0.892 | -1.363 – 1.160 | 0.872 | 41.496 |
| Prior Camping | 0.079 | 0.505 | 0.103 | 0.657 | -0.938 – 1.095 | -1.220 – 1.425 | 0.877 | 47.010 |
| Time X Condition | 0.308 | 0.360 | 0.401 | 0.468 | -0.428 – 1.045 | -0.557 – 1.359 | 0.399 | 28.386 |
| Time X Prior Camping | -0.005 | 0.392 | -0.006 | 0.510 | -0.807 – 0.797 | -1.049 – 1.037 | 0.990 | 29.577 |
| Condition X Prior Camping | -0.226 | 0.664 | -0.294 | 0.863 | -1.564 – 1.111 | -2.033 – 1.445 | 0.735 | 44.979 |
| Time X Condition X Prior Camping | -0.394 | 0.505 | -0.512 | 0.657 | -1.426 – 0.639 | -1.855 – 0.831 | 0.442 | 29.044 |
| **Random Effects** | | | | | | | | |
| σ^2^ | 0.17 | | | | | | | |
| τ_00_ _ID_Matching_ | 0.45 | | | | | | | |
| ICC | 0.73 | | | | | | | |
| N _ID_Matching_ | 37 | | | | | | | |
| Observations | 69 | | | | | | | |
| Marginal R^2^ / Conditional R^2^ | 0.049 / 0.744 | | | | | | | |

**Table S9.6**

*Results of Linear Mixed Model Predicting IIQ: Enrichment from Prior Camping, Condition, and Time*

|  | **IIQ: Enrichment** | | | | | | | |
| --- | --- | --- | --- | --- | --- | --- | --- | --- |
| *Predictors* | *B* | *SE* | *β* | *Std. SE* | *CI* | *Std. CI* | *p* | *df* |
| (Intercept) | 2.804 | 0.328 | 0.054 | 0.357 | 2.144 – 3.463 | -0.665 – 0.773 | **<0.001** | 46.527 |
| Time | -0.093 | 0.314 | -0.102 | 0.342 | -0.734 – 0.548 | -0.801 – 0.598 | 0.769 | 29.425 |
| Condition | 0.732 | 0.568 | 0.798 | 0.619 | -0.410 – 1.874 | -0.447 – 2.044 | 0.203 | 46.527 |
| Prior Camping | 0.435 | 0.609 | 0.474 | 0.664 | -0.787 – 1.657 | -0.858 – 1.807 | 0.478 | 52.439 |
| Time X Condition | -0.907 | 0.524 | -0.989 | 0.571 | -1.979 – 0.165 | -2.158 – 0.180 | 0.094 | 28.624 |
| Time X Prior Camping | -0.372 | 0.568 | -0.405 | 0.620 | -1.532 – 0.789 | -1.670 – 0.860 | 0.518 | 30.366 |
| Condition X Prior Camping | -1.356 | 0.794 | -1.478 | 0.866 | -2.951 – 0.240 | -3.218 – 0.261 | 0.094 | 50.384 |
| Time X Condition X Prior Camping | 1.443 | 0.733 | 1.573 | 0.799 | -0.055 – 2.941 | -0.060 – 3.206 | 0.058 | 29.577 |
| **Random Effects** | | | | | | | | |
| σ^2^ | 0.35 | | | | | | | |
| τ_00_ _ID_Matching_ | 0.51 | | | | | | | |
| ICC | 0.59 | | | | | | | |
| N _ID_Matching_ | 37 | | | | | | | |
| Observations | 69 | | | | | | | |
| Marginal R^2^ / Conditional R^2^ | 0.061 / 0.615 | | | | | | | |

**Table S9.7**

*Results of Linear Mixed Model Predicting DIFI: Overlap from Prior Camping, Condition, and Time*

|  | **DIFI: Overlap (social identification)** | | | | | | | |
| --- | --- | --- | --- | --- | --- | --- | --- | --- |
| *Predictors* | *B* | *SE* | *β* | *Std. SE* | *CI* | *Std. CI* | *p* | *df* |
| (Intercept) | 26.110 | 7.014 | -0.265 | 0.166 | 12.261 – 39.959 | -0.594 – 0.063 | **<0.001** | 164.862 |
| Time | -2.839 | 6.529 | -0.067 | 0.155 | -15.799 – 10.120 | -0.375 – 0.240 | 0.665 | 96.232 |
| Condition | 26.772 | 11.525 | 0.635 | 0.273 | 4.021 – 49.523 | 0.095 – 1.174 | **0.021** | 169.234 |
| Prior Camping | -18.814 | 16.728 | -0.446 | 0.397 | -51.841 – 14.213 | -1.229 – 0.337 | 0.262 | 166.335 |
| Time X Condition | -15.226 | 10.887 | -0.361 | 0.258 | -36.827 – 6.375 | -0.873 – 0.151 | 0.165 | 99.242 |
| Time X Prior Camping | 31.169 | 15.584 | 0.739 | 0.370 | 0.237 – 62.101 | 0.006 – 1.472 | **0.048** | 96.720 |
| Condition X Prior Camping | 13.759 | 20.132 | 0.326 | 0.477 | -25.987 – 53.504 | -0.616 – 1.269 | 0.495 | 167.155 |
| Time X Condition X Prior Camping | -9.290 | 18.859 | -0.220 | 0.447 | -46.718 – 28.137 | -1.108 – 0.667 | 0.623 | 97.610 |
| **Random Effects** | | | | | | | | |
| σ^2^ | 719.09 | | | | | | | |
| τ_00_ _ID_Matching_ | 962.18 | | | | | | | |
| ICC | 0.57 | | | | | | | |
| N _ID_Matching_ | 111 | | | | | | | |
| Observations | 207 | | | | | | | |
| Marginal R^2^ / Conditional R^2^ | 0.093 / 0.612 | | | | | | | |

**Table S9.8**

*Results of Linear Mixed Model Predicting CDAT from Prior Camping, Condition, and Time*

|  | **CDAT** | | | | | | | |
| --- | --- | --- | --- | --- | --- | --- | --- | --- |
| *Predictors* | *B* | *SE* | *β* | *Std. SE* | *CI* | *Std. CI* | *p* | *df* |
| (Intercept) | 1.574 | 0.081 | -0.129 | 0.166 | 1.414 – 1.734 | -0.458 – 0.200 | **<0.001** | 147.908 |
| Time | 0.009 | 0.065 | 0.019 | 0.133 | -0.119 – 0.138 | -0.245 – 0.283 | 0.888 | 97.354 |
| Condition | -0.102 | 0.133 | -0.209 | 0.273 | -0.364 – 0.161 | -0.749 – 0.331 | 0.445 | 148.552 |
| Prior Camping | 0.358 | 0.194 | 0.736 | 0.399 | -0.026 – 0.742 | -0.052 – 1.525 | 0.067 | 151.757 |
| Time X Condition | 0.090 | 0.106 | 0.185 | 0.219 | -0.121 – 0.301 | -0.249 – 0.619 | 0.399 | 97.520 |
| Time X Prior Camping | -0.031 | 0.156 | -0.063 | 0.321 | -0.341 – 0.280 | -0.700 – 0.575 | 0.845 | 98.335 |
| Condition X Prior Camping | -0.104 | 0.234 | -0.214 | 0.480 | -0.566 – 0.357 | -1.163 – 0.735 | 0.656 | 151.169 |
| Time X Condition X Prior Camping | -0.108 | 0.188 | -0.223 | 0.386 | -0.481 – 0.264 | -0.989 – 0.543 | 0.565 | 98.186 |
| **Random Effects** | | | | | | | | |
| σ^2^ | 0.07 | | | | | | | |
| τ_00_ _ID_Matching_ | 0.16 | | | | | | | |
| ICC | 0.69 | | | | | | | |
| N _ID_Matching_ | 110 | | | | | | | |
| Observations | 209 | | | | | | | |
| Marginal R^2^ / Conditional R^2^ | 0.049 / 0.704 | | | | | | | |

**Table S9.9**

*Results of Linear Mixed Model Predicting GFD-PCS from Prior Camping, Condition, and Time*

|  | **GFD-PCS** | | | | | | | |
| --- | --- | --- | --- | --- | --- | --- | --- | --- |
| *Predictors* | *B* | *SE* | *β* | *Std. SE* | *CI* | *Std. CI* | *p* | *df* |
| (Intercept) | 4.772 | 0.061 | -0.015 | 0.168 | 4.651 – 4.893 | -0.347 – 0.318 | **<0.001** | 163.625 |
| Time | -0.002 | 0.058 | -0.005 | 0.159 | -0.117 – 0.113 | -0.320 – 0.311 | 0.977 | 97.322 |
| Condition | 0.014 | 0.101 | 0.038 | 0.277 | -0.185 – 0.213 | -0.508 – 0.584 | 0.891 | 164.271 |
| Prior Camping | -0.328 | 0.148 | -0.899 | 0.405 | -0.620 – -0.036 | -1.699 – -0.098 | **0.028** | 167.433 |
| Time X Condition | 0.023 | 0.095 | 0.062 | 0.261 | -0.166 – 0.212 | -0.456 – 0.580 | 0.812 | 97.542 |
| Time X Prior Camping | 0.214 | 0.140 | 0.586 | 0.383 | -0.063 – 0.491 | -0.174 – 1.346 | 0.129 | 98.632 |
| Condition X Prior Camping | 0.341 | 0.178 | 0.936 | 0.488 | -0.010 – 0.693 | -0.027 – 1.899 | 0.057 | 167.125 |
| Time X Condition X Prior Camping | -0.209 | 0.168 | -0.572 | 0.461 | -0.542 – 0.125 | -1.486 – 0.343 | 0.218 | 98.525 |
| **Random Effects** | | | | | | | | |
| σ^2^ | 0.06 | | | | | | | |
| τ_00_ _ID_Matching_ | 0.07 | | | | | | | |
| ICC | 0.56 | | | | | | | |
| N _ID_Matching_ | 110 | | | | | | | |
| Observations | 208 | | | | | | | |
| Marginal R^2^ / Conditional R^2^ | 0.037 / 0.575 | | | | | | | |

**Table S9.10**

*Results of Linear Mixed Model Predicting SWB from Prior Camping, Condition, and Time*

|  | **SWB** | | | | | | | |
| --- | --- | --- | --- | --- | --- | --- | --- | --- |
| *Predictors* | *B* | *SE* | *β* | *Std. SE* | *CI* | *Std. CI* | *p* | *df* |
| (Intercept) | 8.974 | 0.257 | 0.023 | 0.166 | 8.465 – 9.482 | -0.304 – 0.351 | **<0.001** | 139.360 |
| Time | 0.161 | 0.177 | 0.104 | 0.114 | -0.191 – 0.514 | -0.123 – 0.330 | 0.365 | 95.245 |
| Condition | 0.227 | 0.417 | 0.146 | 0.268 | -0.597 – 1.052 | -0.384 – 0.677 | 0.586 | 140.654 |
| Prior Camping | -1.033 | 0.612 | -0.665 | 0.394 | -2.244 – 0.177 | -1.444 – 0.114 | 0.094 | 140.657 |
| Time X Condition | -0.276 | 0.289 | -0.177 | 0.186 | -0.848 – 0.297 | -0.546 – 0.191 | 0.342 | 95.520 |
| Time X Prior Camping | -0.852 | 0.424 | -0.548 | 0.273 | -1.693 – -0.010 | -1.089 – -0.007 | **0.047** | 95.521 |
| Condition X Prior Camping | 0.827 | 0.735 | 0.532 | 0.473 | -0.625 – 2.280 | -0.402 – 1.467 | 0.262 | 140.759 |
| Time X Condition X Prior Camping | 0.936 | 0.509 | 0.602 | 0.328 | -0.074 – 1.947 | -0.048 – 1.253 | 0.069 | 95.612 |
| **Random Effects** | | | | | | | | |
| σ^2^ | 0.53 | | | | | | | |
| τ_00_ _ID_Matching_ | 1.81 | | | | | | | |
| ICC | 0.78 | | | | | | | |
| N _ID_Matching_ | 111 | | | | | | | |
| Observations | 208 | | | | | | | |
| Marginal R^2^ / Conditional R^2^ | 0.064 / 0.790 | | | | | | | |

**Table S9.11**

*Results of Linear Mixed Model Predicting CDPQOL: Enjoyment (Ages 8-12 years) from Prior Camping, Condition, and Time*

|  | **CDPQOL: Enjoyment (Ages 8-12 years)** | | | | | | | |
| --- | --- | --- | --- | --- | --- | --- | --- | --- |
| *Predictors* | *B* | *SE* | *β* | *Std. SE* | *CI* | *Std. CI* | *p* | *df* |
| (Intercept) | 1.277 | 0.114 | -0.156 | 0.189 | 1.052 – 1.502 | -0.531 – 0.219 | **<0.001** | 111.419 |
| Time | 0.006 | 0.108 | 0.010 | 0.180 | -0.209 – 0.222 | -0.349 – 0.370 | 0.954 | 64.485 |
| Condition | 0.247 | 0.187 | 0.412 | 0.312 | -0.124 – 0.618 | -0.206 – 1.030 | 0.189 | 112.853 |
| Prior Camping | 0.848 | 0.316 | 1.413 | 0.527 | 0.221 – 1.475 | 0.368 – 2.459 | **0.009** | 103.572 |
| Time X Condition | -0.280 | 0.178 | -0.467 | 0.297 | -0.636 – 0.075 | -1.060 – 0.125 | 0.120 | 65.004 |
| Time X Prior Camping | -0.506 | 0.299 | -0.844 | 0.499 | -1.104 – 0.092 | -1.841 – 0.153 | 0.096 | 61.802 |
| Condition X Prior Camping | -0.993 | 0.373 | -1.656 | 0.622 | -1.733 – -0.254 | -2.889 – -0.423 | **0.009** | 105.834 |
| Time X Condition X Prior Camping | 0.788 | 0.353 | 1.314 | 0.589 | 0.082 – 1.494 | 0.137 – 2.491 | **0.029** | 62.554 |
| **Random Effects** | | | | | | | | |
| σ^2^ | 0.16 | | | | | | | |
| τ_00_ _ID_Matching_ | 0.19 | | | | | | | |
| ICC | 0.55 | | | | | | | |
| N _ID_Matching_ | 74 | | | | | | | |
| Observations | 139 | | | | | | | |
| Marginal R^2^ / Conditional R^2^ | 0.070 / 0.585 | | | | | | | |

**Table S9.12**

*Results of Linear Mixed Model Predicting CDPQOL: Negative Emotions (Ages 8-12 years) from Prior Camping, Condition, and Time*

|  | **CDPQOL: Negative Emotions (Ages 8-12 years)** | | | | | | | |
| --- | --- | --- | --- | --- | --- | --- | --- | --- |
| *Predictors* | *B* | *SE* | *β* | *Std. SE* | *CI* | *Std. CI* | *p* | *df* |
| (Intercept) | 1.828 | 0.120 | -0.103 | 0.193 | 1.589 – 2.066 | -0.485 – 0.280 | **<0.001** | 111.754 |
| Time | 0.091 | 0.114 | 0.145 | 0.183 | -0.137 – 0.318 | -0.220 – 0.510 | 0.430 | 65.457 |
| Condition | -0.015 | 0.195 | -0.024 | 0.313 | -0.402 – 0.372 | -0.645 – 0.596 | 0.938 | 110.806 |
| Prior Camping | 0.375 | 0.335 | 0.601 | 0.538 | -0.290 – 1.040 | -0.465 – 1.668 | 0.266 | 103.833 |
| Time X Condition | -0.101 | 0.185 | -0.162 | 0.297 | -0.471 – 0.268 | -0.755 – 0.430 | 0.586 | 65.122 |
| Time X Prior Camping | 0.064 | 0.316 | 0.103 | 0.507 | -0.567 – 0.695 | -0.910 – 1.116 | 0.840 | 62.757 |
| Condition X Prior Camping | -0.414 | 0.394 | -0.664 | 0.632 | -1.195 – 0.367 | -1.917 – 0.589 | 0.295 | 105.439 |
| Time X Condition X Prior Camping | 0.174 | 0.371 | 0.278 | 0.596 | -0.569 – 0.916 | -0.912 – 1.469 | 0.642 | 63.289 |
| **Random Effects** | | | | | | | | |
| σ^2^ | 0.17 | | | | | | | |
| τ_00_ _ID_Matching_ | 0.22 | | | | | | | |
| ICC | 0.56 | | | | | | | |
| N _ID_Matching_ | 74 | | | | | | | |
| Observations | 140 | | | | | | | |
| Marginal R^2^ / Conditional R^2^ | 0.038 / 0.574 | | | | | | | |

**Table S9.13**

*Results of Linear Mixed Model Predicting CDPQOL: School (Ages 8-12 years) from Prior Camping, Condition, and Time*

|  | **CDPQOL: School (Ages 8-12 years)** | | | | | | | |
| --- | --- | --- | --- | --- | --- | --- | --- | --- |
| *Predictors* | *B* | *SE* | *β* | *Std. SE* | *CI* | *Std. CI* | *p* | *df* |
| (Intercept) | 1.654 | 0.118 | 0.035 | 0.193 | 1.419 – 1.888 | -0.347 – 0.417 | **<0.001** | 106.374 |
| Time | -0.054 | 0.102 | -0.087 | 0.166 | -0.257 – 0.150 | -0.419 – 0.244 | 0.601 | 63.462 |
| Condition | -0.111 | 0.193 | -0.180 | 0.314 | -0.494 – 0.272 | -0.804 – 0.443 | 0.568 | 106.374 |
| Prior Camping | 0.159 | 0.328 | 0.259 | 0.534 | -0.493 – 0.811 | -0.802 – 1.320 | 0.629 | 96.296 |
| Time X Condition | -0.017 | 0.166 | -0.028 | 0.271 | -0.350 – 0.315 | -0.569 – 0.513 | 0.919 | 63.462 |
| Time X Prior Camping | 0.429 | 0.278 | 0.698 | 0.452 | -0.127 – 0.984 | -0.206 – 1.601 | 0.128 | 60.701 |
| Condition X Prior Camping | -0.141 | 0.386 | -0.229 | 0.629 | -0.907 – 0.626 | -1.477 – 1.019 | 0.717 | 98.445 |
| Time X Condition X Prior Camping | -0.192 | 0.328 | -0.312 | 0.534 | -0.848 – 0.464 | -1.380 – 0.755 | 0.561 | 61.291 |
| **Random Effects** | | | | | | | | |
| σ^2^ | 0.13 | | | | | | | |
| τ_00_ _ID_Matching_ | 0.24 | | | | | | | |
| ICC | 0.64 | | | | | | | |
| N _ID_Matching_ | 74 | | | | | | | |
| Observations | 138 | | | | | | | |
| Marginal R^2^ / Conditional R^2^ | 0.042 / 0.660 | | | | | | | |

**Table S9.14**

*Results of Linear Mixed Model Predicting CDPQOL: Isolation (Ages 13-16 years) from Prior Camping, Condition, and Time*

|  | **CDPQOL: Isolation (Ages 13-16 years)** | | | | | | | |
| --- | --- | --- | --- | --- | --- | --- | --- | --- |
| *Predictors* | *B* | *SE* | *β* | *Std. SE* | *CI* | *Std. CI* | *p* | *df* |
| (Intercept) | 2.000 | 0.230 | 0.233 | 0.366 | 1.538 – 2.462 | -0.504 – 0.970 | **<0.001** | 44.769 |
| Time | 0.000 | 0.207 | -0.000 | 0.329 | -0.422 – 0.422 | -0.673 – 0.673 | 1.000 | 29.259 |
| Condition | -0.125 | 0.398 | -0.199 | 0.633 | -0.926 – 0.676 | -1.475 – 1.077 | 0.755 | 44.769 |
| Prior Camping | -0.296 | 0.424 | -0.472 | 0.675 | -1.147 – 0.555 | -1.828 – 0.884 | 0.488 | 50.741 |
| Time X Condition | 0.062 | 0.345 | 0.100 | 0.549 | -0.643 – 0.768 | -1.024 – 1.224 | 0.857 | 28.539 |
| Time X Prior Camping | 0.546 | 0.375 | 0.870 | 0.597 | -0.219 – 1.311 | -0.349 – 2.090 | 0.155 | 30.098 |
| Condition X Prior Camping | 0.145 | 0.554 | 0.231 | 0.883 | -0.969 – 1.259 | -1.544 – 2.006 | 0.795 | 48.620 |
| Time X Condition X Prior Camping | -0.551 | 0.483 | -0.877 | 0.769 | -1.537 – 0.436 | -2.450 – 0.695 | 0.263 | 29.395 |
| **Random Effects** | | | | | | | | |
| σ^2^ | 0.15 | | | | | | | |
| τ_00_ _ID_Matching_ | 0.27 | | | | | | | |
| ICC | 0.64 | | | | | | | |
| N _ID_Matching_ | 37 | | | | | | | |
| Observations | 69 | | | | | | | |
| Marginal R^2^ / Conditional R^2^ | 0.048 / 0.656 | | | | | | | |

**Table S9.15**

*Results of Linear Mixed Model Predicting CDPQOL: Limitations (Ages 13-16 years) from Prior Camping, Condition, and Time*

|  | **CDPQOL: Limitations (Ages 13-16 years)** | | | | | | | |
| --- | --- | --- | --- | --- | --- | --- | --- | --- |
| *Predictors* | *B* | *SE* | *β* | *Std. SE* | *CI* | *Std. CI* | *p* | *df* |
| (Intercept) | 2.208 | 0.325 | -0.039 | 0.330 | 1.550 – 2.867 | -0.707 – 0.630 | **<0.001** | 38.521 |
| Time | 0.062 | 0.208 | 0.063 | 0.212 | -0.365 – 0.488 | -0.370 – 0.496 | 0.770 | 28.634 |
| Condition | 0.042 | 0.563 | 0.042 | 0.572 | -1.098 – 1.182 | -1.116 – 1.200 | 0.941 | 38.521 |
| Prior Camping | 1.014 | 0.584 | 1.030 | 0.593 | -0.164 – 2.192 | -0.167 – 2.226 | 0.090 | 42.867 |
| Time X Condition | 0.188 | 0.347 | 0.191 | 0.352 | -0.521 – 0.898 | -0.529 – 0.912 | 0.591 | 28.250 |
| Time X Prior Camping | 0.383 | 0.379 | 0.389 | 0.385 | -0.393 – 1.158 | -0.399 – 1.176 | 0.321 | 29.069 |
| Condition X Prior Camping | -1.103 | 0.772 | -1.120 | 0.784 | -2.661 – 0.455 | -2.703 – 0.462 | 0.160 | 41.221 |
| Time X Condition X Prior Camping | -0.873 | 0.487 | -0.887 | 0.495 | -1.870 – 0.124 | -1.899 – 0.126 | 0.084 | 28.705 |
| **Random Effects** | | | | | | | | |
| σ^2^ | 0.15 | | | | | | | |
| τ_00_ _ID_Matching_ | 0.69 | | | | | | | |
| ICC | 0.82 | | | | | | | |
| N _ID_Matching_ | 37 | | | | | | | |
| Observations | 69 | | | | | | | |
| Marginal R^2^ / Conditional R^2^ | 0.190 / 0.853 | | | | | | | |

**Table S9.16**

*Results of Linear Mixed Model Predicting CDPQOL: Limitations (Ages 13-16 years) from Prior Camping, Condition, and Time*

|  | **CDPQOL: Social (Ages 13-16 years)** | | | | | | | |
| --- | --- | --- | --- | --- | --- | --- | --- | --- |
| *Predictors* | *B* | *SE* | *β* | *Std. SE* | *CI* | *Std. CI* | *p* | *df* |
| (Intercept) | 2.125 | 0.272 | 0.122 | 0.330 | 1.575 – 2.675 | -0.547 – 0.790 | **<0.001** | 38.278 |
| Time | 0.142 | 0.170 | 0.173 | 0.207 | -0.206 – 0.491 | -0.251 – 0.597 | 0.410 | 28.608 |
| Condition | -0.161 | 0.470 | -0.195 | 0.572 | -1.113 – 0.791 | -1.353 – 0.962 | 0.735 | 38.278 |
| Prior Camping | 0.764 | 0.487 | 0.929 | 0.592 | -0.219 – 1.746 | -0.266 – 2.123 | 0.124 | 42.493 |
| Time X Condition | -0.107 | 0.283 | -0.130 | 0.345 | -0.687 – 0.474 | -0.835 – 0.576 | 0.709 | 28.239 |
| Time X Prior Camping | 0.005 | 0.310 | 0.006 | 0.377 | -0.630 – 0.639 | -0.766 – 0.777 | 0.988 | 29.025 |
| Condition X Prior Camping | -0.822 | 0.644 | -0.999 | 0.783 | -2.122 – 0.478 | -2.580 – 0.582 | 0.209 | 40.893 |
| Time X Condition X Prior Camping | -0.198 | 0.398 | -0.241 | 0.484 | -1.013 – 0.617 | -1.232 – 0.751 | 0.623 | 28.677 |
| **Random Effects** | | | | | | | | |
| σ^2^ | 0.10 | | | | | | | |
| τ_00_ _ID_Matching_ | 0.49 | | | | | | | |
| ICC | 0.83 | | | | | | | |
| N _ID_Matching_ | 37 | | | | | | | |
| Observations | 69 | | | | | | | |
| Marginal R^2^ / Conditional R^2^ | 0.178 / 0.857 | | | | | | | |

**Table S9.17**

*Results of Linear Mixed Model Predicting CDPQOL: Uncertainty (Ages 13-16 years) from Prior Camping, Condition, and Time*

|  | **CDPQOL: Uncertainty (Ages 13-16 years)** | | | | | | | |
| --- | --- | --- | --- | --- | --- | --- | --- | --- |
| *Predictors* | *B* | *SE* | *β* | *Std. SE* | *CI* | *Std. CI* | *p* | *df* |
| (Intercept) | 1.708 | 0.275 | -0.200 | 0.353 | 1.151 – 2.266 | -0.915 – 0.514 | **<0.001** | 37.307 |
| Time | 0.239 | 0.157 | 0.306 | 0.201 | -0.083 – 0.561 | -0.106 – 0.718 | 0.139 | 28.503 |
| Condition | 0.375 | 0.477 | 0.481 | 0.611 | -0.590 – 1.340 | -0.757 – 1.718 | 0.436 | 37.307 |
| Prior Camping | 0.879 | 0.491 | 1.127 | 0.629 | -0.112 – 1.870 | -0.144 – 2.397 | 0.081 | 40.947 |
| Time X Condition | -0.489 | 0.261 | -0.627 | 0.335 | -1.024 – 0.046 | -1.312 – 0.059 | 0.072 | 28.195 |
| Time X Prior Camping | -0.826 | 0.286 | -1.059 | 0.367 | -1.412 – -0.241 | -1.809 – -0.309 | **0.007** | 28.850 |
| Condition X Prior Camping | -1.050 | 0.650 | -1.346 | 0.833 | -2.364 – 0.264 | -3.030 – 0.338 | 0.114 | 39.556 |
| Time X Condition X Prior Camping | 0.815 | 0.367 | 1.044 | 0.471 | 0.063 – 1.567 | 0.081 – 2.008 | **0.035** | 28.560 |
| **Random Effects** | | | | | | | | |
| σ^2^ | 0.09 | | | | | | | |
| τ_00_ _ID_Matching_ | 0.52 | | | | | | | |
| ICC | 0.86 | | | | | | | |
| N _ID_Matching_ | 37 | | | | | | | |
| Observations | 69 | | | | | | | |
| Marginal R^2^ / Conditional R^2^ | 0.069 / 0.866 | | | | | | | |

# Robustness analysis

**Table S10**

*Results of Multiple Wilcoxon Signed Rank Tests for the Difference Between Pretest and Posttest Scores Withing Prior Camping Groups and Conditions*

|  | **Campers** | | **Noncampers** | | **Novice noncampers** | | **Novice campers** | | **Experienced noncampers** | | **Experienced campers** | |
| --- | --- | --- | --- | --- | --- | --- | --- | --- | --- | --- | --- | --- |
| **Variable** | **V** | **p** | **V** | **p** | **V** | **p** | **V** | **p** | **V** | **p** | **V** | **p** |
| CD Friendships | 103 | <.001 | 82 | 0.803 | 45.5 | 1 | 3 | <.001 | 6.5 | 0.713 | 66 | 0.009 |
| Peer Support | 78.5 | 0.603 | 29 | 0.475 | 17.5 | 0.611 | 3 | 0.625 | 2 | 1 | 55.5 | 0.206 |
| IIQ: Rejection | 129 | 0.177 | 27 | 0.635 | 15.5 | 0.866 | 10 | 0.098 | 2 | 1 | 63.5 | 0.864 |
| IIQ: Acceptance | 63.5 | 0.864 | 16 | 0.261 | 3 | 0.074 | 0 | 0.346 | 3 | 1 | 52.5 | 0.648 |
| IIQ: Engulfment | 142.5 | 0.354 | 32.5 | 0.258 | 15 | 0.399 | 6 | 0.174 | 3 | 1 | 92 | 0.792 |
| IIQ: Enrichment | 110.5 | 0.614 | 18 | 0.635 | 12 | 0.799 | 0 | 0.125 | 1 | 1 | 96 | 0.663 |
| DIFI - Overlap | 255.5 | 0.226 | 141 | 0.649 | 82 | 0.896 | 4 | 0.011 | 9 | 0.201 | 171 | 0.83 |
| GFD-PCS | 117 | 0.662 | 147 | 0.509 | 75.5 | 0.981 | 18.5 | 1 | 13.5 | 0.134 | 45.5 | 0.633 |
| CDAT | 627 | 0.692 | 225 | 0.885 | 159 | 0.936 | 95.5 | 0.161 | 7.5 | 1 | 240 | 0.659 |
| CDPQOL: Negative emotions | 434 | 0.02 | 196.5 | 0.367 | 135.5 | 0.497 | 77 | 0.132 | 7 | 0.625 | 153.5 | 0.073 |
| CDPQOL: School | 196 | 0.368 | 207.5 | 0.658 | 145 | 0.895 | 30.5 | 0.523 | 6 | 0.181 | 70 | 0.09 |
| CDPQOL: Enjoyment | 38 | 0.364 | 27.5 | 0.649 | 27.5 | 1 | 1.5 | 0.134 | 0 | 1 | 25 | 0.803 |
| CDPQOL: Social | 67 | 0.267 | 30.5 | 0.092 | 13 | 0.176 | 4.5 | 0.586 | 5 | 0.5 | 41.5 | 0.178 |
| CDPQOL: Uncertainty | 29 | 0.045 | 9.5 | 0.916 | 6 | 0.181 | 2.5 | 0.461 | 0 | 0.174 | 15 | 0.064 |
| CDPQOL: Isolation | 93.5 | 0.423 | 38 | 0.304 | 14 | 1 | 6 | 0.85 | 6 | 0.174 | 55 | 0.522 |
| CDPQOL: Limitation | 39.5 | 0.254 | 15 | 0.402 | 9 | 0.787 | 3 | 0.371 | 1 | 1 | 20.5 | 0.086 |
| SWB | 220 | 0.792 | 110.5 | 0.516 | 71.5 | 0.21 | 23 | 0.666 | 5 | 0.572 | 105 | 1 |

Note. CD = Celiac disease. IIQ = Illness Identity Questionnaire. DIFI = Dynamic Identity Fusion Index. CFD-PCS = Gluten-Free Diet Perceived Competence Scale. CDAT = Celiac Dietary Adherence Test. CDPQOL = Pediatric Celiac Disease Quality of Life (measured with two versions, age-dependent, 8-12 years: n = 75, 13-16: n = 36). SWB = Subjective Well-Being. Peer support and IIQ outcomes were measured only for participants aged 13–16 years (n = 36). The values of the V statistics and the corresponding p value refer to the difference between pretest and posttest scores within groups of prior camping experience and conditions.

* *p* < .05, ** *p* < .01, *** *p* < .001.

**Table S11**

*Mixed-Effects Linear Models Predicting Psychosocial Outcomes by Prior Celiac Camp Experience, Condition, and Time, Controlling for Covariates*

| Outcome | Group | Campers pretest *M* (*SD*) | Campers posttest *M* (*SD*) | Noncampers pretest  *M* (*SD*) | Noncampers posttest  *M* (*SD*) | Cohen's *d* for Campers | Cohen's *d* for Noncampers | Interaction *B* (*SE*) | *p*-value |
| --- | --- | --- | --- | --- | --- | --- | --- | --- | --- |
| CD Friendships | All sample | 2.25 (0.09)*** | 3.06 (0.10) | 1.99 (0.14) | 2.03 (0.15) | 1.71 | 0.10 | -1.20 (0.19) | < .001 |
|  | Experienced | 2.72 (0.12)*** | 3.13 (0.12) | 2.04 (0.24) | 2.13 (0.26) | 0.86 | 0.19 | 0.89 (0.35) | .013 |
|  | Novice | 1.78 (0.14)*** | 2.99 (0.15) | 1.93 (0.12) | 1.94 (0.12) | 2.56 | 0.01 |  |  |
| Peer support | All sample | 3.15 (0.34) | 3.30 (0.34) | 2.92 (0.37) | 3.67 (0.39) | 0.35 | -0.58 | -0.36 (0.40) | .38 |
|  | Experienced | 3.36 (0.28) | 3.25 (0.28) | 2.61 (0.56) | 2.04 (0.60) | -0.25 | -1.30 | -0.11 (0.61) | .87 |
|  | Novice | 2.93 (0.56) | 3.35 (0.56) | 3.23 (0.44) | 3.29 (0.43) | 0.95 | 0.14 |  |  |
| IIQ-Rejection | All sample | 2.61 (0.29)** | 2.11 (0.29) | 2.37 (0.32) | 2.28 (0.33) | -1.28 | -0.22 | 1.12 (0.35) | .004 |
|  | Experienced | 1.85 (0.24) | 1.86 (0.24) | 2.68 (0.47) | 2.39 (0.52) | 0.02 | -0.75 | -1.42 (0.54) | .014 |
|  | Novice | 3.37 (0.48)*** | 2.37 (0.48) | 2.06 (0.38) | 2.18 (0.37) | -2.59 | 0.31 |  |  |
| IIQ-Acceptance | All sample | 4.46 (0.38) | 4.46 (0.38) | 3.99 (0.45) | 4.22 (0.46) | -0.01 | 0.60 | 0.20 (0.44) | .65 |
|  | Experienced | 4.45 (0.25) | 4.34 (0.26) | 4.1 (0.5) | 4.23 (0.54) | -0.28 | 0.34 | 0.01 (0.53) | .99 |
|  | Novice | 4.47 (0.5) | 4.57 (0.5) | 3.89 (0.4) | 4.22 (0.39) | 0.26 | 0.87 |  |  |
| IIQ-Engulfment | All sample | 1.88 (0.37) | 1.64 (0.37) | 1.95 (0.44) | 1.81 (0.46) | -0.55 | -0.33 | 0.37 (0.40) | .36 |
|  | Experienced | 1.67 (0.24) | 1.63 (0.25) | 2.14 (0.49) | 1.92 (0.54) | -0.10 | -0.50 | -0.55 (0.60) | .37 |
|  | Novice | 2.09 (0.49) | 1.65 (0.49) | 1.76 (0.39) | 1.69 (0.38) | -1.01 | -0.15 |  |  |
| IIQ-Enrichment | All sample | 2.62 (0.35)* | 3.09 (0.36) | 2.84 (0.43) | 3.11 (0.46) | 0.80 | 0.46 | -0.94 (0.54) | .092 |
|  | Experienced | 2.74 (0.23) | 2.68 (0.24) | 2.81 (0.47) | 3.3 (0.56) | -0.10 | 0.83 | 1.49 (0.81) | .079 |
|  | Novice | 2.5 (0.47)* | 3.5 (0.47) | 2.87 (0.38) | 2.93 (0.36) | 1.69 | 0.10 |  |  |
| DIFI | All sample | 41.26 (8.35) | 50.28 (8.74) | 30.11 (11.64)^♰^ | 15.23 (12.13) | 0.34 | -0.56 | -15.92 (11.11) | .16 |
|  | Experienced | 48.57 (7.62) | 47.65 (7.85) | 40.82 (15.58)* | 8.02 (16.38) | -0.03 | -1.23 | -15.96 (19.78) | .42 |
|  | Novice | 33.95 (9.08)* | 52.91 (9.63) | 19.4 (7.7) | 22.44 (7.89) | 0.71 | 0.11 |  |  |
| GFD-PCS | All sample | 4.84 (0.07) | 4.82 (0.08) | 4.81 (0.1) | 4.71 (0.11) | -0.08 | -0.40 | 0.03 (0.10) | .79 |
|  | Experienced | 4.88 (0.07) | 4.86 (0.07) | 4.82 (0.14) | 4.61 (0.14) | -0.07 | -0.82 | -0.22 (0.18) | .25 |
|  | Novice | 4.8 (0.08) | 4.78 (0.08) | 4.8 (0.07) | 4.81 (0.07) | -0.10 | 0.01 |  |  |
| CDAT | All sample | 1.63 (0.09) | 1.62 (0.09) | 1.71 (0.13) | 1.71 (0.13) | -0.02 | 0.03 | 0.05 (0.11) | .69 |
|  | Experienced | 1.65 (0.08) | 1.72 (0.08) | 1.73 (0.17) | 1.78 (0.18) | 0.26 | 0.19 | -0.06 (0.20) | .75 |
|  | Novice | 1.61 (0.1) | 1.53 (0.1) | 1.68 (0.08) | 1.65 (0.08) | -0.30 | -0.14 |  |  |
| SWB | All sample | 8.94 (0.31) | 9.03 (0.32) | 8.19 (0.44) | 8.42 (0.45) | 0.13 | 0.32 | -0.28 (0.30) | .36 |
|  | Experienced | 8.96 (0.29) | 9.04 (0.29) | 7.51 (0.58) | 8.14 (0.6) | 0.10 | 0.86 | 0.83 (0.54) | .13 |
|  | Novice | 8.92 (0.34) | 9.03 (0.35) | 8.87 (0.29) | 8.71 (0.29) | 0.16 | -0.22 |  |  |

*Note*. CD = Celiac disease. IIQ = Illness Identity Questionnaire. DIFI = Dynamic Identity Fusion Index. CFD-PCS = Gluten-Free Diet Perceived Competence Scale. CDAT = Celiac Dietary Adherence Test. SWB = Subjective Well-Being. Peer support and IIQ outcomes were measured only for participants aged 13–16 years (*n* = 36). The effects are adjusted to gender (1 = female), age, age of diagnosis, socioeconomic status, and having other health conditions (1 = yes). Cohen’s *d* values indicate effect sizes, with positive and negative signs reflecting the direction of effects. Tukey’s pairwise comparisons were conducted using Kenward-Roger degrees of freedom. Values in parentheses are robust standard errors. Interaction terms for the “All sample” rows represent the two-way interaction between condition and time, while interaction terms for the “Experienced” rows represent the three-way interaction between prior camp experience, condition, and time.
♰ p < .10, * p < .05, ** p < .01, *** p < .001.

**Table S12**

*Mixed-Effects Linear Models Predicting CDPQOL by Prior Celiac Camp Experience, Condition, and Time, Controlling for Covariates*

| Outcome | Group | Campers pretest *M* (*SD*) | Campers posttest *M* (*SD*) | Noncampers pretest  *M* (*SD*) | Noncampers posttest  *M* (*SD*) | Cohen's *d* for Campers | Cohen's *d* for Noncampers | Interaction *B* (*SE*) | *p*-value |
| --- | --- | --- | --- | --- | --- | --- | --- | --- | --- |
| CDPQOL: Children aged 8-12 years (*n* = 75) | | | | | | | | | |
| Negative emotions | All sample | 1.88 (0.16) | 1.81 (0.17) | 2.21 (0.23) | 2.08 (0.23) | -0.16 | -0.31 | -0.14 (0.19) | .46 |
|  | Experienced | 1.95 (0.16) | 1.78 (0.16) | 2.43 (0.33) | 2.27 (0.33) | -0.42 | -0.38 | 0.16 (0.37) | .67 |
|  | Novice | 1.80 (0.17) | 1.84 (0.17) | 2.00 (0.13) | 1.90 (0.14) | 0.10 | -0.24 |  |  |
| School | All sample | 1.58 (0.16) | 1.54 (0.16) | 1.95 (0.22) | 1.80 (0.22) | -0.11 | -0.42 | 0.01 (0.17) | .97 |
|  | Experienced | 1.67 (0.15) | 1.53 (0.16) | 2.27 (0.31) | 1.89 (0.31) | -0.38 | -1.01 | -0.24 (0.33) | .47 |
|  | Novice | 1.49 (0.16) | 1.55 (0.17) | 1.63 (0.13) | 1.70 (0.13) | 0.16 | 0.18 |  |  |
| Enjoyment | All sample | 1.30 (0.15) | 1.44 (0.16) | 1.46 (0.21) | 1.70 (0.21) | 0.35 | 0.60 | -0.30 (0.19) | .11 |
|  | Experienced | 1.32 (0.14) | 1.31 (0.15) | 1.63 (0.29) ^♰^ | 2.13 (0.29) | -0.03 | 1.24 | 0.82 (0.37) | .029 |
|  | Novice | 1.29 (0.15) ^♰^ | 1.58 (0.16) | 1.29 (0.12) | 1.27 (0.12) | 0.72 | -0.04 |  |  |
| CDPQOL: Adolescents aged 13-16 years (*n* = 36) | | | | | | | | | |
| Social | All sample | 1.7 (0.38) | 1.78 (0.38) | 2.52 (0.45) | 2.4 (0.46) | 0.23 | -0.36 | -0.08 (0.31) | .80 |
|  | Experienced | 1.71 (0.25) | 1.9 (0.26) | 3.08 (0.5) | 2.95 (0.53) | 0.56 | -0.38 | -0.24 (0.48) | .62 |
|  | Novice | 1.68 (0.5) | 1.65 (0.5) | 1.97 (0.4) | 1.85 (0.39) | -0.11 | -0.35 |  |  |
| Uncertainty | All sample | 1.5 (0.37)** | 1.77 (0.37) | 1.65 (0.43) ^♰^ | 1.95 (0.44) | 0.94 | 1.06 | -0.36 (0.26) | .19 |
|  | Experienced | 1.51 (0.25)** | 1.8 (0.25) | 1.87 (0.48)** | 2.59 (0.51) | 1.01 | 2.50 | 0.79 (0.40) | .061 |
|  | Novice | 1.49 (0.49) | 1.74 (0.49) | 1.43 (0.38) | 1.32 (0.38) | 0.87 | -0.38 |  |  |
| Isolation | All sample | 1.79 (0.3) | 1.72 (0.3) | 1.92 (0.36) | 1.8 (0.38) | -0.18 | -0.33 | 0.27 (0.34) | .43 |
|  | Experienced | 1.78 (0.2) | 1.71 (0.2) | 2.2 (0.4) | 1.75 (0.45) | -0.19 | -1.22- | -0.66 (0.52) | .21 |
|  | Novice | 1.8 (0.4) | 1.73 (0.4) | 1.64 (0.32) | 1.85 (0.31) | -0.17 | 0.57 |  |  |
| Limitation | All sample | 1.92 (0.44) | 1.96 (0.44) | 2.75 (0.52) | 2.46 (0.53) | 0.09 | -0.77 | 0.29 (0.34) | .41 |
|  | Experienced | 1.75 (0.29)* | 2.07 (0.29) | 3.73 (0.57) | 3.11 (0.61) | 0.84 | -1.64 | -1.23 (0.53) | .028 |
|  | Novice | 2.09 (0.58) | 1.84 (0.58) | 1.76 (0.46) | 1.8 (0.45) | -0.66 | 0.10 |  |  |

*Note*. CDPQOL = Pediatric Celiac Disease Quality of Life (measured with two versions, age-dependent). The effects are adjusted to gender (1 = female), age, age of diagnosis, socioeconomic status, and having other health conditions (1 = yes). Cohen’s *d* values indicate effect sizes, with positive and negative signs showing the direction of effects. Tukey’s pairwise comparisons were conducted using Kenward-Roger degrees of freedom. Values in parentheses are robust standard errors. Interaction terms for the "All sample" rows represent the two-way interaction between condition and time, while interaction terms for the "Experienced" rows represent the three-way interaction between prior camping experience, condition, and time.
♰ *p* < .10, * *p* < .05, ** *p* < .01, *** *p* < .001.

**Table S13.1**

*Results of Linear Mixed Model Predicting CD Friendships from Prior Camping, Condition, and Time, Controlling for Covariates*

|  | **CD Friendship** | | | | | | | |
| --- | --- | --- | --- | --- | --- | --- | --- | --- |
| *Predictors* | *B* | *SE* | *β* | *Std. SE* | *CI* | *Std. CI* | *p* | *df* |
| (Intercept) | 0.996 | 0.470 | -0.618 | 0.163 | 0.063 – 1.928 | -0.940 – -0.295 | **0.037** | 125.462 |
| Time | -0.005 | 0.117 | -0.005 | 0.129 | -0.237 – 0.228 | -0.262 – 0.251 | 0.967 | 89.833 |
| Condition | 1.054 | 0.187 | 1.162 | 0.206 | 0.684 – 1.423 | 0.754 – 1.569 | **<0.001** | 159.634 |
| Prior Camping | 0.196 | 0.281 | 0.216 | 0.310 | -0.359 – 0.750 | -0.396 – 0.827 | 0.487 | 158.208 |
| Gender (1 = female) | 0.116 | 0.123 | 0.128 | 0.136 | -0.129 – 0.360 | -0.142 – 0.397 | 0.349 | 92.270 |
| Age | 0.125 | 0.029 | 0.288 | 0.067 | 0.067 – 0.182 | 0.155 – 0.421 | **<0.001** | 92.300 |
| Age of diagnosis | -0.012 | 0.019 | -0.041 | 0.061 | -0.049 – 0.025 | -0.163 – 0.082 | 0.511 | 91.576 |
| SES | -0.072 | 0.046 | -0.097 | 0.061 | -0.163 – 0.019 | -0.219 – 0.025 | 0.117 | 92.602 |
| Other health conditions | 0.052 | 0.135 | 0.057 | 0.149 | -0.216 – 0.320 | -0.238 – 0.353 | 0.700 | 95.683 |
| Time X Condition | -1.202 | 0.193 | -1.324 | 0.213 | -1.585 – -0.818 | -1.748 – -0.901 | **<0.001** | 92.775 |
| Time X Prior Camping | -0.086 | 0.292 | -0.095 | 0.322 | -0.666 – 0.494 | -0.734 – 0.544 | 0.768 | 92.512 |
| Condition X Prior Camping | -0.053 | 0.336 | -0.058 | 0.371 | -0.717 – 0.611 | -0.791 – 0.674 | 0.875 | 159.735 |
| Time X Condition X Prior Camping | 0.885 | 0.348 | 0.975 | 0.384 | 0.193 – 1.576 | 0.213 – 1.737 | **0.013** | 92.876 |
| **Random Effects** | | | | | | | | |
| σ^2^ | 0.22 | | | | | | | |
| τ_00_ _ID_Matching_ | 0.18 | | | | | | | |
| ICC | 0.45 | | | | | | | |
| N _ID_Matching_ | 102 | | | | | | | |
| Observations | 193 | | | | | | | |
| Marginal R^2^ / Conditional R^2^ | 0.528 / 0.740 | | | | | | | |

**Table S13.2**

*Results of Linear Mixed Model Predicting Peer Support from Prior Camping, Condition, and Time, Controlling for Covariates*

|  | **Peer Support** | | | | | | | |
| --- | --- | --- | --- | --- | --- | --- | --- | --- |
| *Predictors* | *B* | *SE* | *β* | *Std. SE* | *CI* | *Std. CI* | *p* | *df* |
| (Intercept) | 2.710 | 2.844 | 0.589 | 0.570 | -3.173 – 8.593 | -0.584 – 1.762 | 0.351 | 24.988 |
| Time | -0.062 | 0.253 | -0.068 | 0.280 | -0.582 – 0.459 | -0.646 – 0.509 | 0.809 | 24.566 |
| Condition | 0.057 | 0.607 | 0.064 | 0.673 | -1.187 – 1.302 | -1.316 – 1.444 | 0.925 | 27.526 |
| Prior Camping | -1.255 | 0.708 | -1.391 | 0.785 | -2.694 – 0.184 | -2.986 – 0.204 | 0.085 | 33.723 |
| Gender (1 = female) | -0.518 | 0.434 | -0.574 | 0.481 | -1.416 – 0.380 | -1.569 – 0.421 | 0.245 | 22.741 |
| Age | 0.027 | 0.171 | 0.030 | 0.192 | -0.327 – 0.381 | -0.367 – 0.428 | 0.876 | 23.088 |
| Age of diagnosis | 0.009 | 0.051 | 0.034 | 0.193 | -0.096 – 0.114 | -0.365 – 0.433 | 0.863 | 23.064 |
| SES | 0.078 | 0.172 | 0.083 | 0.182 | -0.277 – 0.433 | -0.294 – 0.459 | 0.654 | 23.227 |
| Other health conditions | -0.214 | 0.408 | -0.238 | 0.452 | -1.057 – 0.628 | -1.172 – 0.697 | 0.604 | 23.139 |
| Time X Condition | -0.355 | 0.400 | -0.393 | 0.444 | -1.181 – 0.471 | -1.309 – 0.522 | 0.384 | 24.241 |
| Time X Prior Camping | 0.631 | 0.503 | 0.699 | 0.557 | -0.404 – 1.665 | -0.448 – 1.846 | 0.221 | 25.215 |
| Condition X Prior Camping | 1.157 | 0.903 | 1.283 | 1.001 | -0.683 – 2.997 | -0.757 – 3.322 | 0.209 | 31.484 |
| Time X Condition X Prior Camping | -0.105 | 0.611 | -0.116 | 0.677 | -1.363 – 1.154 | -1.511 – 1.279 | 0.865 | 24.848 |
| **Random Effects** | | | | | | | | |
| σ^2^ | 0.19 | | | | | | | |
| τ_00_ _ID_Matching_ | 0.68 | | | | | | | |
| ICC | 0.78 | | | | | | | |
| N _ID_Matching_ | 32 | | | | | | | |
| Observations | 60 | | | | | | | |
| Marginal R^2^ / Conditional R^2^ | 0.138 / 0.809 | | | | | | | |

**Table S13.3**

*Results of Linear Mixed Model Predicting IIQ: Rejection from Prior Camping, Condition, and Time, Controlling for Covariates*

|  | **IIQ: Rejection** | | | | | | | |
| --- | --- | --- | --- | --- | --- | --- | --- | --- |
| *Predictors* | *B* | *SE* | *β* | *Std. SE* | *CI* | *Std. CI* | *p* | *df* |
| (Intercept) | 3.265 | 2.421 | 0.093 | 0.539 | -1.744 – 8.273 | -1.018 – 1.203 | 0.191 | 25.120 |
| Time | -0.120 | 0.222 | -0.148 | 0.273 | -0.578 – 0.337 | -0.711 – 0.415 | 0.592 | 24.600 |
| Condition | 0.196 | 0.518 | 0.241 | 0.638 | -0.867 – 1.258 | -1.067 – 1.549 | 0.709 | 27.819 |
| Prior Camping | 0.217 | 0.607 | 0.267 | 0.747 | -1.016 – 1.449 | -1.250 – 1.784 | 0.723 | 34.256 |
| Gender (1 = female) | -0.020 | 0.369 | -0.024 | 0.455 | -0.784 – 0.744 | -0.965 – 0.916 | 0.958 | 22.724 |
| Age | -0.078 | 0.146 | -0.097 | 0.182 | -0.380 – 0.223 | -0.473 – 0.279 | 0.597 | 23.091 |
| Age of diagnosis | -0.046 | 0.043 | -0.195 | 0.182 | -0.136 – 0.043 | -0.573 – 0.182 | 0.295 | 23.066 |
| SES | 0.039 | 0.146 | 0.045 | 0.172 | -0.264 – 0.341 | -0.311 – 0.402 | 0.794 | 23.237 |
| Other health conditions | 0.087 | 0.347 | 0.108 | 0.427 | -0.630 – 0.805 | -0.776 – 0.991 | 0.803 | 23.145 |
| Time X Condition | 1.120 | 0.352 | 1.379 | 0.433 | 0.395 – 1.846 | 0.486 – 2.272 | **0.004** | 24.256 |
| Time X Prior Camping | 0.409 | 0.441 | 0.504 | 0.543 | -0.499 – 1.318 | -0.615 – 1.622 | 0.363 | 25.286 |
| Condition X Prior Camping | -0.732 | 0.773 | -0.901 | 0.951 | -2.306 – 0.842 | -2.839 – 1.036 | 0.350 | 31.942 |
| Time X Condition X Prior Camping | -1.418 | 0.536 | -1.746 | 0.660 | -2.523 – -0.313 | -3.106 – -0.385 | **0.014** | 24.898 |
| **Random Effects** | | | | | | | | |
| σ^2^ | 0.15 | | | | | | | |
| τ_00_ _ID_Matching_ | 0.49 | | | | | | | |
| ICC | 0.77 | | | | | | | |
| N _ID_Matching_ | 32 | | | | | | | |
| Observations | 60 | | | | | | | |
| Marginal R^2^ / Conditional R^2^ | 0.236 / 0.821 | | | | | | | |

**Table S13.4**

*Results of Linear Mixed Model Predicting IIQ: Acceptance from Prior Camping, Condition, and Time, Controlling for Covariates*

|  | **IIQ: Acceptance** | | | | | | | |
| --- | --- | --- | --- | --- | --- | --- | --- | --- |
| *Predictors* | *B* | *SE* | *β* | *Std. SE* | *CI* | *Std. CI* | *p* | *df* |
| (Intercept) | 3.730 | 2.579 | 0.055 | 0.621 | -1.604 – 9.063 | -1.224 – 1.335 | 0.162 | 24.818 |
| Time | -0.331 | 0.219 | -0.442 | 0.293 | -0.783 – 0.121 | -1.046 – 0.162 | 0.144 | 24.521 |
| Condition | 0.356 | 0.549 | 0.475 | 0.732 | -0.770 – 1.481 | -1.028 – 1.977 | 0.522 | 27.145 |
| Prior Camping | 0.016 | 0.636 | 0.021 | 0.850 | -1.279 – 1.310 | -1.708 – 1.750 | 0.981 | 33.004 |
| Gender (1 = female) | -0.224 | 0.393 | -0.299 | 0.525 | -1.039 – 0.590 | -1.387 – 0.788 | 0.574 | 22.762 |
| Age | -0.012 | 0.155 | -0.017 | 0.210 | -0.333 – 0.308 | -0.451 – 0.417 | 0.937 | 23.084 |
| Age of diagnosis | -0.001 | 0.046 | -0.005 | 0.211 | -0.096 – 0.094 | -0.441 – 0.430 | 0.979 | 23.061 |
| SES | 0.115 | 0.156 | 0.147 | 0.199 | -0.207 – 0.437 | -0.265 – 0.558 | 0.468 | 23.213 |
| Other health conditions | 0.036 | 0.370 | 0.049 | 0.493 | -0.728 – 0.801 | -0.972 – 1.069 | 0.922 | 23.131 |
| Time X Condition | 0.231 | 0.348 | 0.308 | 0.464 | -0.486 – 0.948 | -0.649 – 1.266 | 0.513 | 24.221 |
| Time X Prior Camping | 0.202 | 0.437 | 0.270 | 0.583 | -0.697 – 1.101 | -0.930 – 1.470 | 0.647 | 25.121 |
| Condition X Prior Camping | -0.245 | 0.813 | -0.327 | 1.086 | -1.903 – 1.413 | -2.542 – 1.887 | 0.765 | 30.874 |
| Time X Condition X Prior Camping | 0.005 | 0.530 | 0.007 | 0.708 | -1.088 – 1.098 | -1.453 – 1.466 | 0.992 | 24.783 |
| **Random Effects** | | | | | | | | |
| σ^2^ | 0.15 | | | | | | | |
| τ_00_ _ID_Matching_ | 0.56 | | | | | | | |
| ICC | 0.80 | | | | | | | |
| N _ID_Matching_ | 32 | | | | | | | |
| Observations | 60 | | | | | | | |
| Marginal R^2^ / Conditional R^2^ | 0.074 / 0.810 | | | | | | | |

**Table S13.5**

*Results of Linear Mixed Model Predicting IIQ: Engulfment from Prior Camping, Condition, and Time, Controlling for Covariates*

|  | **IIQ: Engulfment** | | | | | | | |
| --- | --- | --- | --- | --- | --- | --- | --- | --- |
| *Predictors* | *B* | *SE* | *β* | *Std. SE* | *CI* | *Std. CI* | *p* | *df* |
| (Intercept) | -1.270 | 2.476 | -0.580 | 0.559 | -6.391 – 3.851 | -1.729 – 0.569 | 0.613 | 25.584 |
| Time | 0.066 | 0.250 | 0.081 | 0.310 | -0.449 – 0.580 | -0.557 – 0.720 | 0.795 | 24.717 |
| Condition | -0.039 | 0.535 | -0.049 | 0.665 | -1.135 – 1.056 | -1.408 – 1.311 | 0.942 | 28.841 |
| Prior Camping | 0.230 | 0.634 | 0.286 | 0.787 | -1.056 – 1.516 | -1.310 – 1.881 | 0.719 | 35.965 |
| Gender (1 = female) | 0.520 | 0.377 | 0.646 | 0.468 | -0.261 – 1.301 | -0.324 – 1.615 | 0.181 | 22.667 |
| Age | 0.188 | 0.149 | 0.236 | 0.187 | -0.120 – 0.496 | -0.151 – 0.624 | 0.220 | 23.097 |
| Age of diagnosis | 0.034 | 0.044 | 0.145 | 0.188 | -0.057 – 0.126 | -0.244 – 0.534 | 0.448 | 23.072 |
| SES | -0.030 | 0.150 | -0.035 | 0.178 | -0.339 – 0.280 | -0.403 – 0.332 | 0.844 | 23.267 |
| Other health conditions | 0.140 | 0.355 | 0.174 | 0.441 | -0.594 – 0.874 | -0.737 – 1.085 | 0.697 | 23.163 |
| Time X Condition | 0.372 | 0.396 | 0.462 | 0.491 | -0.445 – 1.188 | -0.552 – 1.475 | 0.357 | 24.310 |
| Time X Prior Camping | 0.153 | 0.496 | 0.189 | 0.616 | -0.868 – 1.173 | -1.077 – 1.456 | 0.761 | 25.529 |
| Condition X Prior Camping | -0.256 | 0.804 | -0.318 | 0.998 | -1.891 – 1.379 | -2.347 – 1.712 | 0.752 | 33.452 |
| Time X Condition X Prior Camping | -0.548 | 0.603 | -0.680 | 0.749 | -1.790 – 0.694 | -2.222 – 0.862 | 0.372 | 25.067 |
| **Random Effects** | | | | | | | | |
| σ^2^ | 0.19 | | | | | | | |
| τ_00_ _ID_Matching_ | 0.49 | | | | | | | |
| ICC | 0.72 | | | | | | | |
| N _ID_Matching_ | 32 | | | | | | | |
| Observations | 60 | | | | | | | |
| Marginal R^2^ / Conditional R^2^ | 0.166 / 0.768 | | | | | | | |

**Table S13.6**

*Results of Linear Mixed Model Predicting IIQ: Enrichment from Prior Camping, Condition, and Time, Controlling for Covariates*

|  | **IIQ: Enrichment** | | | | | | | |
| --- | --- | --- | --- | --- | --- | --- | --- | --- |
| *Predictors* | *B* | *SE* | *β* | *Std. SE* | *CI* | *Std. CI* | *p* | *df* |
| (Intercept) | -3.371 | 2.257 | 0.218 | 0.453 | -8.039 – 1.298 | -0.708 – 1.144 | 0.149 | 28.899 |
| Time | -0.058 | 0.338 | -0.062 | 0.361 | -0.752 – 0.637 | -0.805 – 0.682 | 0.866 | 25.433 |
| Condition | 0.569 | 0.521 | 0.609 | 0.557 | -0.488 – 1.626 | -0.522 – 1.740 | 0.282 | 35.560 |
| Prior Camping | 0.368 | 0.653 | 0.393 | 0.699 | -0.949 – 1.685 | -1.016 – 1.802 | 0.576 | 42.919 |
| Gender (1 = female) | -0.173 | 0.342 | -0.185 | 0.366 | -0.882 – 0.536 | -0.944 – 0.573 | 0.618 | 22.294 |
| Age | 0.161 | 0.136 | 0.175 | 0.147 | -0.120 – 0.442 | -0.130 – 0.479 | 0.248 | 23.056 |
| Age of diagnosis | 0.078 | 0.040 | 0.287 | 0.148 | -0.005 – 0.162 | -0.018 – 0.593 | 0.064 | 23.046 |
| SES | 0.533 | 0.137 | 0.545 | 0.140 | 0.250 – 0.816 | 0.256 – 0.834 | **0.001** | 23.344 |
| Other health conditions | 0.228 | 0.324 | 0.244 | 0.347 | -0.441 – 0.898 | -0.472 – 0.961 | 0.488 | 23.199 |
| Time X Condition | -0.942 | 0.538 | -1.008 | 0.575 | -2.050 – 0.166 | -2.193 – 0.177 | 0.092 | 24.671 |
| Time X Prior Camping | -0.431 | 0.666 | -0.461 | 0.712 | -1.798 – 0.935 | -1.923 – 1.000 | 0.523 | 26.956 |
| Condition X Prior Camping | -1.182 | 0.813 | -1.265 | 0.870 | -2.824 – 0.460 | -3.021 – 0.492 | 0.154 | 40.658 |
| Time X Condition X Prior Camping | 1.485 | 0.813 | 1.589 | 0.870 | -0.186 – 3.157 | -0.199 – 3.377 | 0.079 | 26.064 |
| **Random Effects** | | | | | | | | |
| σ^2^ | 0.35 | | | | | | | |
| τ_00_ _ID_Matching_ | 0.30 | | | | | | | |
| ICC | 0.46 | | | | | | | |
| N _ID_Matching_ | 32 | | | | | | | |
| Observations | 60 | | | | | | | |
| Marginal R^2^ / Conditional R^2^ | 0.363 / 0.656 | | | | | | | |

**Table S13.7**

*Results of Linear Mixed Model Predicting DIFI (Overlap) from Prior Camping, Condition, and Time, Controlling for Covariates*

|  | **DIFI (Overlap)** | | | | | | | |
| --- | --- | --- | --- | --- | --- | --- | --- | --- |
| *Predictors* | *B* | *SE* | *β* | *Std. SE* | *CI* | *Std. CI* | *p* | *df* |
| (Intercept) | 79.585 | 31.085 | -0.236 | 0.232 | 17.879 – 141.292 | -0.694 – 0.223 | **0.012** | 117.379 |
| Time | -3.038 | 6.686 | -0.073 | 0.161 | -16.325 – 10.248 | -0.393 – 0.247 | 0.651 | 88.121 |
| Condition | 30.469 | 11.894 | 0.733 | 0.286 | 6.965 – 53.973 | 0.168 – 1.298 | **0.011** | 147.691 |
| Prior Camping | -14.416 | 17.827 | -0.347 | 0.429 | -49.649 – 20.817 | -1.194 – 0.501 | 0.420 | 145.643 |
| Gender (1 = female) | -3.347 | 8.177 | -0.081 | 0.197 | -19.586 – 12.893 | -0.471 – 0.310 | 0.683 | 92.275 |
| Age | -2.846 | 1.925 | -0.144 | 0.097 | -6.668 – 0.977 | -0.337 – 0.049 | 0.143 | 92.133 |
| Age of diagnosis | -1.308 | 1.237 | -0.094 | 0.089 | -3.765 – 1.149 | -0.271 – 0.083 | 0.293 | 92.753 |
| SES | -1.942 | 3.037 | -0.057 | 0.089 | -7.973 – 4.088 | -0.234 – 0.120 | 0.524 | 92.880 |
| Other health conditions | -5.135 | 8.942 | -0.124 | 0.215 | -22.887 – 12.618 | -0.551 – 0.304 | 0.567 | 95.080 |
| Time X Condition | -15.917 | 11.114 | -0.383 | 0.267 | -37.993 – 6.158 | -0.914 – 0.148 | 0.156 | 91.377 |
| Time X Prior Camping | 35.835 | 16.533 | 0.862 | 0.398 | 2.988 – 68.682 | 0.072 – 1.652 | **0.033** | 89.482 |
| Condition X Prior Camping | 9.163 | 21.311 | 0.220 | 0.513 | -32.951 – 51.277 | -0.793 – 1.234 | 0.668 | 147.379 |
| Time X Condition X Prior Camping | -15.963 | 19.779 | -0.384 | 0.476 | -55.255 – 23.329 | -1.329 – 0.561 | 0.422 | 90.266 |
| **Random Effects** | | | | | | | | |
| σ^2^ | 705.03 | | | | | | | |
| τ_00_ _ID_Matching_ | 945.59 | | | | | | | |
| ICC | 0.57 | | | | | | | |
| N _ID_Matching_ | 102 | | | | | | | |
| Observations | 191 | | | | | | | |
| Marginal R^2^ / Conditional R^2^ | 0.118 / 0.623 | | | | | | | |

**Table S13.8**

*Results of Linear Mixed Model Predicting* GFD-PCS *from Prior Camping, Condition, and Time, Controlling for Covariates*

|  | GFD-PCS | | | | | | | |
| --- | --- | --- | --- | --- | --- | --- | --- | --- |
| *Predictors* | *B* | *SE* | *β* | *Std. SE* | *CI* | *Std. CI* | *p* | *df* |
| (Intercept) | 5.037 | 0.265 | 0.091 | 0.238 | 4.511 – 5.563 | -0.381 – 0.564 | **<0.001** | 118.895 |
| Time | -0.003 | 0.062 | -0.010 | 0.177 | -0.126 – 0.119 | -0.362 – 0.342 | 0.956 | 89.498 |
| Condition | -0.027 | 0.102 | -0.079 | 0.294 | -0.230 – 0.175 | -0.660 – 0.502 | 0.789 | 147.658 |
| Prior Camping | -0.194 | 0.155 | -0.556 | 0.446 | -0.500 – 0.113 | -1.436 – 0.324 | 0.214 | 152.260 |
| Gender (1 = female) | -0.090 | 0.070 | -0.258 | 0.200 | -0.229 – 0.049 | -0.656 – 0.140 | 0.200 | 90.616 |
| Age | -0.019 | 0.016 | -0.114 | 0.099 | -0.052 – 0.014 | -0.311 – 0.083 | 0.253 | 90.956 |
| Age of diagnosis | -0.006 | 0.011 | -0.055 | 0.091 | -0.027 – 0.015 | -0.235 – 0.125 | 0.545 | 90.825 |
| SES | 0.005 | 0.026 | 0.019 | 0.090 | -0.046 – 0.057 | -0.160 – 0.198 | 0.834 | 92.200 |
| Other health conditions | 0.059 | 0.077 | 0.170 | 0.220 | -0.093 – 0.211 | -0.267 – 0.607 | 0.443 | 92.258 |
| Time X Condition | 0.027 | 0.101 | 0.076 | 0.290 | -0.174 – 0.227 | -0.499 – 0.652 | 0.793 | 90.327 |
| Time X Prior Camping | 0.208 | 0.154 | 0.598 | 0.443 | -0.098 – 0.515 | -0.281 – 1.477 | 0.180 | 91.965 |
| Condition X Prior Camping | 0.276 | 0.185 | 0.793 | 0.532 | -0.090 – 0.643 | -0.258 – 1.844 | 0.138 | 152.816 |
| Time X Condition X Prior Camping | -0.215 | 0.183 | -0.616 | 0.527 | -0.579 – 0.150 | -1.662 – 0.430 | 0.245 | 91.755 |
| **Random Effects** | | | | | | | | |
| σ^2^ | 0.06 | | | | | | | |
| τ_00_ _ID_Matching_ | 0.06 | | | | | | | |
| ICC | 0.50 | | | | | | | |
| N _ID_Matching_ | 101 | | | | | | | |
| Observations | 192 | | | | | | | |
| Marginal R^2^ / Conditional R^2^ | 0.048 / 0.526 | | | | | | | |

**Table S13.9**

*Results of Linear Mixed Model Predicting* CDAT *from Prior Camping, Condition, and Time, Controlling for Covariates*

|  | **CDAT** | | | | | | | |
| --- | --- | --- | --- | --- | --- | --- | --- | --- |
| *Predictors* | *B* | *SE* | *β* | *Std. SE* | *CI* | *Std. CI* | *p* | *df* |
| (Intercept) | 1.054 | 0.338 | 0.044 | 0.225 | 0.383 – 1.725 | -0.401 – 0.489 | **0.002** | 112.208 |
| Time | 0.037 | 0.067 | 0.080 | 0.145 | -0.097 – 0.171 | -0.208 – 0.368 | 0.584 | 89.902 |
| Condition | -0.118 | 0.126 | -0.255 | 0.272 | -0.368 – 0.131 | -0.793 – 0.283 | 0.350 | 136.172 |
| Prior Camping | 0.139 | 0.191 | 0.299 | 0.411 | -0.238 – 0.516 | -0.513 – 1.111 | 0.468 | 141.000 |
| Gender (1 = female) | -0.055 | 0.089 | -0.118 | 0.192 | -0.232 – 0.123 | -0.501 – 0.264 | 0.540 | 91.217 |
| Age | 0.074 | 0.021 | 0.334 | 0.095 | 0.032 – 0.116 | 0.145 – 0.523 | **0.001** | 91.400 |
| Age of diagnosis | 0.019 | 0.013 | 0.123 | 0.087 | -0.008 – 0.046 | -0.050 – 0.296 | 0.160 | 91.394 |
| SES | -0.051 | 0.033 | -0.133 | 0.086 | -0.116 – 0.015 | -0.305 – 0.038 | 0.127 | 92.505 |
| Other health conditions | 0.034 | 0.098 | 0.073 | 0.211 | -0.161 – 0.229 | -0.347 – 0.492 | 0.731 | 92.638 |
| Time X Condition | 0.045 | 0.110 | 0.096 | 0.237 | -0.174 – 0.263 | -0.375 – 0.567 | 0.686 | 90.586 |
| Time X Prior Camping | -0.090 | 0.168 | -0.193 | 0.363 | -0.424 – 0.245 | -0.913 – 0.527 | 0.596 | 91.905 |
| Condition X Prior Camping | 0.053 | 0.228 | 0.113 | 0.490 | -0.397 – 0.503 | -0.855 – 1.082 | 0.817 | 141.377 |
| Time X Condition X Prior Camping | -0.063 | 0.200 | -0.136 | 0.431 | -0.461 – 0.334 | -0.992 – 0.720 | 0.753 | 91.661 |
| **Random Effects** | | | | | | | | |
| σ^2^ | 0.07 | | | | | | | |
| τ_00_ _ID_Matching_ | 0.12 | | | | | | | |
| ICC | 0.62 | | | | | | | |
| N _ID_Matching_ | 101 | | | | | | | |
| Observations | 193 | | | | | | | |
| Marginal R^2^ / Conditional R^2^ | 0.169 / 0.681 | | | | | | | |

**Table S13.10**

*Results of Linear Mixed Model Predicting CDPQOL: Negative emotions (Ages 8-12 years) from Prior Camping, Condition, and Time, Controlling for Covariates*

|  | **CDPQOL: Negative emotions (Ages 8-12 years)** | | | | | | | |
| --- | --- | --- | --- | --- | --- | --- | --- | --- |
| *Predictors* | *B* | *SE* | *β* | *Std. SE* | *CI* | *Std. CI* | *p* | *df* |
| (Intercept) | 1.261 | 0.668 | -0.028 | 0.295 | -0.075 – 2.596 | -0.615 – 0.560 | 0.064 | 74.397 |
| Time | 0.100 | 0.115 | 0.161 | 0.185 | -0.130 – 0.329 | -0.209 – 0.531 | 0.389 | 61.449 |
| Condition | -0.055 | 0.216 | -0.088 | 0.348 | -0.484 – 0.375 | -0.780 – 0.604 | 0.801 | 90.906 |
| Prior Camping | 0.375 | 0.344 | 0.605 | 0.555 | -0.309 – 1.060 | -0.497 – 1.707 | 0.279 | 88.675 |
| Gender (1 = female) | -0.051 | 0.165 | -0.082 | 0.266 | -0.382 – 0.280 | -0.615 – 0.450 | 0.758 | 60.796 |
| Age | 0.062 | 0.061 | 0.127 | 0.126 | -0.061 – 0.184 | -0.125 – 0.379 | 0.316 | 60.635 |
| Age of diagnosis | 0.011 | 0.025 | 0.049 | 0.111 | -0.039 – 0.062 | -0.173 – 0.271 | 0.658 | 61.066 |
| SES | -0.012 | 0.056 | -0.026 | 0.119 | -0.125 – 0.100 | -0.263 – 0.211 | 0.828 | 60.763 |
| Other health conditions | 0.114 | 0.177 | 0.184 | 0.285 | -0.240 – 0.469 | -0.386 – 0.755 | 0.520 | 59.962 |
| Time X Condition | -0.141 | 0.188 | -0.226 | 0.302 | -0.515 – 0.234 | -0.830 – 0.378 | 0.457 | 61.542 |
| Time X Prior Camping | 0.055 | 0.312 | 0.089 | 0.503 | -0.569 – 0.679 | -0.917 – 1.094 | 0.861 | 59.537 |
| Condition X Prior Camping | -0.439 | 0.410 | -0.707 | 0.660 | -1.253 – 0.374 | -2.018 – 0.603 | 0.287 | 90.297 |
| Time X Condition X Prior Camping | 0.157 | 0.370 | 0.252 | 0.596 | -0.583 – 0.896 | -0.940 – 1.444 | 0.674 | 60.063 |
| **Random Effects** | | | | | | | | |
| σ^2^ | 0.17 | | | | | | | |
| τ_00_ _ID_Matching_ | 0.24 | | | | | | | |
| ICC | 0.58 | | | | | | | |
| N _ID_Matching_ | 70 | | | | | | | |
| Observations | 133 | | | | | | | |
| Marginal R^2^ / Conditional R^2^ | 0.056 / 0.608 | | | | | | | |

**Table S13.11**

*Results of Linear Mixed Model Predicting CDPQOL: School (Ages 8-12 years) from Prior Camping, Condition, and Time, Controlling for Covariates*

|  | **CDPQOL: School (Ages 8-12 years)** | | | | | | | |
| --- | --- | --- | --- | --- | --- | --- | --- | --- |
| *Predictors* | *B* | *SE* | *β* | *Std. SE* | *CI* | *Std. CI* | *p* | *df* |
| (Intercept) | 1.167 | 0.645 | 0.207 | 0.299 | -0.122 – 2.456 | -0.389 – 0.802 | 0.075 | 76.166 |
| Time | -0.066 | 0.105 | -0.111 | 0.177 | -0.277 – 0.145 | -0.465 – 0.242 | 0.532 | 59.841 |
| Condition | -0.154 | 0.211 | -0.259 | 0.353 | -0.573 – 0.264 | -0.960 – 0.442 | 0.465 | 91.676 |
| Prior Camping | 0.189 | 0.329 | 0.317 | 0.550 | -0.464 – 0.843 | -0.777 – 1.411 | 0.566 | 85.030 |
| Gender (1 = female) | -0.117 | 0.161 | -0.195 | 0.269 | -0.438 – 0.205 | -0.733 – 0.343 | 0.471 | 61.612 |
| Age | 0.041 | 0.059 | 0.088 | 0.127 | -0.077 – 0.159 | -0.166 – 0.342 | 0.492 | 60.242 |
| Age of diagnosis | 0.029 | 0.024 | 0.135 | 0.112 | -0.019 – 0.078 | -0.088 – 0.359 | 0.231 | 60.936 |
| SES | 0.000 | 0.054 | 0.001 | 0.119 | -0.108 – 0.109 | -0.238 – 0.239 | 0.996 | 60.440 |
| Other health conditions | 0.039 | 0.172 | 0.065 | 0.288 | -0.305 – 0.383 | -0.511 – 0.641 | 0.822 | 60.697 |
| Time X Condition | 0.006 | 0.174 | 0.010 | 0.291 | -0.341 – 0.353 | -0.572 – 0.592 | 0.972 | 60.056 |
| Time X Prior Camping | 0.441 | 0.282 | 0.739 | 0.472 | -0.123 – 1.006 | -0.206 – 1.685 | 0.123 | 57.598 |
| Condition X Prior Camping | -0.203 | 0.392 | -0.340 | 0.657 | -0.983 – 0.577 | -1.646 – 0.966 | 0.606 | 87.603 |
| Time X Condition X Prior Camping | -0.242 | 0.336 | -0.405 | 0.562 | -0.914 – 0.430 | -1.531 – 0.720 | 0.474 | 58.171 |
| **Random Effects** | | | | | | | | |
| σ^2^ | 0.14 | | | | | | | |
| τ_00_ _ID_Matching_ | 0.23 | | | | | | | |
| ICC | 0.63 | | | | | | | |
| N _ID_Matching_ | 70 | | | | | | | |
| Observations | 131 | | | | | | | |
| Marginal R^2^ / Conditional R^2^ | 0.067 / 0.654 | | | | | | | |

**Table S13.12**

*Results of Linear Mixed Model Predicting CDPQOL: Enjoyment (Ages 8-12 years) from Prior Camping, Condition, and Time, Controlling for Covariates*

|  | **CDPQOL: Enjoyment (Ages 8-12 years)** | | | | | | | |
| --- | --- | --- | --- | --- | --- | --- | --- | --- |
| *Predictors* | *B* | *SE* | *β* | *Std. SE* | *CI* | *Std. CI* | *p* | *df* |
| (Intercept) | 1.151 | 0.589 | -0.208 | 0.283 | -0.027 – 2.328 | -0.771 – 0.355 | 0.055 | 77.379 |
| Time | 0.014 | 0.113 | 0.023 | 0.195 | -0.212 – 0.239 | -0.367 – 0.414 | 0.905 | 60.884 |
| Condition | 0.305 | 0.199 | 0.528 | 0.345 | -0.090 – 0.700 | -0.156 – 1.213 | 0.129 | 98.647 |
| Prior Camping | 0.856 | 0.310 | 1.483 | 0.538 | 0.240 – 1.472 | 0.415 – 2.550 | **0.007** | 93.850 |
| Gender (1 = female) | 0.048 | 0.146 | 0.084 | 0.253 | -0.244 – 0.341 | -0.422 – 0.590 | 0.742 | 60.918 |
| Age | -0.036 | 0.054 | -0.080 | 0.119 | -0.144 – 0.072 | -0.319 – 0.159 | 0.506 | 60.083 |
| Age of diagnosis | 0.031 | 0.022 | 0.149 | 0.105 | -0.013 – 0.076 | -0.062 – 0.359 | 0.164 | 60.576 |
| SES | 0.042 | 0.050 | 0.096 | 0.112 | -0.057 – 0.142 | -0.128 – 0.320 | 0.395 | 60.497 |
| Other health conditions | 0.017 | 0.157 | 0.029 | 0.272 | -0.297 – 0.330 | -0.514 – 0.572 | 0.914 | 60.579 |
| Time X Condition | -0.304 | 0.187 | -0.526 | 0.325 | -0.678 – 0.071 | -1.175 – 0.122 | 0.110 | 61.791 |
| Time X Prior Camping | -0.514 | 0.307 | -0.890 | 0.531 | -1.127 – 0.100 | -1.953 – 0.173 | 0.099 | 58.722 |
| Condition X Prior Camping | -1.126 | 0.372 | -1.950 | 0.644 | -1.863 – -0.388 | -3.228 – -0.672 | **0.003** | 96.304 |
| Time X Condition X Prior Camping | 0.816 | 0.365 | 1.413 | 0.632 | 0.085 – 1.546 | 0.148 – 2.678 | **0.029** | 59.523 |
| **Random Effects** | | | | | | | | |
| σ^2^ | 0.16 | | | | | | | |
| τ_00_ _ID_Matching_ | 0.17 | | | | | | | |
| ICC | 0.51 | | | | | | | |
| N _ID_Matching_ | 70 | | | | | | | |
| Observations | 132 | | | | | | | |
| Marginal R^2^ / Conditional R^2^ | 0.107 / 0.559 | | | | | | | |

**Table S13.13**

*Results of Linear Mixed Model Predicting CDPQOL: Social (Ages 13-16 years) from Prior Camping, Condition, and Time, Controlling for Covariates*

|  | **CDPQOL: Social (Ages 13-16 years)** | | | | | | | |
| --- | --- | --- | --- | --- | --- | --- | --- | --- |
| *Predictors* | *B* | *SE* | *β* | *Std. SE* | *CI* | *Std. CI* | *p* | *df* |
| (Intercept) | 2.504 | 2.597 | -0.534 | 0.558 | -2.869 – 7.876 | -1.685 – 0.618 | 0.345 | 24.426 |
| Time | 0.118 | 0.196 | 0.141 | 0.235 | -0.287 – 0.522 | -0.344 – 0.625 | 0.555 | 24.416 |
| Condition | -0.206 | 0.548 | -0.246 | 0.655 | -1.331 – 0.920 | -1.593 – 1.101 | 0.710 | 26.263 |
| Prior Camping | 1.093 | 0.628 | 1.308 | 0.751 | -0.187 – 2.373 | -0.224 – 2.840 | 0.092 | 31.209 |
| Gender (1 = female) | 0.532 | 0.397 | 0.636 | 0.475 | -0.289 – 1.352 | -0.346 – 1.618 | 0.193 | 22.812 |
| Age | -0.047 | 0.156 | -0.057 | 0.190 | -0.371 – 0.276 | -0.449 – 0.335 | 0.765 | 23.073 |
| Age of diagnosis | 0.022 | 0.046 | 0.090 | 0.190 | -0.074 – 0.118 | -0.303 – 0.484 | 0.640 | 23.052 |
| SES | -0.056 | 0.157 | -0.064 | 0.179 | -0.380 – 0.269 | -0.435 – 0.307 | 0.725 | 23.178 |
| Other health conditions | -0.052 | 0.372 | -0.062 | 0.445 | -0.821 – 0.718 | -0.983 – 0.859 | 0.891 | 23.109 |
| Time X Condition | -0.082 | 0.311 | -0.098 | 0.372 | -0.723 – 0.559 | -0.865 – 0.670 | 0.795 | 24.174 |
| Time X Prior Camping | 0.011 | 0.391 | 0.013 | 0.468 | -0.794 – 0.817 | -0.951 – 0.977 | 0.977 | 24.899 |
| Condition X Prior Camping | -0.836 | 0.805 | -1.000 | 0.964 | -2.482 – 0.810 | -2.971 – 0.970 | 0.308 | 29.388 |
| Time X Condition X Prior Camping | -0.237 | 0.475 | -0.284 | 0.568 | -1.216 – 0.741 | -1.455 – 0.887 | 0.621 | 24.628 |
| **Random Effects** | | | | | | | | |
| σ^2^ | 0.12 | | | | | | | |
| τ_00_ _ID_Matching_ | 0.59 | | | | | | | |
| ICC | 0.84 | | | | | | | |
| N _ID_Matching_ | 32 | | | | | | | |
| Observations | 60 | | | | | | | |
| Marginal R^2^ / Conditional R^2^ | 0.180 / 0.865 | | | | | | | |

**Table S13.14**

*Results of Linear Mixed Model Predicting CDPQOL: Uncertainty (Ages 13-16 years) from Prior Camping, Condition, and Time, Controlling for Covariates*

|  | **CDPQOL: Uncertainty (Ages 13-16 years)** | | | | | | | |
| --- | --- | --- | --- | --- | --- | --- | --- | --- |
| *Predictors* | *B* | *SE* | *β* | *Std. SE* | *CI* | *Std. CI* | *p* | *df* |
| (Intercept) | -1.141 | 2.541 | -0.881 | 0.571 | -6.397 – 4.116 | -2.058 – 0.296 | 0.658 | 24.060 |
| Time | 0.108 | 0.166 | 0.136 | 0.208 | -0.234 – 0.451 | -0.293 – 0.565 | 0.520 | 24.315 |
| Condition | 0.426 | 0.531 | 0.534 | 0.666 | -0.668 – 1.519 | -0.838 – 1.905 | 0.431 | 25.433 |
| Prior Camping | 1.274 | 0.602 | 1.598 | 0.755 | 0.044 – 2.505 | 0.055 – 3.141 | **0.043** | 29.351 |
| Gender (1 = female) | 0.586 | 0.388 | 0.735 | 0.487 | -0.217 – 1.390 | -0.272 – 1.743 | 0.145 | 22.859 |
| Age | 0.125 | 0.153 | 0.159 | 0.194 | -0.191 – 0.441 | -0.243 – 0.561 | 0.422 | 23.060 |
| Age of diagnosis | 0.023 | 0.045 | 0.101 | 0.195 | -0.070 – 0.117 | -0.302 – 0.504 | 0.610 | 23.042 |
| SES | 0.065 | 0.153 | 0.078 | 0.184 | -0.252 – 0.383 | -0.302 – 0.459 | 0.675 | 23.141 |
| Other health conditions | -0.313 | 0.364 | -0.392 | 0.456 | -1.066 – 0.440 | -1.336 – 0.552 | 0.399 | 23.086 |
| Time X Condition | -0.358 | 0.263 | -0.450 | 0.330 | -0.901 – 0.184 | -1.130 – 0.231 | 0.185 | 24.130 |
| Time X Prior Camping | -0.830 | 0.331 | -1.041 | 0.415 | -1.513 – -0.147 | -1.897 – -0.185 | **0.019** | 24.682 |
| Condition X Prior Camping | -1.213 | 0.775 | -1.521 | 0.972 | -2.802 – 0.376 | -3.513 – 0.471 | 0.129 | 27.894 |
| Time X Condition X Prior Camping | 0.788 | 0.402 | 0.988 | 0.504 | -0.041 – 1.617 | -0.051 – 2.028 | 0.061 | 24.477 |
| **Random Effects** | | | | | | | | |
| σ^2^ | 0.08 | | | | | | | |
| τ_00_ _ID_Matching_ | 0.58 | | | | | | | |
| ICC | 0.87 | | | | | | | |
| N _ID_Matching_ | 32 | | | | | | | |
| Observations | 60 | | | | | | | |
| Marginal R^2^ / Conditional R^2^ | 0.153 / 0.894 | | | | | | | |

**Table S13.15**

*Results of Linear Mixed Model Predicting CDPQOL: Limitations (Ages 13-16 years) from Prior Camping, Condition, and Time, Controlling for Covariates*

|  | **CDPQOL: Limitations (Ages 13-16 years)** | | | | | | | |
| --- | --- | --- | --- | --- | --- | --- | --- | --- |
| *Predictors* | *B* | *SE* | *β* | *Std. SE* | *CI* | *Std. CI* | *p* | *df* |
| (Intercept) | 1.943 | 3.001 | -0.754 | 0.543 | -4.264 – 8.150 | -1.874 – 0.365 | 0.524 | 24.309 |
| Time | -0.039 | 0.217 | -0.040 | 0.219 | -0.488 – 0.409 | -0.492 – 0.412 | 0.858 | 24.384 |
| Condition | 0.038 | 0.631 | 0.038 | 0.636 | -1.259 – 1.335 | -1.270 – 1.346 | 0.952 | 25.999 |
| Prior Camping | 1.308 | 0.721 | 1.319 | 0.727 | -0.162 – 2.779 | -0.164 – 2.802 | 0.079 | 30.635 |
| Gender (1 = female) | 0.766 | 0.458 | 0.772 | 0.462 | -0.182 – 1.714 | -0.184 – 1.728 | 0.108 | 22.827 |
| Age | -0.005 | 0.181 | -0.005 | 0.184 | -0.378 – 0.369 | -0.386 – 0.377 | 0.979 | 23.069 |
| Age of diagnosis | -0.015 | 0.054 | -0.052 | 0.185 | -0.126 – 0.096 | -0.435 – 0.331 | 0.780 | 23.049 |
| SES | -0.038 | 0.181 | -0.037 | 0.175 | -0.413 – 0.337 | -0.398 – 0.325 | 0.836 | 23.167 |
| Other health conditions | -0.218 | 0.430 | -0.219 | 0.433 | -1.107 – 0.671 | -1.116 – 0.677 | 0.617 | 23.102 |
| Time X Condition | 0.289 | 0.344 | 0.292 | 0.347 | -0.421 – 1.000 | -0.424 – 1.008 | 0.409 | 24.160 |
| Time X Prior Camping | 0.658 | 0.433 | 0.664 | 0.437 | -0.235 – 1.551 | -0.237 – 1.564 | 0.141 | 24.831 |
| Condition X Prior Camping | -1.079 | 0.926 | -1.088 | 0.934 | -2.973 – 0.815 | -2.997 – 0.822 | 0.254 | 28.922 |
| Time X Condition X Prior Camping | -1.226 | 0.526 | -1.236 | 0.531 | -2.310 – -0.141 | -2.329 – -0.142 | **0.028** | 24.581 |
| **Random Effects** | | | | | | | | |
| σ^2^ | 0.14 | | | | | | | |
| τ_00_ _ID_Matching_ | 0.80 | | | | | | | |
| ICC | 0.85 | | | | | | | |
| N _ID_Matching_ | 32 | | | | | | | |
| Observations | 60 | | | | | | | |
| Marginal R^2^ / Conditional R^2^ | 0.229 / 0.883 | | | | | | | |

**Table S13.16**

*Results of Linear Mixed Model Predicting CDPQOL: Isolation (Ages 13-16 years) from Prior Camping, Condition, and Time, Controlling for Covariates*

|  | **CDPQOL: Isolation (Ages 13-16 years)** | | | | | | | |
| --- | --- | --- | --- | --- | --- | --- | --- | --- |
| *Predictors* | *B* | *SE* | *β* | *Std. SE* | *CI* | *Std. CI* | *p* | *df* |
| (Intercept) | 0.381 | 2.020 | -0.215 | 0.602 | -3.797 – 4.559 | -1.453 – 1.023 | 0.852 | 25.856 |
| Time | -0.211 | 0.214 | -0.345 | 0.350 | -0.651 – 0.229 | -1.065 – 0.375 | 0.333 | 24.783 |
| Condition | -0.118 | 0.439 | -0.193 | 0.719 | -1.016 – 0.780 | -1.662 – 1.276 | 0.791 | 29.431 |
| Prior Camping | -0.106 | 0.523 | -0.173 | 0.856 | -1.166 – 0.955 | -1.908 – 1.563 | 0.841 | 36.855 |
| Gender (1 = female) | 0.300 | 0.308 | 0.491 | 0.503 | -0.337 – 0.937 | -0.551 – 1.533 | 0.340 | 22.635 |
| Age | 0.077 | 0.122 | 0.128 | 0.202 | -0.174 – 0.329 | -0.289 – 0.545 | 0.531 | 23.099 |
| Age of diagnosis | 0.003 | 0.036 | 0.016 | 0.202 | -0.072 – 0.077 | -0.402 – 0.434 | 0.937 | 23.074 |
| SES | 0.041 | 0.122 | 0.065 | 0.191 | -0.211 – 0.294 | -0.330 – 0.460 | 0.737 | 23.282 |
| Other health conditions | -0.100 | 0.290 | -0.163 | 0.474 | -0.699 – 0.499 | -1.143 – 0.817 | 0.734 | 23.172 |
| Time X Condition | 0.273 | 0.339 | 0.447 | 0.555 | -0.426 – 0.973 | -0.697 – 1.591 | 0.428 | 24.341 |
| Time X Prior Camping | 0.666 | 0.424 | 1.089 | 0.694 | -0.207 – 1.539 | -0.338 – 2.517 | 0.129 | 25.665 |
| Condition X Prior Camping | 0.087 | 0.663 | 0.142 | 1.084 | -1.260 – 1.433 | -2.061 – 2.344 | 0.897 | 34.267 |
| Time X Condition X Prior Camping | -0.658 | 0.516 | -1.076 | 0.845 | -1.721 – 0.405 | -2.815 – 0.663 | 0.214 | 25.162 |
| **Random Effects** | | | | | | | | |
| σ^2^ | 0.14 | | | | | | | |
| τ_00_ _ID_Matching_ | 0.32 | | | | | | | |
| ICC | 0.70 | | | | | | | |
| N _ID_Matching_ | 32 | | | | | | | |
| Observations | 60 | | | | | | | |
| Marginal R^2^ / Conditional R^2^ | 0.074 / 0.719 | | | | | | | |

**Table S13.17**

*Results of Linear Mixed Model Predicting SWB from Prior Camping, Condition, and Time, Controlling for Covariates*

|  | **SWB** | | | | | | | |
| --- | --- | --- | --- | --- | --- | --- | --- | --- |
| *Predictors* | *B* | *SE* | *β* | *Std. SE* | *CI* | *Std. CI* | *p* | *df* |
| (Intercept) | 10.184 | 1.219 | -0.151 | 0.236 | 7.764 – 12.603 | -0.619 – 0.317 | **<0.001** | 107.391 |
| Time | 0.159 | 0.185 | 0.103 | 0.119 | -0.208 – 0.527 | -0.134 – 0.339 | 0.391 | 88.645 |
| Condition | 0.328 | 0.431 | 0.211 | 0.277 | -0.525 – 1.182 | -0.338 – 0.760 | 0.448 | 123.259 |
| Prior Camping | -0.565 | 0.653 | -0.363 | 0.420 | -1.858 – 0.728 | -1.195 – 0.468 | 0.389 | 123.929 |
| Gender (1 = female) | 0.177 | 0.321 | 0.114 | 0.206 | -0.460 – 0.815 | -0.296 – 0.524 | 0.582 | 92.978 |
| Age | -0.204 | 0.076 | -0.274 | 0.101 | -0.354 – -0.054 | -0.475 – -0.073 | **0.008** | 92.878 |
| Age of diagnosis | -0.035 | 0.048 | -0.067 | 0.093 | -0.131 – 0.061 | -0.252 – 0.118 | 0.474 | 92.957 |
| SES | 0.149 | 0.119 | 0.117 | 0.093 | -0.087 – 0.385 | -0.069 – 0.302 | 0.214 | 93.265 |
| Other health conditions | -0.139 | 0.348 | -0.089 | 0.224 | -0.831 – 0.553 | -0.534 – 0.356 | 0.691 | 93.777 |
| Time X Condition | -0.275 | 0.299 | -0.177 | 0.193 | -0.870 – 0.320 | -0.560 – 0.206 | 0.361 | 89.229 |
| Time X Prior Camping | -0.791 | 0.458 | -0.509 | 0.295 | -1.701 – 0.119 | -1.094 – 0.076 | 0.088 | 89.508 |
| Condition X Prior Camping | 0.565 | 0.777 | 0.364 | 0.500 | -0.973 – 2.104 | -0.626 – 1.353 | 0.468 | 124.862 |
| Time X Condition X Prior Camping | 0.834 | 0.543 | 0.537 | 0.350 | -0.246 – 1.914 | -0.158 – 1.231 | 0.128 | 89.497 |
| **Random Effects** | | | | | | | | |
| σ^2^ | 0.53 | | | | | | | |
| τ_00_ _ID_Matching_ | 1.76 | | | | | | | |
| ICC | 0.77 | | | | | | | |
| N _ID_Matching_ | 102 | | | | | | | |
| Observations | 193 | | | | | | | |
| Marginal R^2^ / Conditional R^2^ | 0.122 / 0.795 | | | | | | | |

# Evaluation: feasibility and acceptability of IG Zöliakie Camps

## Future activities preferences among all study participants

Participants were asked in the first questionnaire (pretest) about activities they would like Zölikids to offer. The following figure presents the extent to which children aged 8–12 years would like to take part in four types of activities:

The figure shows that the most popular activity is a zoo visit (Zoobesuch), followed by a treasure hunt (Schatzsuche) and candle making (Kerzenziehen). In an open-ended question, participants suggested a variety of other activities they would like to participate in:

***Do you have other suggestions for Zölikids events?***

- *Baking, cooking, bowling*
- *Baking course at Jovis Bakery in Bern*
- *Baking courses*
- *Baking and cooking courses*
- *Swimming in the afternoon (Badi-nami)*
- *Biking, cross-country skiing, playing ping-pong*
- *Bike tours*
- *Playing ice hockey*
- *Cycling tour, swimming, cooking, baking*
- *Soccer tournament*
- *Soccer tournament, camping*
- *Rock climbing together*
- *Movie visit*
- *Going to the cinema*
- *Cooking class for teenagers*
- *Cooking, baking*
- *Painting in a studio, baking, games afternoon*
- *Museum visit, cooking & baking*
- *Nature conservation activities, e.g., weeding*
- *Orienteering*
- *Horse riding*
- *Riding camp*
- *Swimming, horse riding*
- *Sunday brunch - taking the train to visit various bakeries and possibly baking together in the afternoon*
- *Sports day*
- *Sports afternoon, crafts afternoon*
- *Celiac party*
- *Celiac party / crafts afternoon*
- *Zölikids cooking course, baking course*
- *Cooking together*
- *Cooking and hiking together.*

**The following figure presents the distribution of preferences for six types of activities offered to adolescents aged 13–16 years:**

#

As shown in the diagram, adolescents are most interested in *rope parks* (*Seilpark*) and *baking courses* (*Backkurs*), followed by *cooking courses* (*Kochkurs*) and *candle making* (*Kerzenziehen*). Unlike children (ages 8-12 years), who favor the zoo visit, this activity is the least popular among adolescents (ages 13-16 years).

**Here are the suggestions from adolescents for activities they are interested in:**

**Do you have other suggestions for Zölikids events?**

1. **Game day**
2. **Eating together in a restaurant**
3. **A dessert competition**
4. **Aquabasilea, Alpamare, Europa Park**
5. **International trip**
6. **Activities where celiac disease isn’t always the focus, just to have fun**
7. **Escape room**
8. **Spring camp**
9. **Sleeping outdoors for a weekend, cooking and grilling ourselves**
10. **Shopping**
11. **Skillspark Winterthur**
12. **Helping out at an animal shelter**
13. **Hiking or cycling tour**

*.*

## Camp evaluation: Expectations before the camp

Zölikids-Lager participants were asked in the pretest questionnaire to rate their expectations for the summer camp. The figure below shows that overall, more than 80% of the campers were highly anticipating the camp experience in general, as well as the availability of gluten-free food. Additionally, slightly more than half of the participants were very eager to meet other children at the camp. In contrast, just under half of the children expressed strong interest in meeting and getting to know the camp supervisors (*Betreuer*).

**Satisfaction with the camp (posttest)**

In the post-test questionnaires, completed approximately four weeks after the camp, participants rated their satisfaction with various aspects of the camp, including the camp experience overall, the food and facilities, the activities, friendships formed, and the supervision (*Betreuung*). The distribution of responses is shown in the following figure:

Most camp participants expressed high satisfaction from the cam and the supervision, as well as from the food, activities, and friends they met in the course. It should be noted that for most campers, satisfaction was high (“zufrieden”) but not very high (“zehr Zufrieden”). However, about one in three campers expressed neutral or low satisfaction from the Unterbringung in the camp, and about one of five campers was not satisfied from the activities, the food or other participants.

The following table presents average levels of satisfaction on a scale of 1 to 5 (and standard deviations) for participants based on age groups and Zölikids lager for each aspect for which satisfaction was measured. Results of a series of independent sample t-tests show that satisfaction from the camp in general, as well as from the Betreuung and the social aspect of the camp was significantly higher among adolescent compared to child participants. Moreover, average levels of satisfaction seem to be somewhat lower for Sommerlager 2 compared to Sommerlager 1, although not significantly.

| Satisfaction aspect | \| **Age Group 8–12 (n=35)** \| \| --- \| | \| **Age Group 8–12 (n=35)** \| \| --- \| | \| **Age Group 8–12 (n=35)** \| \| --- \| | \| **Age Group 8–12 (n=35)** \| \| --- \| | \| **Age Group 8–12 (n=35)** \| \| --- \| |
| --- | --- | --- | --- | --- | --- | --- | --- | --- | --- | --- |
| \| The summer camp overall \| \| --- \|  \|  \| \| --- \| | 3.86 (0.43) | 4.38 (1.02)** | 4.19 (0.91) | 3.80 (0.41) | 4.17 (0.41) |
| \| Food at the summer camp \| \| --- \|  \|  \| \| --- \| | 3.79 (0.41) | 4.14 (1.15) | 4.03 (0.95) | 3.79 (0.54) | 4.00 (0.63) |
| \| Accommodation (e.g., sleeping arrangements) \| \| --- \|  \|  \| \| --- \| | 3.74 (0.61) | 3.62 (1.07) | 3.65 (0.95) | 3.90 (0.55) | 3.33 (0.52) |
| \| Activities at the summer camp \| \| --- \|  \|  \| \| --- \| | 3.74 (0.44) | 4.00 (1.10) | 3.87 (0.92) | 3.65 (0.59 | 4.00 (0.63) |
| \| Supervision at the summer camp \| \| --- \|  \|  \| \| --- \| | 3.89 (0.40) | 4.43 (0.98)** | 4.28 (0.82) | 3.85 (0.49) | 4.00 (0.63) |
| \| Other children met at the camp \| \| --- \|  \|  \| \| --- \| | 3.76 (0.50) | 4.30 (0.98)** | 4.14 (0.92) | 3.74 (0.56) | 4.00 (0.00) |

* p < .05, ** p < .01, *** p < .001

Satisfaction with the camp was also analyzed based on gender and camp experience, comparing new campers (those attending for the first time) with returning campers (those who had previously attended at least one camp). Overall, there were no significant differences in satisfaction levels between boys and girls or between new and returning campers.

| Satisfaction Aspect | Male (n=21) | Female (n=35) | New Campers (n=18) | Returning Campers (n=39) |
| --- | --- | --- | --- | --- |
| The summer camp overall | 4.00 (0.89) | 4.09 (0.66) | 4.00 (0.59) | 4.08 (0.81) |
| Food at the summer camp | 3.95 (0.81) | 3.91 (0.79) | 4.00 (0.59) | 3.92 (0.88) |
| Accommodation (e.g., sleeping arrangements) | 3.86 (0.85) | 3.60 (0.78) | 3.83 (0.62) | 3.64 (0.87) |
| Activities at the summer camp | 3.81 (0.87) | 3.86 (0.69) | 3.89 (0.68) | 3.77 (0.84) |
| Supervision at the summer camp | 4.00 (0.89) | 4.14 (0.60) | 3.94 (0.64) | 4.18 (0.76) |
| Other children met at the camp | 3.80 (0.95) | 4.06 (0.61) | 3.89 (0.58) | 4.03 (0.85) |

**The following figure presents the mean scores for overall satisfaction with the camp on a scale from 1 to 10:**

Consistent with the results of the feedback questionnaire, participants reported a high level of enjoyment from the camp, with no significant differences observed between age groups, gender, new or returning campers, or the specific camp attended. While average enjoyment was slightly higher in the Herbstlager and lower in Sommerlager 1, these differences were not statistically significant.

Overall satisfaction with the camp is also reflected in the very high willingness of participants to recommend the camp to other children with celiac disease. As shown in the following figure, nearly all campers would recommend children their age with celiac to attend the camps:

Finally, the high satisfaction with the camp is also reflected in the participants' strong intention to return next year. As shown in the following figure, the vast majority of children indicated that they would register for the camp again. Interestingly, all boys (100%) reported that they intend to return to the camp ("ja"), compared to 77% of the girls.

## Friendship formation during the camp:

Children who attended Zölikids-Lager were asked if they made new friends during the camp. The following figure presents the results for all campers, as well as by age group, gender, camp experience (new or returning campers), and the specific camp attended. The results are overwhelmingly positive—almost all participants made at least one new friend, with many reporting the formation of multiple friendships. While friendship formation appears to be slightly stronger among boys, new campers, and participants of the Herbstlager, high rates of friendship were observed across all groups.

## Potential for long-term friendships:

The following figure presents the distribution of responses to the question, “Do you think you will stay in contact with the other children from Zölikids-Lager in the future?” These responses may indicate the expected or potential for long-term positive social impacts of the camps.

Most campers expressed expectations of staying in contact with others they met at the camp. These expectations were higher among adolescent campers (52% responded “yes”) compared to child campers (only 17% were certain). Additionally, expectations for continued interactions were slightly higher among girls (37%) than boys (19%). Interestingly, new campers were more optimistic about maintaining friendships (61%) compared to returning campers (45%). Responses also varied by camp session: half of the participants from the first summer camp expected to stay in touch, compared to only 5% from the second summer camp. The results for the Autumn camp should be interpreted with caution, as only six participants were included in the study. Overall, based on self-reports, there appears to be a reasonable likelihood for long-term friendships among more than half of the campers.

## The camp facilitator as a role model:

To what extent did participants in Zölikids-Lager view the camp leaders (Lagerleiterinnen) as role models, and how many expressed interest in becoming camp leaders themselves in the future? The following figure shows the mean scores on a scale from 1 (“not at all”) to 5 (“very much”).

Among all campers, 21 (approximately 40%) indicated that they would “very much” like to become a Zölikids-Lagerleiter*in in the future. Notably, almost half (47%) of female participants expressed a strong desire to take on this role, compared to only 21% of male participants. Interest in becoming a camp leader was also slightly higher among returning campers (44%) compared to new campers (28%). Additionally, the percentage of campers who were highly interested in leading camps was higher among participants from the first summer camp (45%) and the autumn camp (50%) compared to those from the second summer camp (26%).

## Self-assessment of camp’s impact on coping:

Participants were asked whether they believe the camp helped them better cope with their disease and manage it in their daily lives. The mean scores, on a scale from 1 to 5, are presented in the following figure:

Among all campers, only 25% reported that the camp helped them "very much" in managing their disease in their daily lives. Perceived help was slightly higher among child participants (30%) compared to adolescents (20%), and also among new campers (33%) compared to returning campers (22%). There was also a difference between the camps: the percentage of children who attended Sommerlager 2 and found the camp helpful for managing their celiac disease was lower (10%) than those from Sommerlager 1 (27%) and Herbstlager (67%).

.

## Answers to open questions:

In the final section of the feedback questionnaire, camp participants were asked to briefly respond to three open-ended questions. The first question was: *"What did you like most about the Zölikids camp?"* The answers are presented in the following table, categorized by the camp attended, age group, and gender of the participants:

**Zölikids-Lager: *"What did you like most about the Zölikids camp?"***

Responses are categorized by camp, age group, and gender.

**1. Sommerlager 1**

- **Children aged 8–12 years**
  - **Male**:
    - Everything
    - Playing ping pong and the food
    - Everything
  - **Female**:
    - Being able to eat everything, meeting people with the same illness, just everything!
    - The food...
    - The food and spending time together
    - The food, the children, and the kind supervisors! I liked everything!
    - The delicious food
    - The friends
    - The water fight, hiking
    - Food
    - Meeting everyone again
    - Everything
- **Adolescents aged 13-16 years**
  - **Male**:
    - Activities, food, meeting new people, seeing old friends again
    - The food
    - The food and the games
    - The hike
    - Making friends
    - Playing ping pong with others
    - Everything
  - **Female**:
    - In general, all the funny moments
    - The food
    - That I met new people
    - That I was in a room with amazing people and the leadership team
    - The other kids
    - The people
    - All the friends
    - Food, spending time together
    - Spending free time with friends
    - Meeting friends from last year again
    - Singing and variety
    - Hiking
    - Everything

**2. Sommerlager 2**

- **Children aged 8-12 years:**
  - **Male**:
    - Everything
    - The food
    - The food (especially the Berliner), the great trips, the kids
    - The same food for everyone. The trips/hikes. Games...
    - The theme (Time Travel) and the food
    - The kids and supervisors
    - Food
    - Food and friends
    - My room, the delicious food
    - Many kids without parents, food
    - The hike
    - The hike
    - Everything
  - **Female**:
    - The food, the trips, sleeping in the room with the other children
    - That you always meet new friends
    - Food
    - Swimming pool
    - Doing things together
    - Doing things together
    - Making waffles and eating them
    - Everything
- **Adolescents aged 13-16 years:**
  - **Female**:
    - The beauty night

**3. Herbstlager**

- **Children aged 8-12 years:**
  - **Male**:
    - The hike
  - **Female**:
    - The food, the activities
    - The games and just feeling normal
    - The games. That I could eat anything without asking. It was paradise.
    - Being like the others and being able to eat everything
    - Everything
- **Adolescents 13-16 years:**
  - **Female**:
    - The night hike

**The second open question was: "Was there anything you did not like about the Zölikids camp? If so, what was it?"** The answers are presented in the following table, categorized by camp, age group, and gender.

**Zölikids-Lager: *"Was there anything you did not like about the Zölikids camp? If so, what was it?"***

Responses are categorized by camp, age group, and gender.

**1. Sommerlager 1:**

- **Children aged 8-12 years:**
  - **Male**:
    - No
  - **Female**:
    - That we could hear the people in the next room very loudly.
    - The night hike was a bit too cold! But the time was perfect. It could even be at 1 or 2 AM! ;)
    - The homemade gummy bears
    - Homesickness
    - The horror hike (it was very exhausting)...
    - The camp Olympics
    - Bullying
    - No
- **Adolescents aged 13-16 years:**
  - **Male**:
    - Nothing
    - Night activity
    - No
    - Nothing
  - **Female**:
    - The food was too unhealthy.
    - That my best friend wasn’t in the same camp.
    - The toilets.
    - A scarier night hike.
    - Sometimes there were too many “activities for little kids.”
    - I found that the program was designed more for younger kids, even though I was in the camp for older children.
    - No
    - Water fight, night activity, and show performance
    - The weekly theme (Time Travel) was no longer age-appropriate in my opinion.

**2. Sommerlager 2:**

- **Children aged 8-12 years:**
  - **Male**:
    - One kid in my room always argued with me.
    - It was too short... otherwise, nothing.
    - No
    - No
    - Nothing
    - Nothing
  - **Female**:
    - Fried apple rings
    - No
    - No
    - Sleeping
    - The hike
- **Adolescents aged 13-16 years:**
  - **Female**:
    - The time travel theme because I was a bit too old for it.

**3. Herbstlager:**

- **Children aged 8-12 years:**
  - **Male**:
    - Nothing
  - **Female**:
    - No
    - No, I liked everything very much.
    - Nothing
    - Nothing
- **Adolescents aged 13-16 years:**
  - **Female**:
    - No

Finally, campers were asked: "Is there anything you would add or change about the Zölikids camp?" The responses to this question are presented in raw form in the table below:

**Zölikids-Lager: *"Is there anything you would add or change about the Zölikids camp?"***

Responses are categorized by camp, age group, and gender.

**1. Sommerlager 1:**

- **Children aged 8-12 years**
  - **Male**:
    - No
  - **Female**:
    - Everyone going to bed at the same time
    - More excursions! Or a long, long night hike to observe animals
    - Healthier food
    - No
    - Nothing
- **Adolescents aged 13-16 years**
  - **Male**:
    - A two-week camp
    - More intense night activities
    - Nothing
  - **Female**:
    - The program
    - Let us cook for one day
    - Movie night
    - Group assignments
    - I think there should be a camp for 12 and older due to the activities
    - I would offer healthier snacks
    - No water fight, no scary night activity, and no show performance
    - The camp for older kids should start at age 13
    - No

**2. Sommerlager 2:**

- **Children aged 8-12 years**
  - **Male**:
    - Learn to cook
    - The camp location was too far from home. The travel distance was long for both drop-off and pick-up.
    - More hiking
    - No
    - Nothing
    - More activities, less free time
  - **Female**:
    - Let the children help with cooking one time
    - No
    - Nothing
    - Zölikids camp on a horse farm :)
- **Adolescents aged 13-16 years**
  - **Female**:
    - A scary night in both camps

**3. Herbstlager:**

- **Children aged 8-12 years**
  - **Male**:
    - No
  - **Female**:
    - Offer the Zölikids camp as a ski camp
    - Do something with animals (like trekking)
    - No
- **Adolescents aged 13-16 years**
  - **Female**:
    - No
